# Non-coding RNAs and Mitochondrial Dysfunction in Alzheimer’s Disease: A Systematic Review

**DOI:** 10.1007/s12035-026-06198-9

**Published:** 2026-09-15

**Authors:** Ștefania-Maria Mocrei-Rebrean, Vlad Răzniceanu, Sebastian Romeo Pintilie, Ioana Berindan-Neagoe

**Affiliations:** 1 Faculty of Medicine, Iuliu Hațieganu University of Medicine and Pharmacy, Cluj-Napoca, Romania; 2 Department of Genomics, MEDFUTURE Institute for Biomedical Research, Iuliu Hațieganu University of Medicine and Pharmacy, Cluj-Napoca, Romania; 3 Doctoral School, Iuliu Hațieganu University of Medicine and Pharmacy, Cluj-Napoca, Romania; 4 Academy of Medical Sciences, Bucharest, Romania

**Keywords:** Alzheimer’s disease, Mitochondrial dysfunction, Non-coding RNA, MicroRNA, Long non-coding RNA, Circular RNA

## Abstract

Alzheimer’s disease (AD) is responsible for 70% of dementia cases worldwide, with tau hyperphosphorylation and amyloid-β plaque accumulation representing its core pathological hallmarks. Genetic predisposition, oxidative stress, and neuroinflammation contribute to disease onset and progression. Non-coding ribonucleic acids (ncRNAs) are a class of RNAs which control gene expression and whose dysregulation in AD patients has been linked to amyloid production, neuroinflammation, and mitochondrial dysfunction, which ranges from impaired energy metabolism to disrupted mitochondrial biogenesis and dynamics. Our descriptive systematic review surveyed the involvement of ncRNAs in mitochondrial dysfunction in AD across experimental and clinical literature. We identified multiple microRNAs (miRNAs), long non-coding RNAs (lncRNAs), and circular RNAs (circRNAs) that directly regulate mitophagy, mitochondrial biogenesis, mitochondrial autophagic, and apoptotic pathways, mitochondrial dynamics, and protein import mechanisms in AD models. Among the most important candidates demonstrating clinical dysregulation, miR-140 and lncRNA NEAT1 regulate mitophagy, while miR-9, miR-34a, miR-146a, miR-155, and miR-485 are implicated in mitochondrial biogenesis and miR-204 in mitochondrial autophagy. LncRNA BDNF-AS, miR-148a-3p, miR-21-5p, and miR-103a-3p emerged as regulators of the mitochondrial apoptosis pathway with confirmed clinical dysregulation. Multiple ncRNAs control mitochondrial dynamics, of which miR-195, miR-124, and miR-455-3p have also been studied in AD patients. Additionally, several ncRNAs were found to indirectly regulate mitochondrial fission, autophagy, and apoptosis, although the underlying mechanisms require further characterization. Thus, while ncRNA-centered AD research is in its early stages, current mechanistic and translational evidence supports mitochondrially relevant ncRNAs as promising candidates for biomarker and therapeutic development.

**Supplementary Information:**

The online version contains supplementary material available at https://doi.org/10.1007/s12035-026-06198-9.

## Introduction

Alzheimer’s disease (AD) accounts for 70% of dementia cases worldwide [1], with 58 million cases documented as of 2020 and an estimated threefold increase in prevalence by 2050 [2]. AD is characterized by tau hyperphosphorylation [3], amyloid-beta (Aβ) plaque buildup [4, 5], and synaptic failure [6]. Additional pathophysiological contributors include oxidative stress [7], neuroinflammation [8], and mutations in the genes β-amyloid precursor protein (APP), presenilin 1 (PSEN1), presenilin 2 (PSEN2), and apolipoprotein E (APOE) [9]. Early diagnosis and disease-modifying treatment in AD are of great research interest in order to reduce disability and to alleviate healthcare system burden [10, 11].

**Fig. 1:**
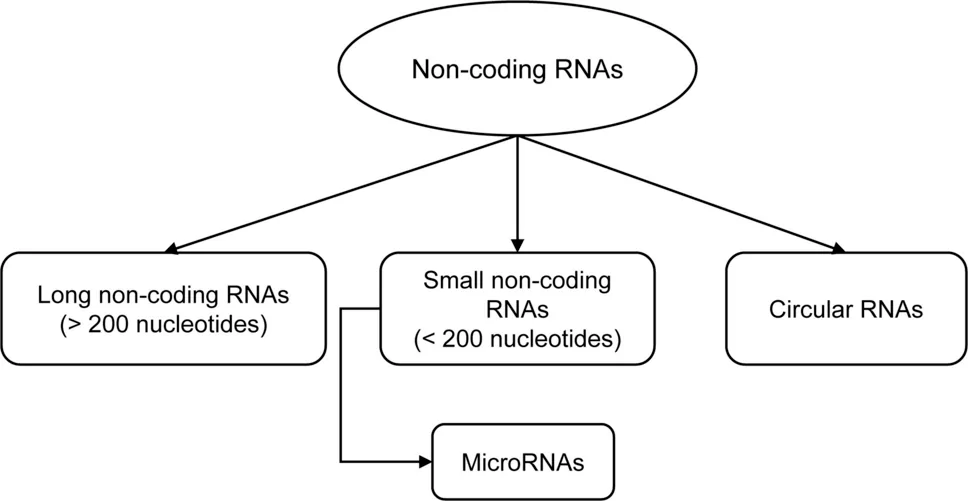
Main non-coding RNA classes relevant to mitochondrial dysfunction in Alzheimer’s disease

Non-coding RNA (ncRNA) is a type of RNA which is not translated into proteins, but instead helps regulate gene expression [12]. As shown in Fig. 1, ncRNAs can be classified into long non-coding RNAs (lncRNA) and small non-coding RNAs by using a threshold of 200 nucleotides [13–16]. MicroRNAs (miRNAs) belong to the latter class [17] while circular RNAs (circRNAs) are a distinct category of ncRNAs with a closed-loop structure [18, 19]. LncRNAs induce various epigenetic modifications [20–22]. By contrast, miRNAs bind to complementary mRNA sequences to inhibit their translation [23, 24] and circRNAs act as a “sponge” for miR, thereby preventing it from repressing target messenger RNA (mRNA) [25]. LncRNAs influence amyloid production, tau hyperphosphorylation, mitochondrial dysfunction, oxidative stress, and synaptic impairment [26]. Accumulating evidence indicates that ncRNAs play an important role in AD-related oxidative stress [27, 28] and that AD may benefit from the usage of ncRNAs as biomarkers and therapeutic targets [29, 30].

Alterations in mitochondrial biogenesis, dynamics, mitophagy, proteostasis, and mitochondrial DNA (mtDNA) have been observed in AD [31]. However, it is unclear whether mitochondrial defects precede or are a consequence of amyloid pathology [32]. On the one hand, Aβ and hyperphosphorylated tau alter neuronal energy production and antioxidant defense mechanisms [31], which subsequently disturbs mitochondrial physiology, cytochrome oxidase activity, and intracellular calcium homeostasis [32]. On the other hand, mitochondrial reactive oxygen species (ROS) favor Aβ buildup [33].

Although numerous reviews analyze the role of ncRNAs in AD pathogenesis in the general sense [28, 34–37], there is a scarcity of literature summarizing the data available on ncRNA involvement in mitochondrial dysfunction in AD. To our knowledge, only one narrative review published in 2024 has investigated the mechanistic links between the various ncRNA classes, amyloid pathology and mitochondrial dysfunction in AD [38]. Three other studies reviewed the role of mitochondrially-localized microRNAs in AD pathology [39–41]. The purpose of this paper is to provide a systematic and updated overview on the state of ncRNA research in the field of AD with a focus on mitochondrial dysfunction and potential therapeutic and diagnostic applications.

## Methods

### Search Strategy

A systematic literature search of the PubMed, Embase, and Web of Science Core Collection databases was conducted using search strings combining ‘Alzheimer’ and ‘mitochondrial’ with Boolean operators and the following ncRNA terms: ‘microRNA’, ‘miRNA’, ‘miR’, ‘long non-coding RNA’, ‘lncRNA’, ‘non-coding RNA’, ‘circular RNA’, ‘circRNA’, ‘small nucleolar RNA’, ‘snoRNA’, ‘short hairpin RNA’, ‘small hairpin RNA’ and ‘shRNA’. Studies published before 18 November 2025 were assessed for eligibility.

### Study Eligibility Criteria

Articles investigating the pathological mechanisms, therapeutic or biomarker applications of the selected ncRNA classes, or their regulatory enzymes in patients or experimental AD models were included. The inclusion criteria were (1) studies written in English; (2) research conducted on human patients or experimental AD models; (3) studies investigating endogenous ncRNAs or synthetic ncRNA-based reagents that mimic in vivo processes in relation to mitochondrial parameters in AD; (4) investigations addressing the pathological mechanisms of ncRNAs or their regulatory enzymes in relation to mitochondrial dysfunction in AD; (5) investigations evaluating therapeutic applications of the selected ncRNA classes or their regulatory enzymes in relation to mitochondrial function in AD; (6) investigations evaluating the biomarker applications of the selected ncRNA classes or their regulatory enzymes in AD; and (7) original research articles. The exclusion criteria were (1) conference/meeting abstracts; (2) non-English publications; (3) reviews and meta-analyses; (4) bibliometric analyses and editorials; (5) articles investigating diseases other than AD; (6) articles whose primary outcomes were unrelated to ncRNA expression or ncRNA biogenesis in AD; (7) articles whose primary outcomes included mitochondrial parameters but not ncRNAs or ncRNA biogenesis in AD; (8) articles whose models induced cognitive decline via secondary AD pathogenetic mechanisms without reproducing essential histopathological characteristics of AD (e.g., chemically induced AD models via scopolamine, AlCl_3_ and streptozotocin [42]).

### Data Extraction

Screening was performed independently by three reviewers (S.M.M.R., V.R. and S.R.P.). Conflicts were resolved by a fourth experienced reviewer (I.B.N.). The extracted data were organized into tables featuring publication details, experimental model, patient cohort or bioinformatics dataset, sample type, RNA quantification method, and mitochondria-relevant results. Given the heterogeneity of models, outcomes, and sample sizes, a meta-analysis and risk of bias assessment were not performed.

## Results and Discussion

### Database Screening and Characteristics of the Included Studies

**Fig. 2:**
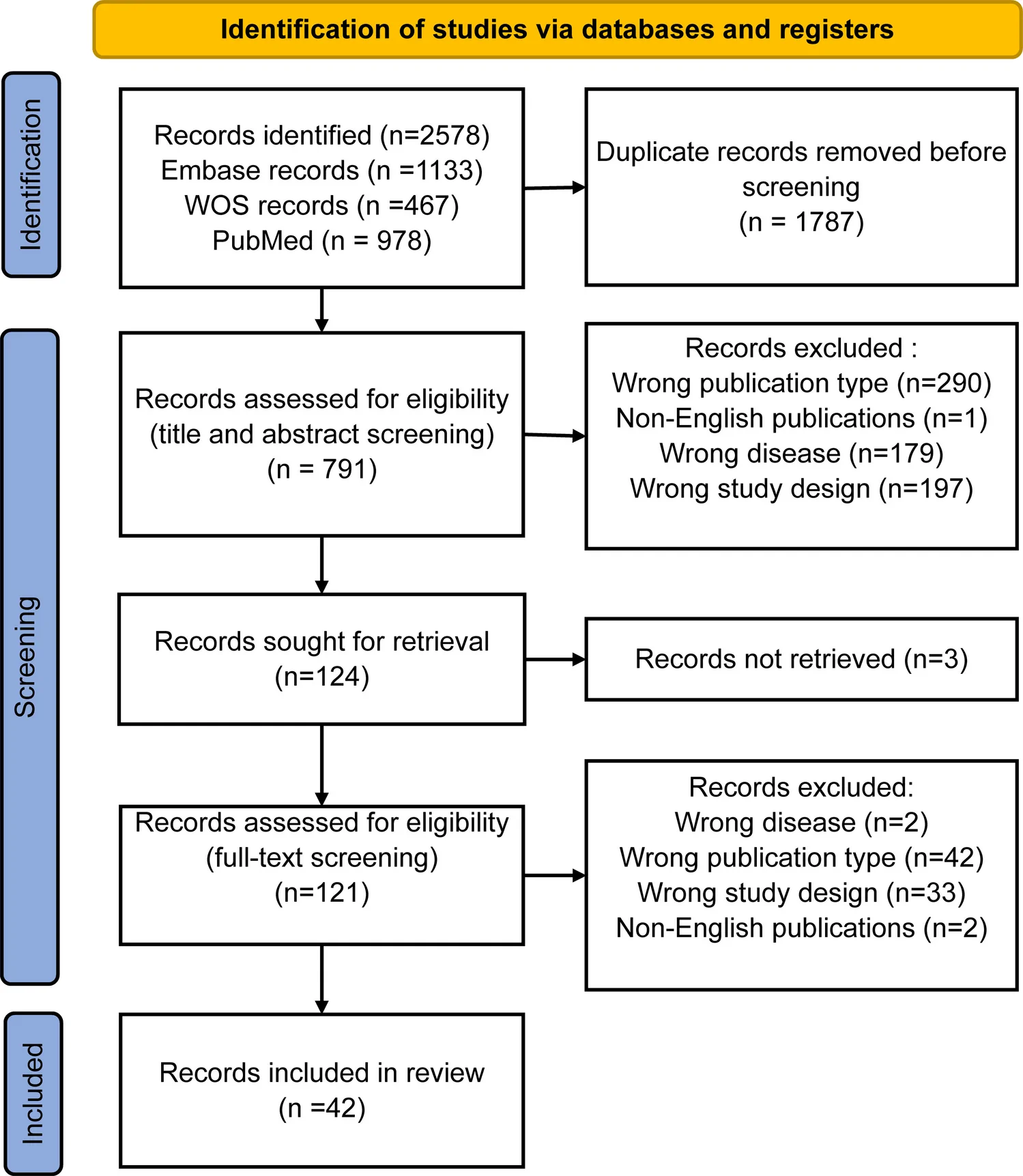
PRISMA flowchart illustrating the identification and screening process of the studies included in this review

Article identification and selection following PRISMA guidelines [43] are summarized in Fig. 2. PubMed, Embase, and Web of Science Core Collection searches retrieved 2578 records. After automatic duplicate identification using Rayyan [44] and manual assessment, 1787 duplicates were removed. Screening yielded 42 studies, an overview of which is provided in Table 1.

**Table 1:** Overview of studies included in this systematic review: design, models, and main ncRNA findings

No.	Reference	Year	Study type	Model and sample type	RNA quantification methods	NcRNA class studied	Primary objective
1.	[45]	2019	Fundamental	Cell-based model	RT-qPCR	miRNA lncRNA	Aβ25–35 ↑ SNHG1; SNHG1 knockdown restores MMP and reduces apoptosis via the miR-137/KREMEN1 axis
2.	[46]	2020	Mixed^a^	Human (brain) Rodent (brain) Cell-based model	RT-qPCR	miRNA lncRNA	↓ WT1-AS and ↑ miR-375 in AD; WT1-AS overexpression increases MMP/ATP and reduces oxidative stress via the WT1/miR-375/SIX4 axis
3.	[47]	2021	Fundamental	Rodent (brain) Cell-based model	RT-qPCR	lncRNA miRNA	Aβ25–35 ↑ H19 and ↓ miR-129; H19 silencing restores MMP and reduces oxidative stress via the miR-129/HMGB1 axis
4.	[48]	2025	Fundamental	Cell-based model	RT-qPCR	miRNA circRNA	Aβ ↑ circ_0049472 and ↓ miR-22-3p; circ_0049472 knockdown restores mitochondrial function via the miR-22-3p/PDE4A axis
5.	[49]	2023	Fundamental	Cell-based model	Poly(A) and total RNA-seq	lncRNA circRNA	PSEN1 A79V/L150P neurons: ↓ MEG3, LOXL1-AS1, A2M-AS1; CircRNAs predominantly ↑; ↑ CDR1as/circ_0001946 in L150P
6.	[50]	2020	Fundamental	Rodent (brain) Cell-based model	RT-qPCR	miRNA	APP/PS1 mice/Aβ42: ↓ Dicer1 via Nrf2 repression; Dicer1 loss increases oxidative stress and decreases MMP; Dicer1 overexpression is neuroprotective
7.	[51]	2022	Translational^a^	Human (brain)	N/A	miRNA	↓miR-132-3p, miR-212-3p in AD; miR-9-5p variably dysregulated in AD
8.	[52]	2025	Translational^a^	Human (brain)	N/A	miRNA lncRNA	↓ miR-129-5p and miR-132 in AD brains; Higher miR-129-5p/miR-132 associated with greater brain resilience; miR-124-3p, NEAT1 and PTEN emerged as key nodes in a mitochondrial-related ceRNA network
9	[53]	2025	Mixed^a^	Human (brain, blood) Cell-based model	N/A	miRNA	↓ HIBCH and MGME1 in AD; miR-922 directly targets MGME1; miR-98-5p does not
10.	[54]	2025	Translational^a^	Human (brain)	N/A	miRNA	miR-103 and miR-484 predicted to co-regulate AD-relevant OXPHOS and TCA-cycle genes
11.	[55]	2021	Translational	Human (brain)	RT-qPCR	miRNA	↑ miR-485 in AD frontal cortex; Associated with ↓ PGC-1α and impaired antioxidant response
12.	[56]	2024	Translational^a^	Human (brain)	N/A	miRNA	miR-106b-5p, miR-17-5p and miR-16-5p linked to altered protein networks in AD hippocampus; Protein alterations associated with impaired mitochondrial function
13.	[57]	2020	Translational	Human (brain)	miRNA array-based analysis	miRNA	↑ miR-9, miR-34a, miR-146a and miR-155 in AD temporal cortex; All target the SIRT1 3′UTR, consistent with ↓ SIRT1
14.	[58]	2021	Mixed	Human (plasma, CSF, brain) Cell-based model	miRNA microarray; RT-qPCR	miRNA	↑ miR-1273g-3p in AD plasma and CSF; miR-1273g-3p suppresses TIMM13, GLRX5 and MTCH1 and induces mitochondrial dysfunction
15.	[59]	2022	Translational	Human (brain)	RT-qPCR	miRNA	↑ miR-146a in AD temporal cortex miR-146a inversely associated with VDAC and impairs oxidative phosphorylation
16.	[60]	2024	Mixed	Human (brain) Rodent (brain)	RT-qPCR	miRNA	↓ miR-218-5p and miR-124-3p in AD hippocampus; Loss of miR-218-5p/miR-124-3p increases RTN3
17.	[61]	2024	Mixed	Human (brain) Rodent (brain) Cell-based model	miRNA microarray; RT-qPCR	miRNA	↑ miR-323-5p and miR-370-3p in AD-model mice; miR-150-3p, miR-323-5p and miR-370-3p ↓ OPA1; miR-150-3p directly targets MFN2
18.	[62]	2024	Translational^a^	Human (brain)	N/A	miRNA	AD-associated miRNAs predicted to target autophagy/mitochondrial biomarkers TFEB, TOMM20 and GABARAPL1
19.	[63]	2021	Fundamental	Rodent (brain) Cell-based model	RT-qPCR	miRNA	↑ miR-351-5p and ↓ Miro2 in AD-model hippocampus; miR-351-5p suppresses Miro2
20.	[64]	2024	Mixed	Human (brain) Rodent (brain) Cell-based model	RT-qPCR	miRNA	↑ miRNA PC-5P-12969 in AD brains and models; PC-5P-12969 targets GSK3α and improves mitochondrial respiration/ATP while reducing fragmentation
21.	[65]	2025	Fundamental	Rodent (brain) Cell-based model	Nanostring miRNA expression assay; RT-qPCR	miRNA	APP/PSEN1 mice: ↑ miR-7a, miR-148a. ↓ miR-34a, miR-128a; miR-7a targets Klf4, reducing ferroptosis-associated mitochondrial fragmentation and MMP loss
22.	[66]	2025	Fundamental	Rodent (brain) Cell-based model	RT-qPCR	miRNA	↑ miR-9-5p and ↓ SIRT1 in APP/PS1 hippocampus; Rg1 ↓ miR-9-5p, restores SIRT1, improves mitochondrial function/mitophagy
23.	[67]	2025	Fundamental	Cell-based model	RT-qPCR	miRNA	miR-148a-3p, miR-21-5p, miR-103a-3p derived from neural stem cells exert mitochondrial protective effects in AD
24.	[68]	2021	Fundamental	Rodent (brain) Cell-based model	RT-qPCR	miRNA	↑ miR-140 and ↓ PINK1 in AD models; miR-140 directly targets PINK1; miR-140 silencing improves mitochondrial function and autophagy
25.	[69]	2020	Fundamental	Cell-based model	RT-qPCR	miRNA	Aβ25–35: ↓ miR-9-5p and ↑ GSK-3β; miR-9-5p targets GSK-3β, reducing mitochondrial dysfunction and oxidative stress
26.	[70]	2016	Fundamental	Rodent (brain) Cell-based model	miRNA microarray; RT-qPCR	miRNA	SAMP8 hippocampus: ↑ miR-195, ↓ miR-15b; miR-195 directly targets MFN2, causing loss of MMP
27.	[71]	2021	Fundamental	Rodent (brain) Cell-based model	RT-qPCR	miRNA	↑ miR-204 and ↓ TRPML1 in AD models; miR-204 targets TRPML1, promoting ROS production and impaired mitochondrial autophagy
28.	[72]	2021	Fundamental	Cell-based model	RT-qPCR	miRNA	AD neuronal models: ↑ miR-124, miR-125b and miR-21; ↑ exosomal miR-124 and miR-155; miR-124 mimic improves mitochondrial membrane potential
29.	[73]	2022	Fundamental	Cell-based model	RT-qPCR	miRNA	↑ miR-124 in APP-Swedish neurons and their secretome; miR-124 mimic preserves mitochondrial membrane potential and reduces inflammatory activation
30.	[74]	2019	Fundamental	Cell-based model	RT-qPCR	miRNA	miR-455-3p promotes mitochondrial biogenesis and fusion (↓ DRP1/FIS1; ↑ OPA1/MFN1/MFN2), reducing mutant APP-induced mitochondrial fragmentation
31.	[75]	2024	Mixed	Human (brain) Rodent (brain) Cell-based model	RNAscope ISH; RT-qPCR; Northern blot	lncRNA	↓ lncMtDloop in human AD brains and 3xTg mice; lncMtDloop regulates TFAM-dependent mitochondrial transcription, improving mitochondrial homeostasis
32.	[76]	2018	Mixed	Human (CSF, serum) Cell-based model	RT-qPCR	lncRNA	↑ lncRNA-ATB in AD CSF/serum and Aβ-treated cells; lncRNA-ATB suppression restores MMP and reduces oxidative injury via the miR-200/ZNF217 axis
33.	[77]	2022	Fundamental	Rodent (brain) Cell-based model	RT-qPCR	lncRNA	↑ MIR600HG in AD mice; MIR600HG promotes NEDD4L-mediated PINK1 degradation, impairing autophagy and mitochondrial function
34.	[78]	2020	Fundamental	Rodent (brain) Cell-based model	RT-qPCR	lncRNA	↑ NEAT1 in AD mice NEAT1 promotes NEDD4L-mediated PINK1 ubiquitination, impairing PINK1-dependent autophagy
35.	[79]	2018	Fundamental	Cell-based model	RT-qPCR	lncRNA	Aβ25–35: ↑ BDNF-AS and ↓ BDNF BDNF-AS silencing restores MMP and reduces mitochondrial apoptosis/oxidative stress
36.	[80]	2023	Fundamental	Rodent (brain) Cell-based model	RT-qPCR	circRNA	Aβ1–42: ↑ circ_0004381 and ↓ miR-647; circ_0004381 sponges miR-647 to ↑ PSEN1; its knockdown reduces oxidative stress and mitochondrial dysfunction
37.	[81]	2023	Fundamental	Cell-based model	miRNA microarray; RT-qPCR	miRNA	AuNP treatment: ↑ miR-21-5p, miR-132, miR-107, miR-369, miR-181c and miR-212 ↓ miR-34a and miR-138; miR-21-5p/SOCS6 signaling contributes to protection against Aβ-induced mitochondrial injury
38.	[82]	2024	Fundamental	Rodent (brain) Cell-based model	RT-qPCR	miRNA	miR-375-3p overexpression enhances mitophagy; Reduces Aβ-induced mitochondrial dysfunction and apoptosis via PS1/mTOR signaling
39.	[83]	2023	Translational	Whole blood	Small RNA-Sequencing	miRNA	↓ miR-6501-5p and ↓ miR-143-3p in AD miR-6501-5p predicted to target ATP5E; miR-143-3p predicted to target NDUFB9, NDUFA9, NDUFS1 and COX4I1
40.	[84]	2008	Mixed	Human (brain) Cell-based model	RT-qPCR	miRNA	Braak stages 5–6 AD vs Braak stages 0–1 controls: Hippocampus: ↑ miR-423 Medial frontal gyrus: ↑ miR-423 Cerebellum: ↓ miR-98 miR-423 and miR-98 target IDH2
41.	[85]	2025	Fundamental	Rodent (brain) Cell-based model	RT-qPCR	miRNA	CeO₂-DRP/MA treatment resulted in ↓ relative miRNA-204 levels, increased MMP, and ↓ PINK1, Beclin-1 and LC3B
42.	[86]	2020	Fundamental	Rodent (brain) Cell-based model	RT-qPCR	miRNA	AD osteoblasts: miR-96-5p inhibitor increases Foxo1, decreases Lcn-2 Hippocampus of AD mice: miR-96-5p inhibitor reduced oxidative stress markers

ᵃStudy used an in silico approach instead of/in addition to performing experimental RNA quantification

This table summarizes the publication year, study type, experimental model, RNA processing method, ncRNA class studied as well as the main findings of each study selected for data extraction after screening. Studies using human tissue like CSF, serum, plasma, blood or human omics datasets were termed “translational,” while in vitro and in vivo studies were referred to as “fundamental.” Studies employing both approaches were termed “mixed studies.” The downregulation and upregulation of ncRNAs, other genes and proteins are represented using downward arrows (↓) and upward arrows (↑).The RNA quantification column indicates only experimental RNA expression assessment methods. N/A indicates that no experimental RNA quantification methods relevant to the study design were reported

*AD*, Alzheimer’s disease; *circRNA*, circular RNA; *CSF*, cerebrospinal fluid; *lncRNA*, long non-coding RNA; *miR*, microRNA; *N/A*, not applicable; *ncRNA*, non-coding RNA; *Poly(A) RNA-seq*, polyadenylated RNA sequencing; *RNA-seq*, RNA sequencing; *ROS*, reactive oxygen species; *RT-qPCR*, reverse transcription quantitative polymerase chain reaction; *total RNA-seq*, total RNA sequencing

*Gene and protein symbols*: *A2M-AS1*, alpha-2-macroglobulin antisense RNA 1; *BDNF-AS*, brain-derived neurotrophic factor antisense RNA; *Dicer1*, double-stranded RNA-specific endoribonuclease 1; *GABARAPL1*, GABA type A receptor-associated protein-like 1*; LOXL1-AS1*, lysyl oxidase-like 1 antisense RNA 1; *MDH2*, malate dehydrogenase 2; *MEG3*, maternally expressed gene 3; *MFN2*, mitofusin 2; *MGME1*, mitochondrial genome maintenance exonuclease 1; *MIR600HG*, miR-600 host gene; *Miro2*, mitochondrial Rho GTPase 2; *NDUFB10*, NADH:ubiquinone oxidoreductase subunit B10; *NDUFS1*, NADH:ubiquinone oxidoreductase core subunit S1; *NDUFS2*, NADH:ubiquinone oxidoreductase core subunit S2; *NDUFS3*, NADH:ubiquinone oxidoreductase core subunit S3; *NDUFS8*, NADH:ubiquinone oxidoreductase core subunit S8; *NDUFV1*, NADH:ubiquinone oxidoreductase core subunit V1; *NEAT1*, nuclear paraspeckle assembly transcript 1; *OPA1*, optic atrophy 1; *PDHA1*, pyruvate dehydrogenase E1 subunit alpha 1; *PINK1*, PTEN-induced putative kinase 1; *SDHB*, succinate dehydrogenase complex iron sulfur subunit B; *SNHG1*, small nucleolar RNA host gene 1; *TFEB*, transcription factor EB; *TOMM20*, translocase of the outer mitochondrial membrane 20; *UQCRFS1*, ubiquinol-cytochrome c reductase Rieske iron-sulfur polypeptide 1; *WT1-AS*, Wilms tumor 1 antisense RNA

The mitochondria-relevant results for each ncRNA class were organized into Tables 2, 3, and 4. Studies describing cross-class mechanisms were listed in the ncRNA table according to the primary mechanistic focus [45–47]. In all other multi-class ncRNA articles, findings were organized separately into the relevant tables [48, 49]. Tables 2 and 3 were divided into patient-based (“translational”) and model-based (“fundamental”) sub-sections in order to highlight clinically relevant evidence. The complete data extraction in Tables 2, 3, and 4 including sample type and RNA-quantification-methods are present in the Supplementary Material. Statistical data related to ncRNA expression in the form of *p*-values were reported in the Results column of each table, where available, and only the fold-change values explicitly reported in numerical format in the original studies were documented.

**Table 2:** MicroRNA dysregulation and mitochondrial dysfunction in AD: overview of translational and fundamental studies

MiRNA—translational studies		
Reference	Subjects or experimental model	Results
[51]	AD patients and controls Meta-analysis: - 36 transcriptomic datasets (7 for RNA-seq and 29 for microarray) - 45 differential miR expression studies	AD vs. controls: - ↓ miR-132-3p (logOR = − 5.08; 95% CI − 6.41 to − 3.74; FDR = 1.19 × 10⁻12) - ↓ miR-212-3p (logOR = − 6.37; 95% CI − 7.53 to − 5.21; FDR = 2.38 × 10⁻25) - miR-9-5p frequently reported as differentially expressed, but not among the statistically significant meta-analytic DEmiRNAs
[52]	Discovery datasets: - Religious orders study and the rush memory and aging project - miR cohorts: control (*n* = 180), MCI (*n* = 111), AD (*n* = 147), other (*n* = 12) - mRNA cohorts: control (*n* = 200), MCI (*n* = 165), AD (*n* = 251), other (*n* = 12) Validation dataset: - Alzheimer’s disease neuroimaging initiative - mRNA cohorts: control (*n* = 246), MCI (*n* = 383), AD (*n* = 114)	AD vs controls: - ↓ miR-129-5p (FDR < 0.01) - ↓ miR-132 (FDR < 0.01) ceRNA regulatory axes: - miR-132-3p–NEAT1–circ_0132052 - miR-129-5p–MALAT1–circ_0066962
[53]^e^	Training set: GSE5281 *n* = 87 controls *n* = 74 AD samples	AD vs. controls: - ↓ HIBCH and MGME1 (> twofold; *p* < 0.001) - miR-922 predicted and experimentally validated as a direct regulator of MGME1
	Validation set: GSE63061 *n* = 139 AD samples *n* = 249 controls	
[54]	GSE118553 GSE48350 GSE33000 *n* = 377 AD patients *n* = 223 controls	AD vs. controls: - miR-103 and miR-484 identified as predicted regulators of mitochondrial hub genes (UQCRFS1, NDUFS2, NDUFS3, NDUFS1, SDHB, MDH2, PDHA1, NDUFS8, NDUFB10, and NDUFV1) - miR-103 showed a reported interaction with NDUFV1; the role of miR-103/miR-484 in AD remains insufficiently characterized
[55]	*n* = 10 AD *n* = 12 controls *n* = 12 nondemented with Alzheimer’s neuropathology (NDAN)	AD vs controls and NDAN: - ↑ miR-485-5p vs controls (*p* = 0.007) - ↑ miR-485-5p vs NDAN (*p* = 0.003) - no significant difference between controls and NDAN (*p* = 0.0909) - ↓ PGC1α in AD; elevated miR-485-5p was proposed to contribute to PGC1α inhibition
[56]	NeuroPro database: 11 proteomic studies of human AD hippocampal tissue	AD hippocampus: - miR-106b-5p, miR-17-5p and miR-16-5p identified as regulators associated with altered proteins - associated target proteins enriched in ribosomal function, autophagy and immune-system pathways
[57]	*n* = 3 AD patients (79.3 ± 9.2 years) *n* = 3 controls (78.4 ± 5.7 years)	AD vs age-, sex- and PMI-matched controls: - ↑ miR-9, miR-34a, miR-146a and miR-155 (1.7- to 3.3-fold) - miR-9, miR-34a, miR-146a and miR-155 have recognition sites in the SIRT1 mRNA 3′UTR - their upregulation was proposed to contribute to ↓ SIRT1 expression in AD
[58]^e^	*n* = 36 controls *n* = 24 aMCI *n* = 36 AD	AD vs. controls (|FC|> 1.5; *p* < 0.05): - ↑ miR-1273g-3p - ↑ miR-8063 - ↓ miR-6798-3p - ↓ miR-106b-5p - ↓ miR-363-3p aMCI vs controls (|FC|> 1.5; *p* < 0.05): - ↑ miR-1273g-3p - ↑ miR-375
	*n* = 31 control *n* = 12 PSAD (presymptomatic AD) *n* = 20 PDAD (prodromal AD) *n* = 20 AD	AD vs. controls: - ↑ miR-1273g-3p (*p* < 0.05) AD vs. PSAD: - ↑ miR-1273g-3p (*p* < 0.01) PDAD vs. controls: - no significant difference in miR-1273g-3p
	*n* = 13 controls *n* = 10 PSAD *n* = 13 PDAD *n* = 14 AD	PSAD vs. controls: - ↑ miR-1273g-3p (*p* < 0.01) PDAD vs. controls: - ↑ miR-1273g-3p (*p* < 0.01) AD vs. controls: - ↑ miR-1273g-3p (*p* < 0.001) CSF miR-1273g-3p negatively correlated with CSF Aβ42 (*r* = −0.501; *p* < 0.001)
	Prefrontal cortex RNA-seq dataset: *n* = 383 AD *n* = 165 controls Post-mortem hippocampus: *n* = 8 AD *n* = 5 controls	AD vs. controls: - ↓ GLRX5, MTCH1 and TIMM13 mRNA in prefrontal cortex - ↓ TIMM13 protein in hippocampal CA1, CA2 and CA3
[59]^e^	*n* = 13 AD patients *n* = 10 controls	AD vs. age-matched controls: - ↑ miR-146a (3.6-fold; *p* ≤ 0.05) - miR-146a negatively correlated with VDAC1 protein levels (*ρ* = −0.80; *p* ≤ 0.01)
[60]^e^	*n* = 6 AD *n* = 6 controls	AD vs. controls: - ↓ miR-218-5p and miR-124-3p (*p* < 0.05) - ↑ RTN3 protein (*p* < 0.001)
[61]^e^	GSE173955 *n* = 8 AD (hippocampus) *n* = 10 controls	AD vs. controls: - ↓ OPA1 (*p* = 0.031) - ↓ MFN2, not significant (*p* = 0.058) AD vs. controls: - ↓ OPA1 (*p* = 0.029) - ↓ MFN2 (*p* = 0.00042)
	GSE183260 *n* = 19 AD (whole brain) *n* = 21 controls	
[62]	GSE5281: *n* = 87 AD *n* = 74 controls Validation dataset GSE132903: *n* = 97 AD *n* = 98 controls miRDB/HMDD databases: hub gene-targeting AD-associated miRNAs	GSE5281, AD vs. controls: - ↑ TFEB (logFC = 1.80; *P*adj = 1.75 × 10^−14^) - ↓ TOMM20 (logFC = −1.01; *P*adj = 1.77 × 10^−6^) - ↓ GABARAPL1 (logFC = −1.01; *P*adj = 1.57 × 10^−9^) Expression patterns of TFEB, TOMM20 and GABARAPL1 confirmed in GSE132903 (*p* < 0.001) miRDB/HMDD: - TFEB predicted targets: miR-29a-3p, miR-124-3p, miR-29b-3p, miR-29c-3p - TOMM20 predicted targets: let-7a-2-3p, miR-204-3p - GABARAPL1 predicted targets: miR-195-5p, miR-16-5p, miR-155-5p, miR-497-5p, miR-15b-5p, miR-143-3p, miR-133b
[64]^e^	*n* = 27 AD (65–95 years old) *n* = 15 controls (65–103 years old)	AD vs. controls: - ↑ miRNA PC-5P-12969 (*p* = 0.0008)
[83]	Statistical and machine learning analysis *N* = 7 AD (> 65 years old) *N* = 4 controls (> 65 years old)	AD compared to controls (peripheral blood): ↓miR-6501-5p (log2FC = − 1.34159, *p* = 0.003957) ↓miR-143-3p (log2FC = − 0.69404, *p* = 0.048802) Predicted mitochondrial target genes: - miR-6501-5p: predicted to target ATP5E - miR-143-3p: predicted to target NDUFB9, NDUFA9, NDUFS1 and COX4I1 Associated mitochondria-relevant terms as per Gene Ontology (GO) analysis: - miR-6501-5p**:** respiratory electron transport chain; generation of precursor metabolites and energy - miR-143-3p: respiratory electron transport chain; generation of precursor metabolites and energy; mitochondrial inner membrane; mitochondrial electron transport; NADH dehydrogenase activity; mitochondrial respiratory chain complex I
[84]^e^	Hippocampus, medial frontal gyrus, and cerebellum samples from age-matched *n* = 27 subjects: *n* = 2 Braak stage 0 (non-demented) *n* = 5 Braak stage 1 (non-demented) *n* = 5 Braak stage 3 (non-demented) *n* = 5 each Braak stages 4, 5, and 6 (AD)	Braak stages 5–6 AD vs. Braak stages 0–1 controls: Hippocampus: ↑ miR-423 (*p* < 0.05) Medial frontal gyrus: ↑ miR-423 (*p* < 0.05) Cerebellum: ↓ miR-98 (*p* < 0.05)
**MiRNA—fundamental studies**		
Reference	Subjects or experimental model	Results
[48]	SK-N-SH and CHP212 cells treated with Aβ	Aβ-treated vs. control cells: - ↓ miR-22-3p (*p* < 0.001) Mechanistic validation: - miR-22-3p mimic vs. miR-NC: ↓ PDE4A - miR-22-3p directly targets the PDE4A 3′UTR - miR-22-3p overexpression protected against Aβ-induced neuronal injury
[50]	APP/PS1 mice	APP/PS1 vs. WT littermates: - ↓ Dicer1 mRNA (4 months: *p* = 0.0335; 11 months: *p* = 0.0167) - ↓ Dicer1 protein (4 months: *p* = 0.0092; 11 months: *p* = 0.0002) - ↓ Nrf2 mRNA (4 months: *p* = 0.0304; 11 months: *p* = 0.0195) - ↓ Nrf2 protein (4 months: *p* = 0.0001; 11 months: *p* = 0.0038) - ↑ activated caspase-3 protein (4 months: *p* = 0.0098; 11 months: *p* = 0.0008)
	Primary murine hippocampal neurons	Dicer1 siRNA vs NC siRNA: - increased ROS (*p* < 0.0001) - decreased MMP (*p* = 0.0399) - ↑ activated caspase-3 protein (*p* = 0.020)
	Neuro-2a cells	Aβ42 vs. vehicle: - ↓ Dicer1 protein (*p* = 0.0004) - ↓ Nrf2 protein Nrf2 overexpression vs. vehicle plasmid: - ↑ Dicer1 mRNA (*p* = 0.0482) - ↑ Dicer1 protein (*p* = 0.024) Mechanistic validation: - Nrf2 directly regulates Dicer1 transcription through ARE sites in the Dicer1 promoter
[53]	293 T cells co-transfected with MGME1 3′UTR luciferase reporter constructs and miR-922 or miR-98-5p	MGME1-3′UTR-WT + miR-922 vs. MGME1-3′UTR-WT + miR-NC: - decreased luciferase activity by 42% (*p* = 0.003) - confirmed direct binding of miR-922 to MGME1 3′UTR MGME1-3′UTR + miR-98-5p: - no specific binding to MGME1
[58]	H4-APPswe and SH-SY5Y cells	H4-APPswe and SH-SY5Y + miR-1273g-3p mimic vs. mimic NC: - ↑ miR-1273g-3p (*p* < 0.01) - decreased MMP and mitochondrial respiration - increased mitochondrial fragmentation and oxidative stress - ↓ GLRX5, MTCH1 and TIMM13 Rescue experiments: - restored mitochondrial respiration and reversed BACE1/Aβ42-related changes
[59]	Primary cortical neurons from Sprague Dawley rats treated with Aβ	Aβ-treated vs. untreated primary cortical neurons: - ↑ intracellular miR-146a (*p* ≤ 0.05) - ↑ EV miR-146a (*p* ≤ 0.0001)
	Primary mixed glial cells from Sprague Dawley rats	Aβ-treated vs untreated primary mixed glial cells: - ↑ intracellular miR-146a (*p* ≤ 0.0001) - ↑ EV miR-146a (*p* ≤ 0.01) miR-146a overexpression vs. vector control in primary mixed glia: - ↑ intracellular miR-146a (> twofold) - ↑ EV miR-146a (53–116-fold) - decreased ATP-linked respiration, maximal respiration and spare respiratory capacity, glycolysis, glycolytic capacity and glycolytic reserve
[60]	Male 3 × Tg-AD mice subjected to social isolation Adult male C57BL/6 mice (wild-type) subjected to social isolation	Socially isolated vs. group-housed AD and WT mice: - ↓ miR-218-5p and miR-124-3p Socially isolated AD mice + miR-218-5p/miR-124-3p agomirs vs. scramble: - restored DG-CA3 synaptic plasticity and pattern separation Mechanistic validation: - miR-218-5p and miR-124-3p directly bind the Rtn3 3′UTR and posttranscriptionally suppress RTN3 protein - RTN3 upregulation/aggregation disrupted mitochondrial distribution in presynaptic boutons
[61]	3xTg-AD mice	3 × Tg-AD vs. WT mice: - ↑ miR-323-5p and miR-370-3p (> twofold, *p* < 0.001) - ↓ OPA1 and MFN2 protein
	Adult rat hippocampal neural stem/progenitor (HCN) cells - insulin withdrawal - miR-150-3p, miR-323-5p or miR-370-3p transfection	Insulin withdrawal vs. control: - ↑ miR-150-3p, miR-323-5p and miR-370-3p miRNA transfection vs control: - ↓ OPA1 - miR-150-3p additionally ↓ MFN2 - decreased mitochondrial respiration and ATP production - increased mitochondrial fission Mechanistic validation: - miR-150-3p directly binds the MFN2 3′UTR (30% reduction in reporter activity, *p* < 0.001) - OPA1 is a potential target of all three miRNAs
[63]	HCN cells	miR-351-5p mimic vs. miR-Con: - ↓ Miro2 mRNA by 71% (*p* < 0.0001) - ↓ Miro2 protein by 41% (*p* < 0.01) - increased mitochondrial fission, mitophagy, autophagy-dependent cell death - decreased MMP and ATP production Mechanistic validation: - miR-351-5p directly targets the Miro2 3′UTR (37% reduction in reporter activity; *p* < 0.001)
	AD model: 5xFAD mice, 3xTg-AD mice WT mice	3xTg-AD and 5xFAD vs. WT mice: - ↑ miR-351-5p - ↓ Miro2
[64]	APP transgenic mice (Tg2576), 6 months old (*n* = 5) Tau transgenic mice (P301L), 6 months old (*n* = 5) Aβ knock-in mice (hA-KI), 12 months old (*n* = 5) Wild-type C57BL/6 mice (*n* = 5)	vs respective WT controls: - ↑ miRNA PC-5P-12969 in APP mice (*p* = 0.0117) - ↑ miRNA PC-5P-12969 in Tau mice (*p* = 0.0110) - no significant change in 6-month hA-KI mice (*p* = 0.9606) - ↑ miRNA PC-5P-12969 in 12-month hA-KI mice (*p* = 0.0362)
	HT22 cells (untreated) HT22 cells + miRNA PC-5P-12969 vector HT22 cells + mAPP plasmid vector HT22 cells + miRNA PC-5P-12969 + mAPP vectors (pre-condition) HT22 cells + mAPP + miRNA PC-5P-12969 vectors (post-condition)	vs untreated HT22 cells: - ↑ miRNA PC-5P-12969 with mAPP (*p* < 0.0001) - ↑ miRNA PC-5P-12969 with miRNA PC-5P-12969 vector (*p* < 0.0001) - ↑ miRNA PC-5P-12969 in post-condition (*p* = 0.0001) - no significant change in pre-condition (*p* = 0.9693) Post-condition vs. mAPP: - ↓ GSK3α and APP - increased ATP production and maximal mitochondrial respiration - increased mitochondrial length - decreased mitochondrial fragmentation Mechanistic validation: - miRNA PC-5P-12969 directly binds the GSK3α 3′UTR (*p* < 0.0001)
[65]	*n* = 3 APP/PSEN1 double transgenic mice *n* = 3 wild-type (WT) mice	APP/PSEN1 vs. WT mice: - cortex: ↑ miR-7a (2.05-fold); ↓ miR-34a - hippocampus: ↑ miR-7a and miR-148a; ↓ miR-128a - ↑ Klf4 protein (*p* = 0.015 by immunofluorescence; *p* < 0.05 by Western blot)
	N9 microglial cells primed with lipopolysaccharide, followed by treatment with Aβ oligomers for 24 h	LPS-primed N9 + AβO + miR-7a mimic vs. LPS-primed N9 + AβO: - decreased ROS - ↓ iNOS and NLRP3
	SH-SY5Y cells treated with RAS-selective lethal 3 and vehicle (ferroptosis model)	RSL3 + miR-7a mimic vs. RSL3: - ↓ Klf4 - decreased ROS - restored normal long tubular mitochondrial morphology - increased MMP (complete recovery at 250 nM miR-7a) Mechanistic validation: -miR-7a directly binds the Klf4 3′UTR and suppresses Klf4
	SH-SY5Y cells	miR-7a mimic vs negative control: ↓ Klf4 miR-7a inhibitor treatment: ↑ Klf4
[66]	*n* = 42 APP/PS1 mice *n* = 6 wild type C57BL/6J mice	AD vs. WT: - ↑ miR-9-5p (*p* < 0.001) - ↓ SIRT1 - decreased ATP, MMP, and mitochondrial respiratory function AD + Rg1 vs. AD + Vehicle 1: - ↓ miR-9-5p (*p* < 0.001) - ↑ SIRT1 - improved mitochondrial function AD + Rg1 + miR-9-5p agomir vs. AD + Rg1 + agomir NC: - ↑ miR-9-5p (*p* < 0.05) - ↓ SIRT1 - increased mitochondrial dysfunction
	Mouse hippocampal neuron HT-22 treated with Aβ_1–42_ (20 µM)	Aβ1–42 vs. blank: - ↑ miR-9-5p (*p* < 0.001) - ↓ SIRT1 - ↓ PINK1, Parkin, LC3B II/I, and Beclin-1 Aβ1–42 + Rg1 vs. Aβ1–42 + Vehicle 1: - ↓ miR-9-5p (*p* < 0.001) - ↑ SIRT1 - ↑ PINK1, Parkin, LC3B II/I and Beclin-1 Aβ1–42 + Rg1 + miR-9-5p mimic vs Aβ1–42 + Rg1 + mimic NC: - ↑ miR-9-5p (*p* < 0.05) - ↓ SIRT1 - ↓ PINK1, Parkin, LC3B II/I and Beclin-1 - decreased ATP, MMP, and mitochondrial respiratory function
[67]	Primary human neuronal cultures from human induced pluripotent stem cells (hiPSCs) exposed to 1 µM Aβ−42o	hiPSC-NSC-EVs (containing miR-148a-3p, miR-21-5p and miR-103a-3p) + Aβ−42o vs Aβ−42o alone: - decreased mitochondrial superoxide - ↓ BAD and BAX; ↑ BCL-2 - increased MMP and preserved TOM20 + mitochondria - ↑ Beclin-1 and MAP1LC3B - normalized mitochondrial complex gene expression
[68]	Male Sprague–Dawley rats, induced via aggregated injection Aβ1–42 into the hippocampus	AD vs. normal rat hippocampus: - ↑ miR-140 (*p* < 0.05) - ↓ PINK1 miR-140 antagomir vs. NC-antagomir: - ↑ PINK1 - increased autophagy
	Primary hippocampal neurons from 1-day-old Sprague–Dawley rats treated with Aβ-derived diffusible ligands	AβDDLs-treated vs control neurons: - ↑ miR-140 (*p* < 0.05) - ↓ PINK1 - increased ROS and mitochondrial dysfunction; decreased autophagy miR-140 inhibitor vs. NC-inhibitor: - ↓ miR-140 (*p* < 0.05) - ↑ PINK1 - increased MMP and autophagy; decreased ROS
[69]	Mouse hippocampal neuron cell line HT22 treated with Aβ25–35	Aβ25–35-treated vs untreated HT22 cells: - ↓ miR-9-5p (*p* < 0.05) - ↑ GSK-3β Aβ25–35 + miR-9-5p mimic vs Aβ25–35 + NC mimic: - ↑ miR-9-5p (*p* < 0.01) - ↓ GSK-3β - decreased ROS/oxidative stress and apoptosis - ↓ cytochrome C, AIF, cleaved caspase-3 and BAX; ↑ BCL-2
[70]	SAMP8 mice aged 3-, 6- and 9- months (hippocampus)	SAMP8 vs age-matched SAMR1 hippocampus: - ↑ miR-195 at 3 and 6 months (*p* < 0.05); no significant change at 9 months - ↓ miR-15b at 3, 6 and 9 months (*p* < 0.05) - ↓ MFN2 mRNA and protein
	HT-22 hippocampal neuronal cells transfected with miR-195 mimics	miR-195 mimic vs scramble in HT-22 cells: - ↑ miR-195 (*p* < 0.05) - ↓ MFN2 protein - decreased MMP
[71]	Rat hippocampal neurons treated with Aβ1–42 Normal hippocampal neurons (control)	Aβ1–42-treated vs. control neurons: - ↑ miR-204 (*p* < 0.05) - ↓ TRPML1 - increased mitochondrial dysfunction/ROS and impaired mitochondrial autophagy Aβ1–42 + miR-204 inhibitor vs. Aβ1–42 + inhibitor negative-control: - ↑ TRPML1 - ↓ PINK1, Parkin, LC3II, and Beclin1 - increased MMP; decreased ROS and mitochondrial autophagy Aβ1–42 + CLN (STAT3 activation) vs. Aβ1–42 + vehicle: - ↓ miR-204 (*p* < 0.05) - ↑ TRPML1 - increased MMP and ATP; decreased ROS and mitochondrial autophagy
	APP/PS1 transgenic AD mouse model	APP/PS1 vs. normal hippocampus: - ↑ miR-204 (*p* < 0.05) - ↓ TRPML1 - decreased mitochondrial autophagy and increased mitochondrial ROS
[72]	SH-SWE cells (transfected with human APP695)	SH-SWE vs. SH-WT: - ↑ miR-124, miR-125b, and miR-146a (*p* < 0.05); ↑ miR-21 and miR-155 (*p* < 0.01) - ↑ exosomal miR-124 (*p* < 0.05) and miR-155 (*p* < 0.01) miR-124 mimic vs. mock in SH-SWE: - ↑ miR-124 (70-fold; *p* < 0.01) - ↓ exosomal miR-125b (*p* < 0.05), miR-146a and miR-155 (*p* < 0.01); miR-21 decreased, NS - increased mitochondrial activity/MitoTracker intensity miR-124 inhibitor vs. mock in SH-SWE: - ↓ miR-124 (≥ 80% decrease; *p* < 0.01) - ↑ miR-21 (*p* < 0.05) and miR-146a (*p* < 0.01); ↓ miR-125b (*p* < 0.05) - increased MFN2 protein; no significant change in DRP1
	iNEU-PSEN (iNeurons from iPSCs generated from a patient carrying the PSEN1ΔE9 mutation) and iNEU-wild type from a healthy female control	iNEU-PSEN vs. iNEU-WT: - ↑ miR-124, miR-125b, and miR-21 (*p* < 0.05) - ↓ miR-146a and miR-155 (*p* < 0.05) - ↑ exosomal miR-124 and miR-155 (*p* < 0.01) miR-124 mimic vs. mock in iNEU-PSEN: - ↑ miR-124 (~ 12-fold; *p* < 0.01) - ↓ miR-125b (*p* < 0.01) and miR-21 (*p* < 0.05) - ↓ exosomal miR-21 and miR-146a (*p* < 0.05) and miR-155 (*p* < 0.01); miR-125b decreased, NS miR-124 inhibitor vs. mock in iNEU-PSEN: - ↓ miR-124 (≥ 80% decrease; *p* < 0.01) - ↓ miR-125b (*p* < 0.05) and miR-21 (*p* < 0.01); ↑ miR-146a (*p* < 0.01)
[73]	APP Swedish SH-SY5Y cells (SWE)	SWE vs. wild-type SH-SY5Y (SH): - ↑ miR-124 in cells and secretome (cellular, exosomal, and EXO-free; *p* < 0.05)
	Human CHME3 microglial cells treated with IFNγ and co-cultured with SWE cells	IFNγ-microglia co-cultured with SWE vs IFNγ-microglia monoculture: - ↑ miR-124 in microglia (nearly 90-fold; *p* < 0.001) IFNγ-microglia co-cultured with miR-124-mimic SWE vs IFNγ-microglia co-cultured with mock SWE: - ↑ miR-124 in SWE cells (70-fold; *p* < 0.01) and secretome (exosomal and EXO-free; *p* < 0.05) - ↑ miR-124 and ↓ miR-146a in co-cultured microglia (*p* < 0.01) - ↓ iNOS and TNF-α expression in microglia - increased mitochondrial activity/MitoTracker Red intensity
[74]	N2a mouse neuroblastoma cells transfected with mutant APP cDNA	Mutant APP + miR-455-3p vs. mutant APP - increased mitochondrial biogenesis - decreased mitochondrial fragmentation - ↓ mitochondrial fission markers (DRP1 and FIS1) - ↑ mitochondrial fusion markers (OPA1, MFN1, and MFN2)
[81]	Human neural stem cells (hNSCs) (H9-derived) treated with Aβ1–42 ± gold nanoparticles (AuNPs)	Aβ + AuNPs vs. Aβ - ↑ miR-21-5p (FC = 4.78), miR-132, miR-107, miR-369, miR-181c, and miR-212 - ↓ miR-34a and miR-138 - ↑ DRP1, NRF1, and TFAM mRNA - increased MMP and decreased mitochondrial ROS - decreased apoptosis Aβ + AuNPs + miR-21-5p inhibitor vs Aβ + AuNPs + NC - ↓ miR-21-5p (*p* < 0.01) - ↓ DRP1, NRF1, and TFAM mRNA - decreased cell viability - decreased MMP, increased mitochondrial ROS, and increased apoptosis miR-21-5p mimic vs. mimic-NC - ↑ miR-21-5p (*p* < 0.01) - ↓ SOCS6 protein - increased cell viability and decreased apoptosis
[82]	SH-SY5Y cells treated with Aβ25–35	SH + Aβ25–35 + miR-375-3p vs. SH + NC + Aβ25–35 - decreased Aβ25–35-induced apoptosis - decreased intracellular ROS - increased MMP/JC-1 polymer:monomer fluorescence ratio
	APP/PS1 transgenic mice	AD + miR-375-3p vs. AD control - ↑ LC3B, Beclin-1, MFN2, and PINK1 mRNAs
[85]	*n* = 10 3 × Tg-AD mice receiving PBS injections in the tail veil *n* = 10 3 × Tg-AD mice receiving CeO_2_-DRP/MA 1 mmol/L (CeO_2_ nanoparticles covalently linked with mitochondrial aptamers (MA) and DNA-RNA hybrid chains (DRP) to detect and silence mitochondrial miRNA-204) injections in the tail vein *n* = 10 C57BL/6 mice	3 × Tg-AD treated with CeO_2_-DRP/MA vs. PBS-treated 3 × Tg-AD mice: - Decreased escape latency (*p* < 0.05) - Increased swimming speed (Morris water maze) - Decreased depression-related and anxiety-related behaviors Hippocampal tissue of CeO_2_-DRP/MA treated vs. PBS-treated 3 × Tg-AD mice: - ↓ PINK1, Beclin1, and LC3B mRNA/protein - ↑TRPML1 and p-STAT3 mRNA/protein
	SY5Y + A*β* cells	SY5Y + Aβ + CeO2-DRP/MA (1 mmol/L) vs. untreated SY5Y + Aβ cells: - increased cell viability from ~ 50% at 0 mmol/L to 98% at 1 mmol/L after 24 h - increased MMP - ↓ mitochondrial miRNA-204 (*p* < 0.01) - ↓PINK1, Beclin-1, and LC3B mRNA - ↑TRPML1 and STAT3 mRNA
	N2aSW cells	CeO2-DRP/MA-treated vs. untreated N2aSW cells: - ↓PINK1, Beclin-1, LC3B mRNA/protein - ↑TRPML1, STAT3 mRNA/protein
[84]	SK-N-AS (human neuroblastoma cells)	Pre-miR-423 vs. negative control-transfected cells: - ↓ IDH2 mRNA (59.33% and 60.19% of control in two independent experiments; *p* < 0.05)
	HepG2 (human hepatocellular carcinoma)	Pre-miR-98 vs. negative control-transfected cells: - ↓ IDH2 mRNA (70.03% and 43.98% of control in two independent experiments; *p* < 0.05)
[86]	Kunming mice: *n* = 6 normal *n* = 6 sham *n* = 66 successfully modeled AD mice after bilateral hippocampal Aβ1–40 injection, divided into 11 groups (*n* = 6/group)	AD-mice derived osteoblasts: - AD + naringin vs. AD + PBS: ↓ miR-96-5p (*p* < 0.05) - AD + miR-96-5p inhibitor vs. AD + inhibitor-NC: ↓ miR-96-5p (*p* < 0.05), ↑ Foxo1, ↓Lcn-2 - AD + miR-96-5p mimic vs. AD + miR-96-5p mimic-NC: ↓ Foxo1, ↑Lcn-2
		Hippocampus of AD mice: - AD + miR-96-5p inhibitor vs. AD + inhibitor-NC: - Reduced OH- and MDA - Increased SOD, CAT and GSH-Px - AD + sh-Lcn2 vs. AD + sh-NC: Reduced mitochondrial structural damage (qualitative assessment) - AD + oe-Foxo1 vs. oe-NC: - Reduced mitochondrial structural damage (qualitative assessment) - Reduced OH- and MDA - Increased SOD, CAT and GSH-Px - ↓ Lcn2

^e^Studies marked with a superscript “e” included additional experimental validation directly assessing the reported ncRNA or its proposed regulatory interaction which was accordingly included in the “Fundamental” section of the table

This table synthesizes the experimental model and mitochondrially relevant results of microRNA-focused studies. Studies using human tissue like CSF, serum, plasma, blood or human omics datasets were termed “translational,” while in vitro and in vivo studies were referred to as “fundamental.” The first part of the table summarizes translational data while the second part of the table summarizes fundamental experimental findings. For those studies which featured both translational and fundamental results, the findings were placed in the respective table segment. The downregulation and upregulation of ncRNAs, other genes and proteins are represented using downward arrows (↓) and upward arrows (↑)

*3xTg-AD mice*, triple-transgenic Alzheimer’s disease model mice; *AD*, Alzheimer’s disease; *aMCI*, Aβ-positive amnestic mild cognitive impairment; *APP*, β-amyloid precursor protein; *ATP*, adenosine triphosphate; *AuNPs*, gold nanoparticles; *Aβ*, amyloid-beta; *ceRNA*, competing endogenous RNA; *CSF*, cerebrospinal fluid; *DEmiRNA/DEmiRNAs*, differentially expressed microRNA(s); *EV*, extracellular vesicles; *HCN*, adult hippocampal neural progenitor cells; *hiPSC*, human induced pluripotent stem cell; *hNSC*, human neural stem cell; *iPSC*, induced pluripotent stem cell; *lncRNA*, long non-coding RNA; *MAP*, Rush Memory and Aging Project; *mAPP*, mutant APP; *MCI*, mild cognitive impairment; *miR/miRNA*, microRNA; *MMP*, mitochondrial membrane potential; *mRNA*, messenger RNA; *N/A*, not applicable; *ncRNA*, non-coding RNA; *NDAN*, nondemented patients with Alzheimer’s neuropathology; *PDAD*, prodromal Alzheimer’s disease; *PSAD*, presymptomatic Alzheimer’s disease; *RNA-seq*, RNA sequencing; *ROS*, reactive oxygen species; *RT-qPCR*, reverse transcription quantitative polymerase chain reaction; *SAMP8*, senescence-accelerated mouse prone 8; *WT*, wild-type; *PBS*, phosphate-buffered saline; *CeO₂*, cerium dioxide; *DRP*, DNA–RNA hybrid chains; *MA*, mitochondrial aptamers; *DNA*, deoxyribonucleic acid; *RNA*, ribonucleic acid; *FAM*, fluorescein amidite; *IVIS*, in vivo imaging system; *NC*, negative control; *MDA*, malondialdehyde; *SOD*, superoxide dismutase; *CAT*, catalase; *GSH-Px*, glutathione peroxidase; *shRNA*, short hairpin RNA; *oe*, overexpression, *RSL3*, RAS-selective lethal 3; *log2FC*, log2 fold change; *GO*, Gene Ontology

*Gene and protein symbols*: *AIF*, apoptosis-inducing factor; *BAD*, Bcl-2-associated death promoter; *BAX*, BCL2-associated X protein; *BCL-2*, B-cell lymphoma 2; *Dicer1*, double-stranded RNA-specific endoribonuclease 1; *DRP1*, dynamin-related protein 1; *FIS1*, mitochondrial fission protein 1; *GABARAPL1*, GABA type A receptor-associated protein-like 1; *GSK-3β*, glycogen synthase kinase-3 beta; *GSK3*, glycogen synthase kinase 3; *HIBCH*, 3-hydroxyisobutyryl-CoA hydrolase; *iNOS*, inducible nitric oxide synthase; *Klf4*, Krüppel-like factor 4; *LC3B*, microtubule-associated protein 1 light chain 3B; *MAP1-LC3B*, microtubule-associated proteins 1A/1B light chain 3B; *MDH2*, malate dehydrogenase 2; *MFN1*, mitofusin 1; *MFN2*, mitofusin 2; *MGME1*, mitochondrial genome maintenance exonuclease 1; *Miro2*, mitochondrial Rho GTPase 2; *NDUFB10*, NADH:ubiquinone oxidoreductase subunit B10; *NDUFS1*, NADH:ubiquinone oxidoreductase core subunit S1; *NDUFS2*, NADH:ubiquinone oxidoreductase core subunit S2; *NDUFS3*, NADH:ubiquinone oxidoreductase core subunit S3; *NDUFS8*, NADH:ubiquinone oxidoreductase core subunit S8; *NDUFV1*, NADH:ubiquinone oxidoreductase core subunit V1; *NLRP3*, NLR family pyrin domain-containing protein 3; *NRF1*, nuclear respiratory factor 1; *NRF2*, nuclear factor erythroid 2-related factor 2; *OPA1*, optic atrophy 1 gene; *Parkin*, E3 ubiquitin-protein ligase parkin; *PDE4A*, phosphodiesterase 4 A; *PDHA1*, pyruvate dehydrogenase E1 subunit alpha 1; *PGC-1α*, peroxisome proliferator-activated receptor-gamma coactivator 1-alpha; *PINK1*, PTEN-induced putative protein kinase 1; *PSEN1*, presenilin 1; *RTN3*, reticulon 3 gene; *SDHB*, succinate dehydrogenase complex iron sulfur subunit B; *SIRT1*, sirtuin 1; *SOCS6*, suppressor of cytokine signalling 6; *STAT3*, signal transducer and activator of transcription 3; *TFEB*, transcription factor EB; *TIMM13*, translocase of the inner mitochondrial membrane 13; *TNF-α*, tumor necrosis factor alpha; *TOMM20*, translocase of the outer mitochondrial membrane 20; *TRPML1*, transient receptor potential mucolipin-1; *UQCRFS1*, ubiquinol-cytochrome c reductase Rieske iron-sulfur polypeptide 1; *UTR*, untranslated region; *VDAC1*, voltage-dependent anion channel 1, *IDH2*, isocitrate dehydrogenase 2, Beclin1, beclin 1; *FOXO1*, forkhead box O1; *LCN2*, lipocalin 2, *ATP5E*, ATP synthase F1 subunit epsilon; *NDUFB9*, NADH:ubiquinone oxidoreductase subunit B9; *NDUFA9*, NADH:ubiquinone oxidoreductase subunit A9; *COX4I1*, cytochrome c oxidase subunit 4I1

**Table 3:** Long non-coding RNA dysregulation and mitochondrial dysfunction in AD: overview of translational and fundamental studies

LncRNA—translational studies		
Reference	Subjects or experimental model	Results
[46]^e^	GEO GSE4757 (*n* = 10 controls, *n* = 10 AD human cortices) GEO GSE16759 (*n* = 4 controls, *n* = 4 AD human samples—parietal lobe cortex)	GSE4757, AD vs. controls: - ↓ WT1-AS (*p* = 0.0082) - ↓ SIX4 (*p* = 0.0055) GSE16759, AD vs. controls: - ↑ miR-375 (*p* = 0.0438)
[75]^e^	*n* = 8 controls (5 female, 3 male) *n* = 8 AD patients (4 female, 4 male, Braak stages IV–VI)	AD vs age- and sex-matched controls: - ↓ lncMtDloop in PFC (*p* = 0.0057) - ↓ lncMtDloop in hippocampal CA1 (*p* = 0.0076) - ↓ lncMtDloop in dentate gyrus, trend only (*p* = 0.063) - ↓ lncMtDloop by RT-qPCR in PFC (*p* = 0.0031) and hippocampus (*p* = 0.0027)
[76]^e^	*n* = 16 probable AD patients (7 males and 9 females, 70.3 ± 8.8 years) *n* = 18 healthy individuals (8 males and 10 females, 68.3 ± 9.6 years)	AD patients vs. healthy controls: - ↑ lncRNA-ATB in CSF (*p* < 0.001) - ↑ lncRNA-ATB in serum (*p* < 0.05)
**LncRNA—fundamental studies**		
Reference	Subjects or experimental model	Results
[45]	SH-SY5Y cells treated with Aβ25–35 human primary neurons treated with Aβ25–35	Aβ25–35-treated vs. untreated SH-SY5Y and human primary neurons: - ↑ SNHG1 (20 μM: *p* < 0.001) - decreased MMP Aβ25–35 + si-SNHG1 vs. Aβ25–35 + si-NC: - increased MMP and decreased apoptosis Mechanistic validation: - SNHG1 knockdown ↑ miR-137 (*p* < 0.01) - SNHG1 sponges miR-137; miR-137 directly targets KREMEN1 - miR-137 inhibition or KREMEN1 overexpression partially reversed the protective effects of SNHG1 knockdown
[46]	SH-SY5Y cells treated with Aβ_25–35_	Aβ25–35-treated vs. untreated SH-SY5Y cells: - ↑ miR-375 (p < 0.05) - ↓ WT1-AS and SIX4 (*p* < 0.05) Aβ25–35 + miR-375 mimic vs. Aβ25–35 + mimic-NC: - ↓ SIX4 mRNA and protein (*p* < 0.05) - decreased MMP and ATP production - increased oxidative stress and apoptosis Mechanistic validation: - miR-375 directly binds SIX4 mRNA - WT1 transcriptionally promotes miR-375 expression
	Male BALB/c mice, Aβ25–35-induced AD model *n* = 10/group	Aβ25–35 AD-model vs. sham mice: - ↑ miR-375 (*p* < 0.05) - ↓ WT1-AS and SIX4; ↑ WT1 AD + WT1-AS overexpression vs. AD + oe-NC: - ↓ miR-375 (*p* < 0.05) - ↑ SIX4 (*p* < 0.05) - decreased oxidative stress and neuronal apoptosis
[47]	PC12 cells treated with Aβ_25–35_	Aβ25–35-treated vs. untreated PC12 cells: - ↑ H19 and ↓ miR-129 (*p* < 0.05) - ↑ HMGB1 (*p* < 0.05) Aβ25–35 + si-H19 vs. Aβ25–35 + si-NC: - ↑ miR-129 and ↓ HMGB1 (*p* < 0.05) - decreased ROS and increased MMP (*p* < 0.05) Mechanistic validation: - H19 directly binds/sponges miR-129 - miR-129 directly targets the HMGB1 3′UTR
	BALB/c male mice injected with Aβ25–35 (intraventricular) *n* = 10/group	Aβ25–35 AD-model vs. sham mice: - ↑ H19 and ↓ miR-129 - ↑ HMGB1
[49]	hiPSC-derived glutamatergic forebrain neurons from PSEN1-mutant patients (L150P, A79V) and isogenic controls (fibroblast-derived)	A79V and L150P PSEN1-mutant neurons vs. respective gene-corrected isogenic controls: - ↓ MEG3 (A79V: L2FC = −5.88, *P*adj = 1.47 × 10⁻^2^⁹; L150P: L2FC = −6.31, *P*adj = 2.75 × 10⁻⁹^4^) - ↓ LOXL1-AS1 - ↓ A2M-AS1
[75]	WT and 3xTg-AD mouse-derived primary hippocampal neuron cultures	3xTg-AD vs. WT primary hippocampal neurons: - ↓ lncMtDloop (RT-qPCR: *p* = 0.0075) 3xTg-AD + LV-lncMtDloop vs. 3xTg-AD + LV-control: - ↑ mtRNA (*p* = 0.0363) - ↑ mitochondrial genome-encoded OXPHOS genes/proteins - ↓ Drp1 protein (*p* = 0.0352) - ↑ Opa1 protein (*p* = 0.0296) - increased basal and reserve mitochondrial respiration
	3xTg-AD mice Wild-type mice	12-month-old 3xTg-AD vs. age-matched WT mice: - ↓ lncMtDloop in PFC and hippocampus (RT-qPCR: *p* = 0.0059 and *p* = 0.0082, respectively) 3xTg-AD + AAV-lncMtDloop vs. 3xTg-AD + AAV-control: - restored mitochondrial ultrastructure and morphology in PFC and hippocampus - ↑ PINK1, Parkin and LC3B Mechanistic validation: - lncMtDloop directly interacts with TFAM and promotes its recruitment to mtDNA promoters, increasing mitochondrial transcription
[76]	Neuronal PC12 cells treated with Aβ _25–35_	Aβ25–35-treated vs. untreated PC12 cells: - ↑ lncRNA-ATB (*p* < 0.001) - increased ROS - decreased MMP Aβ25–35 + sh-ATB vs. Aβ25–35 + sh-NC: - ↓ lncRNA-ATB (*p* < 0.01) - decreased ROS - increased MMP sh-ATB vs. sh-NC: - ↑ miR-200 (*p* < 0.01) Aβ25–35 + sh-ATB + miR-200 inhibitor vs Aβ25–35 + sh-ATB + inhibitor NC: - increased ROS - decreased MMP miR-200 mimic vs. mimic NC: - ↓ ZNF217
[77]	APP/PS1 mice 5xFAD mice	APP/PS1 and 5xFAD mice > 6 months vs. age-matched WT: - ↑ MIR600HG (*p* < 0.05; age-dependent) APP/PS1 + AAV-shMIR600HG vs. untreated APP/PS1: - increased cytochrome c oxidase activity
	SH-SY5Y cells	SH-SY5Y + MIR600HG overexpression vs control: - ↑ PINK1–NEDD4L interaction - ↑ PINK1 ubiquitination - ↓ PINK1 - ↓ autophagy markers (LC3, OPTN and p62) SH-SY5Y + shMIR600HG vs. control: - ↑ PINK1 - ↑ autophagy markers (LC3, OPTN and p62)
[78]	APP/PS1 transgenic mice	APP/PS1 vs. WT: - ↑ lncRNA NEAT1 in older APP/PS1 mice (*p* < 0.01) APP/PS1 + AAV-NEAT1 vs. untreated APP/PS1: - decreased cytochrome c oxidase activity APP/PS1 + AAV-shNEAT1 vs. untreated APP/PS1: - increased/restored cytochrome c oxidase activity and ATP - decreased Aβ
	SH-SY5Y cells	NEAT1 overexpression vs. vector: - ↑ PINK1 ubiquitination and degradation - decreased PINK1-dependent autophagy NEAT1 knockdown vs. vector: - ↓ PINK1 ubiquitination - ↑ PINK1 and PINK1-dependent autophagy
	N2A-APPsw cells	shNEAT1 vs. control: - decreased Aβ42 (*p* < 0.01) shNEAT1 + siPINK1/siOPTN/siLC3 vs shNEAT1: - reversed the reduction in Aβ42
[79]	PC12 cells treated with Aβ_25–35_	Aβ25–35 vs. untreated PC12 cells: - ↑ lncRNA BDNF-AS (*p* < 0.05) - ↓ BDNF Aβ25–35 + si-BDNF-AS vs. Aβ25–35 + si-NC: - ↓ lncRNA BDNF-AS (*p* < 0.05) - ↑ BDNF and BCL-2; ↓ BAX and cleaved caspase-3 - increased MMP - decreased cytochrome c release and apoptosis - decreased ROS and MDA; increased SOD and CAT activity

^e^Studies marked with a superscript “e” included additional experimental validation directly assessing the reported ncRNA or its proposed regulatory interaction which was accordingly included in the “Fundamental” section of the table

This table synthesizes the experimental model and mitochondrially relevant results of lncRNA-focused studies. Studies using human tissue like CSF, serum, plasma, blood, or human omics datasets were termed “translational,” while in vitro and in vivo studies were referred to as “fundamental.” The downregulation and upregulation of ncRNAs, other genes and proteins are represented using downward arrows (↓) and upward arrows (↑). The first part of the table summarizes translational data while the second part of the table summarizes fundamental experimental findings. For those studies which featured both translational and fundamental results, the findings were placed in the respective table segment

*3xTg-AD mice*, Triple-transgenic Alzheimer’s disease model mice; *AD*, Alzheimer’s disease; *APP*, β-amyloid precursor protein; *ATP*, adenosine triphosphate; *AuNPs*, gold nanoparticles; *Aβ*, amyloid-beta; *CAT*, catalase; *CSF*, cerebrospinal fluid; *DG*, dentate gyrus; *GEO*, Gene Expression Omnibus; *hiPSC*, human induced pluripotent stem cell; *LC3*, microtubule-associated proteins 1A/1B light chain 3; *lncMtDloop*, long non-coding mitochondrial displacement loop RNA; *lncRNA*, long non-coding RNA; *lncRNA BDNF-AS*, long non-coding RNA brain-derived neurotrophic factor antisense; *lncRNA NEAT1*, lncRNA nuclear paraspeckle assembly transcript 1; *lncRNA WT1-AS*, Wilms tumor 1 antisense RNA; *MDA*, malondialdehyde; *miRNA*, microRNA; *MMP*, mitochondrial membrane potential; *mtRNA*, mitochondrial RNA; *N/A*, not applicable; *OSI*, oxidative stress index; *OXPHOS*, oxidative phosphorylation; *PFC*, prefrontal cortex; *Poly(A) RNA-seq*, polyadenylated RNA sequencing; *PSEN1*, presenilin 1; *RT-qPCR*, reverse transcription quantitative polymerase chain reaction; *SOD*, superoxide dismutase; *total RNA-seq*, total RNA sequencing

*Gene and protein symbols*: *A2M-AS1*, alpha-2-macroglobulin antisense RNA 1; *BAX*, BCL2-associated X protein; *BCL-2*, B-cell lymphoma 2; *BDNF-AS*, BDNF antisense RNA; *DRP1*, dynamin-related protein 1; *HMGB1*, high mobility group box 1; *LC3B*, microtubule-associated protein 1 light chain 3B; *LOXL1-AS1*, lysyl oxidase-like 1 antisense RNA 1; *MIR600HG*, miR-600 host gene; *NEDD4L*, neural precursor cell expressed, developmentally down-regulated 4-like, E3 ubiquitin protein ligase; *OPA1*, optic atrophy 1 gene; *OPTN*, optineurin; *p62*, sequestosome-1 (SQSTM1); *Parkin*, E3 ubiquitin-protein ligase parkin; *PINK1*, PTEN-induced putative protein kinase 1; *SIX4*, SIX homeobox 4; *WT1*, Wilms Tumor 1 gene; *WT1-AS*, Wilms tumor 1 antisense RNA; *ZNF217*, zinc finger protein 217

**Table 4:** Circular RNA dysregulation and mitochondrial dysfunction in AD: overview of translational and experimental studies

Reference	Subjects or experimental model	Results
[48]	SK-N-SH and CHP212 cells treated with 20 μM Aβ exposure for 24 h	Aβ-treated vs. control cells: - ↑ circ_0049472 (*p* < 0.001) - ↓ miR-22-3p (*p* < 0.001) Aβ + si-circ_0049472 vs. Aβ + si-NC: - ↓ circ_0049472 (*p* < 0.001) - ↑ miR-22-3p (*p* < 0.001) - ↑ BCL-2; ↓ BAX and cleaved-caspase-3 - increased/restored MMP
[49]	hiPSC-derived glutamatergic forebrain neurons from PSEN1-mutant patients (L150P, A79V) and isogenic controls (fibroblast-derived)	L150P vs. isogenic control: - ↑ CDR1as/circ_0001946 (L2FC = 2.38; *P*adj = 2.04 × 10^−10^) - ↓ UBE2A A79V vs. isogenic control: - circ_0001946: no significant change (L2FC = 0.67; *P*adj = 0.37) - ↓ UBE2A
[80]	Primary mouse hippocampal neurons treated with Aβ1–42	miR-647 inhibitor + sh-circ_0004381 vs. sh-circ_0004381: - ↓ miR-647 (*p* < 0.05) - increased ROS and cytoplasmic cytochrome c; decreased SOD - attenuated the increases in ATP and MMP - ↑ BAX/BCL-2 ratio
	Microglial BV2 cells treated with lipopolysaccharide	LPS + sh-circ_0004381 vs. LPS + sh-NC: - ↓ iNOS and COX-2 - decreased M1 and increased M2 polarization - decreased IL-1β, IL-6 and TNF-α
	Male APPswe/PS1dE9 transgenic mice	APPswe/PS1dE9 vs. non-transgenic controls: - ↑ circ_0004381 and ↓ miR-647 (*p* < 0.05) APPswe/PS1dE9 + sh-circ_0004381 vs. untreated APPswe/PS1dE9: - ↓ circ_0004381 and ↑ miR-647 (*p* < 0.05) - decreased ROS

This table synthesizes the tissue sample, experimental model, RNA quantification method and mitochondrially-relevant results of 3 circRNA-focused studies. No eligible circRNA studies using patient-derived data were identified after the screening process. All circRNA evidence was derived from experimental in vitro and in vivo studies, which we refer to as “fundamental studies.” The downregulation and upregulation of ncRNAs, other genes and proteins are represented using downward arrows (↓) and upward arrows (↑)

*AD*, Alzheimer’s disease; *APP*, β-amyloid precursor protein; *ATP*, adenosine triphosphate; *Aβ*, amyloid-beta; *circRNA*, circular RNA; *hiPSC*, human induced pluripotent stem cell; *MMP*, mitochondrial membrane potential; *N/A*, not applicable; *ncRNA*, non-coding RNA; *Poly(A) RNA-seq*, polyadenylated RNA sequencing; *RNA-seq*, RNA sequencing; *ROS*, reactive oxygen species; *RT-qPCR*, reverse transcription quantitative polymerase chain reaction; *SOD*, superoxide dismutase; *total RNA-seq*, total RNA sequencing

*Gene and protein symbols*: *BAX*, BCL2-associated X protein; *BCL-2*, B-cell lymphoma 2; *COX-2*, cyclooxygenase-2; *iNOS*, inducible nitric oxide synthase; *PSEN1*, presenilin 1; *UBE2A*, ubiquitin-conjugating enzyme E2A

The findings presented mechanistic evidence of variable strength and are discussed accordingly. Some studies performed direct mitochondrial assessments such as measures of ATP production, mitochondrial morphology, mitophagy, and mitochondrial dynamics while other studies reported indirect evidence such as that derived from bioinformatics analyses or oxidative stress and apoptosis assessments.

### Mitophagy and ncRNAs in AD

Mitophagy is the selective autophagy of dysfunctional mitochondria. This process is dependent on the PTEN-induced putative protein kinase 1 (PINK1) and the E3 ubiquitin ligase Parkin. Normally, PINK1 is imported to the inner mitochondrial membrane, but mitochondrial membrane depolarization leads to PINK1 accumulation on the outer membrane. This causes the recruitment of cytosolic Parkin, which ubiquitinates outer membrane proteins and leads to autophagic receptor accumulation [87]. Mitophagy involves various selective autophagy adaptors such as p62, optineurin (OPTN) and nuclear dot protein 52 (NDP52), which are recruited to dysfunctional mitochondria under the regulation Parkin/PINK1. The adaptors contain a ubiquitin-binding domain and a LC3-interacting region [88]. The LC3 proteins are part of the Atg8 family of proteins which conjugate to the autophagosomal membranes, which are essential for autophagosome-lysosome fusion [89].

Two experimental studies have demonstrated the involvement of miRNAs in regulating PINK1 in AD. Firstly, miR-351-5p suppressed mitochondrial Rho GTPase 2 (Miro2) in adult hippocampal neural progenitor cells, thus disrupting mitochondrial transport, inducing mitochondrial fission and stimulating mitophagy through the PINK1/Parkin pathway. In AD mice hippocampi, miR-351-5p was upregulated while Miro2 was downregulated. While miR-351-5p itself was not quantified in the human hippocampus, Miro2 was downregulated in AD patients [63]. Hence, therapeutic avenues to restore spatial mitochondrial distribution in neurons and maintain synaptic integrity may involve modulating miR-351-5p.

Secondly, miR-140 was shown to downregulate PINK1. MiR-140 overexpression was detected in both AD mice hippocampal tissues and Aβ oligomer-treated primary hippocampal neurons, which was associated with impaired autophagy. Functional inhibition of miR-140 in both models preserved mitochondrial function via the upregulation of PINK [68]. Other AD studies linked miR-140-5p upregulation in the human brain to ADAM10 downregulation and increased amyloidogenic processing of APP [90, 91]. miR-140 is also involved in mitochondrial dynamics and mitophagy in other cell types [92–94], encouraging further investigation.

PINK1 is also modulated by a series of long non-coding RNAs. Firstly, lncRNA NEAT1 upregulation was observed in the hippocampus of AD mice, alongside increased PINK1 ubiquitination and reduced autophagy markers (p62, OPTN, LC3). Its knockdown reversed these effects, confirming its inhibitory role on PINK1-dependent mitophagy [78]. The disruptive effect of NEAT1 on mitochondrial homeostasis observed by Huang et al. (2020) is in agreement with other studies. Elsewhere, NEAT1 overexpression was shown to disrupt mitochondrial biogenesis by interacting with peroxisome proliferator-activated receptor-gamma coactivator 1-alpha (PGC-1α) and inhibiting B-cell lymphoma 2 protein (BCL-2) and sirtuin 1 (SIRT1) [95]. NEAT1 knockdown reduces neuronal apoptosis rates in AD [96]. Interestingly, one study using PD mice found that NEAT1 prevented PINK1 degradation [97]. Nonetheless, the evidence suggests that NEAT1 regulates mitophagy and other mitochondrial processes in AD.

Secondly, Liu et al. (2022) found that lncRNA MIR600HG was upregulated in AD mice. MIR600HG upregulates E3 ligase NEDD4L (E3 ubiquitin protein ligase*,* neural precursor cell expressed, developmentally down-regulated 4-like*),* which decreases *PINK*1 activity via ubiquitination, consequently impairing mitophagic activation [77]. NEDD4L has been shown to suppress autophagy and negatively regulate mitochondrial energy metabolism [98, 99]. The findings of Liu et al. [77] regarding the NEDD4L/PINK1 axis are corroborated by a recent endothelial injury study [100].

Finally, lncRNA BACE1-AS knockdown via lentiviral modulation resulted in a significant increase in PINK1 and LC3-I/II expression in SH-SY5Y cells [101]. LncRNA BACE1-AS suppression increases cognitive performance in AD mice [102] and promotes autophagy [103], though further in vitro studies better replicating the hallmarks of AD pathophysiology are warranted.

In summary, PINK1 levels are decreased by miR-140 [68], lncRNA NEAT1 [78] and lncRNA MIR600HG [77] via various mechanisms in the preclinical AD studies identified in this review. Dysregulated levels of these ncRNAs have been reported in patients. MiR-140-5p is upregulated in post-mortem AD brains [90] while plasma miR-140-3p levels are predictive for mild cognitive impairment [104] and are elevated in stroke [105]. LncRNA BACE1-AS is a biomarker candidate and is upregulated in the plasma [106, 107] and serum of AD patients [108]. NEAT1 is upregulated in the plasma of AD [109] and PD patients [110]. MIR600HG is dysregulated in autism spectrum disorder [111], but additional studies are necessary to identify MIR600HG dysregulation patterns in AD. Finally, miR-351-5p, which stimulates PINK1/Parkin-dependent mitophagy, is upregulated in AD mouse hippocampi [63] but further studies are needed to confirm its dysregulation in patients.

### NcRNAs and Mitochondrially Mediated Cell Death in AD

Multiple cell death pathways are dysregulated in AD, from the autophagy-lysosome pathway [112] to high neuronal apoptosis rates [113] and ferroptosis [114]. In this section, we discuss the ncRNAs which help regulate autophagy, apoptosis and ferroptosis in AD, highlighting how they affect mitochondrial activity in each case.

Caspases are key agents in apoptosis. They can be activated through death receptors such as those in the tumor necrosis factor receptor superfamily or via the intrinsic, mitochondria-dependent pathway [115]. The mitochondrial pathway involves mitochondrial outer membrane permeabilization (MOMP), which enables cytochrome c to leave the intermembrane space and enter the cytosol, where it activates executioner caspases-3 and −7. MOMP is regulated by pro-apoptotic effector proteins BCL-2-associated X protein (BAX), BCL-2 homologous antagonist/killer (BAK) and BH3-only proteins and anti-apoptotic BCL-2 proteins [116]. A number of BH3-only proteins activate BAX or BAK and cause them to form pores in the inner membrane, ensuring MOMP. Anti-apoptotic BCL-2 proteins prevent the activation of BAX and BAK or directly sequester BH3-only proteins, thus preventing apoptosis despite death signaling [117].

Among the various regulatory mechanisms of the mitochondrial apoptosis pathway, the tumor suppressor protein p53 acts as a simultaneous BAX activator and BCL-2 suppressor [115]. By contrast, the PI3K/AKT pathway prevents apoptosis. Phosphatidylinositol-3-kinases (PI3K) can activate numerous downstream targets, including the protein kinase B (AKT), which is an important regulator of cellular proliferation, as it inhibits the pro-apoptotic activity of BAD, BAX, and glycogen synthase kinase-3 beta (GSK-3β) [118].

We identified several studies on the involvement of ncRNAs in mitochondrially mediated apoptosis in AD. Firstly, Guo et al. (2017) found that knockdown of the lncRNA BDNF-AS in Aβ-treated PC12 cells led to a significant upregulation of the mitochondrial anti-apoptotic marker BCL-2, alongside a reduction in pro-apoptotic proteins cleaved caspase-3 and BAX [79]. These findings complement preexisting literature. BDNF-AS has been shown to promote amyloid deposition [119] and mitochondrial dysfunction [120] and is upregulated in murine AD models [121] as well as in AD human blood [119]. BDNF-AS knockdown has been shown to suppress apoptosis and inflammatory pathway-related proteins in vivo [121].

Secondly, circ_0049472, a mainly cytoplasmic ncRNA, was upregulated in a cell model of AD, while miR-22-3p, which was shown to have neuroprotective and anti-apoptotic effects in other diseases [122], was downregulated [48]. Mechanistically, it was shown that circ_0049472 binds to miR-22-3p and that circ_0049472 knockdown resulted in the normalization of mitochondrial membrane potential (MMP), increased BCL-2 expression and decreased BAX and cleaved-caspase-3 [48].

Thirdly, in another study, neurons treated with Aβ−42o were exposed to extracellular vesicles containing miR-148a-3p, miR-21-5p, miR-103a-3p. This reduced BAX and BAD expression, increased anti-apoptotic BCL-2 levels, improved MMP, restored mitochondrial populations and normalized mitochondrial electron transport-related gene expression [67]. These results suggest that miR-148a-3p, miR-21-5p, miR-103a-3p exert protective effects on mitochondrial function and prevent mitochondrially mediated apoptosis, but further mechanistic confirmation is required to confirm which miR has the most powerful protective effect. miR-148a-3p is downregulated in AD patients’ serum [123, 124] and blood [125] and can be upregulated via exercise interventions [126]. Meanwhile, miR-21-5p levels in cerebrospinal fluid (CSF) were upregulated in a small group of AD patients [127] while whole blood and plasma studies report conflicting regulation patterns [128–130]. MiR-21-5p upregulation also indirectly protected against neuroinflammation and mitochondrial dysfunction in AD neurons treated with gold nanoparticles [81]. Similarly abundant studies are available on miR-103a-3p. It has been linked to several AD hub genes [131] as well as childhood trauma-related disorders [132], multiple sclerosis[133], and PD [134]. Abyadeh et al. (2025) identified links between miR-103 and mitophagy genes in AD, as discussed in another section of this article [54]. One other study found that miR-103a-3p is downregulated in AD fundamental models [135] but miR-103a-3p levels in patients’ serum were also lowered by exercise and cognitive rehabilitation [136]. Thus, the dysregulation pattern of miR-103a-3p has yet to be thoroughly investigated.

Fourth, Zhang demonstrated that H19 is upregulated while miR-129 is downregulated in the brains of AD mice. Silencing lncRNA H19 increased miR-129 levels, which in turn reduced p53 and Ki-67 expression, promoting Aβ25–35-induced PC12 cell viability [47]. Overall, the available clinical evidence on H19 dysregulation in AD [137] and mild cognitive impairment [138] is inconclusive. Lnc-RNA H19 demonstrates, however, broader mitochondrial relevance across various organ systems [139–142].

Fifth, miR-106b-5p is downregulated AD plasma [58] and whole-blood samples [143] and it has been designated as a hub miR in AD [144]. In their bioinformatics analysis, Nguyen et al. (2024) linked miR-106b-5p, miR-17–5p, and miR-16–5p to autophagy and immune signaling in the AD hippocampus [56]. There is evidence that miR-106b-5p promotes mitochondrial oxidative stress [145, 146] and modulates the BAX/BCL-2 and caspase-dependent apoptotic signaling pathways in SH‐SY5Y cells [146]. Therefore, future research could investigate the link between miR-106b-5p dysregulation and mitochondrial dysfunction in AD. miR-16-5p is upregulated in AD mouse brains [147], but the data on its pro-apoptotic effects via BCL-2 downregulation [147] is in conflict with its anti-apoptotic function mediated by BACE1 inhibition [148]. Comparatively less evidence exists for miR-17–5p; it demonstrates protective effects on mitochondrial autophagy in the myocardium [149] while disrupting the amyloid pathway in the microglia [150].

Sixth, experimentally induced overexpression of miR-9-5p in murine hippocampal neurons exposed to Aβ resulted in reduced oxidative stress via downregulated GSK-3β, which in turn decreased BAX and cytochrome C and increased BCL-2 [69]. Interestingly, another in vitro AD study concluded that miR-9-5p inhibits autophagy [151]. LncRNA BDNF-AS acts as a competing endogenous RNA for BACE1 by competitively binding miR-9-5p, which promotes amyloid pathology [119]. Further studies are necessary to clearly establish which molecular mechanism of interest should be employed therapeutically as far as miR-9-5p is concerned.

It must be noted that the direction of observed dysregulation trends of miR-9-5p across experimental models and patient tissue samples vary. For example, the above-mentioned study reports miR-9-5p downregulation in Aβ-treated neurons [69], whereas another study noted the opposite trend in the hippocampus of APP/PS1 transgenic mice [66]. In patient studies, Yuen et al. assessed miR-9-5p across eight independent human AD-versus-control brain comparisons spanning six brain regions through an omic dataset meta-analysis, but no general direction of dysregulation for miR-9-5p was established because the pooled analysis did not yield statistical significance [51]. Meanwhile, Cieślik et al. 2020 [57] used temporal-lobe tissue from three AD brains and three controls and observed miR-9-5p upregulation in AD brains [57]. Further diverging reports come from exosome-enriched compared to raw AD CSF [152] and AD brain tissue datasets [153]. These discrepancies may be associated with differences in sample sizes and types, choice of sampled brain region and the various factors influencing in vivo transgenic AD mouse microRNA signatures compared to cell monocultures.

So far, we have discussed the ncRNAs relevant to the mitochondrial apoptosis pathway in AD. In the following, we focus on autophagy. An additional important regulator of autophagic flux and mitochondrial bioenergetics is GABA type A receptor-associated protein-like 1 (GABARAPL1), which is an Atg8-family protein contributing to the later stages of autophagosome maturation [154, 155]. GABARAPL1 is downregulated in the human AD frontal lobe [156]. Loss of GABARAPL1 impairs autophagic flux and mitochondrial clearance, leading to the accumulation of dysfunctional mitochondria [154]. Our review identified that several miRNAs are relevant to GABARAPL1 function in AD.

Xu et al. (2024) identified GABARAPL1 as an AD-associated biomarker, with its downregulation confirmed across human AD transcriptomic datasets. Bioinformatic analyses performed within the same study revealed that miR-195-5p, miR-16-5p, miR-155-5p, miR-497-5p, miR-15b-5p, miR-143-3p, and miR-133b are predicted to target GABARAPL1 [62]. MiR-195-5p was additionally linked to neuroinflammation and amyloid toxicity in another study investigating differentially expressed miRNAs in AD [51]. Among these miRNAs predicted to target GABARAPL1, several have also been reported as dysregulated in human AD samples, as will be discussed below. Plasma, serum or whole blood dysregulation has been documented for miR-195-5p and miR-16-5p [157], miR-497-5p [158], miR-15b-5p [159, 160], miR-143-3p [161, 162], and miR-133b [163, 164]. MiR-195-5p [165] and miR-155-5p are upregulated in the AD human brain [166], while miR-16-5p was additionally dysregulated in CSF [167].

The second autophagy regulator of interest is TFEB (transcription factor EB), which acts as a major transcriptional regulator of autophagy-lysosomal genes [168]. TFEB activity is inhibited by GSK3β and mTOR signaling, which promotes Aβ and phosphorylated tau accumulation and cognitive decline in AD [112]. TFEB also regulates electron transport chain complex I, supports mitochondrial biogenesis via transcriptional regulation and also ensures the expression of mitophagy receptors nuclear dot protein 52 (NDP52) and optineurin (OPTN) [169]. TFEB has been validated as an AD biomarker in SH-SY5Y neuronal cells and is predicted to be targeted by miR-29a-3p, miR-124-3p, miR-29b-3p, and miR-29c-3p [62]. One in vitro study used miR-124-3p mimics to increase GSK-3β phosphorylation and consequently reduce tau protein-induced apoptosis [170]. miR-29b-3p exhibited significant downregulation in the whole blood of PD but not AD patients [171]. By contrast, miR-29c-3p was not only downregulated in the serum of AD patients, but was also shown to upregulate the mitochondrial epistasis gene signal transducer and activator of transcription 3 (STAT3) activity in vitro and improve cognitive function in rats [172]. Mesenchymal stem cell-derived extracellular vesicles containing miR-29c-3p ameliorated AD pathology in vivo and in vitro. The therapeutic effect of miR-29c-3p was mediated by BACE1 inhibition [173].

As with apoptosis and autophagy, there are ferroptosis-relevant ncRNAs implicated in AD pathophysiology. Ferroptosis is a cell death mechanism which is iron-dependent and reactive oxygen species-reliant [174]. It promotes Aβ, hyperphosphorylated tau aggregation [175] and neurodegeneration [176]. The AD brain displays increased iron deposition [177]. Iron is necessary for mitochondrial electron transport chain function, heme biosynthesis, such that disruptions in iron homeostasis also induce mitochondrial dysfunction [178].

We identified one study which investigated the involvement of miR-7a in ferroptotic phenomena and the effect thereof on the mitochondria. Ramesh et al. (2025) showed that miR-7a was upregulated in AD mice cortices and hippocampi while the pro-inflammatory Krüppel-like factor 4 (Klf4) was upregulated in AD mouse brain tissue. MiR-7a mimics decreased Klf4 protein expression in untreated SH-SY5Y cells while, in a separate experiment, ferroptosis-induced SH-SY5Y cells displayed increased Klf4 protein. MiR-7a mimic treatment of SH-SY5Y cells followed by ferroptosis induction prevented mitochondrial fragmentation and restored MMP levels. Importantly, the cells used in the above experiments were untreated with Aβ oligomers, nor was miR-7a expression evaluated following Aβ oligomer treatment [65]. The observed upregulation of both miR-7a and Klf4 in AD mice coupled with the protective effects of miR-7a mimics in ferroptosis models could be interpreted as follows: the observed miR-7a upregulation in vivo plays a partial compensatory role in the context of AD-related Klf4 overexpression, as also noted by the authors [65]. Klf4 belongs to the family of zinc finger transcription factors with various regulatory functions [179], such as iron level modulation and lipid peroxidation [65]. The mitochondria has been implicated in the initiation and mediation of ferroptotic signaling due to its role in mtROS production, iron accumulation [180], the TCA cycle and electron transfer chain activity [181]. Conversely, ferroptotic stimuli lead to mitochondrial morphology and metabolism abnormalities [182].

To summarize this section, preclinical AD model data indicates that lncRNA BDNF-AS [79] and circ_0049472 [48] promote mitochondrial-dependent apoptosis by downregulating BCL-2 and upregulating BAX and caspase-3. Conversely, miR-148a-3p, miR-21-5p, miR-103a-3p [67] exert the opposite effect, preventing apoptosis. LncRNA H19 silencing results in microRNA-129 upregulation and consequent anti-apoptotic effects in vitro via the regulation of p53 and Ki-67 expression [47] and other experimental data from non-AD models point to its involvement in the regulation of PTEN and PINK1 [123, 139, 142]. Additionally, miR-7a seems to attenuate ferroptosis-associated mitochondrial fragmentation [65], but no clinical data is available on its dysregulation in AD patients, although decreased miR-7 expression has been observed in PD post-mortem brains [183]. Bioinformatic evidence converging with preexisting mechanistic and clinical studies further implicate miR-106b-5p in autophagic regulation in AD [56], possibly via BAX/BCL-2 signaling and mitochondrial oxidative stress [146]. Similarly, miR-195-5p, miR-16-5p, miR-155-5p, miR-497-5p, miR-15b-5p, miR-143-3p, and miR-133b are predicted regulators of GABARAPL1 [62], which importantly regulates autophagosome formation and mitochondrial clearance [154, 155]. TFEB, another major autophagy regulator which also regulates mitophagy receptor expression [112, 169] is predicted to be targeted by miR-29a-3p, miR-124-3p, miR-29b-3p, miR-29c-3p in AD [62]. These predicted miRNA-target relationships warrant further experimental validation to clarify their involvement in autophagic and mitochondrial dysregulation in AD.

### NcRNAs Influence Mitochondrial Biogenesis and Mitochondrial Gene Expression in AD

Altered mitochondrial biogenesis is one of the causes for reduced mitochondrial populations in AD hippocampi [184]. Multiple mitochondrial transcription factors and coactivators are involved in mitochondrial biogenesis regulation. PGC-1α plays a central role due to its role in activating mitochondrial gene transcription and function. Two key regulators of PGC-1α in low energy conditions are the energy sensor AMP-activated protein kinase (AMPK), which activates PGC-1α via phosphorylation when the AMP/ATP ratio increases, and SIRT1, which deacetylates PGC-1α when the NAD+/NADH ratio increases [185]. Our study identified that several ncRNAs intervene in this mitochondrial regulatory axis in AD.

MiR-34a, miR-146a, and miR-155 [57] and miR-9-5p [57, 66] are negative regulators of SIRT1 while miR-485 downregulates PGC-1α [55] in AD. MiR-9-5p, miR-132-3p, and miR-212-3p all have been shown to inhibit SIRT1 in other disease models [186–189]. MiR-9-SIRT1 interactions have previously been reported in neurodegeneration studies [189]. MiR-132-3p inhibition conserves SIRT1-PGC-1α-NRF2 signaling in oxidative stress [187]. Below, we will discuss reports on the dysregulation of these miRNAs in AD, having already addressed miR-9-5p dysregulation trends in the previous section. MiR-485 is a highly conserved brain-enriched miR whose dysregulation in the AD brain [55], blood and CSF [190], salivary exosomes [191] as well as in PD [190] has been previously reported. Notably, miR-34a, miR-146a, miR-155 [57] and miR-485 [55] were significantly upregulated in AD patient brains. Finally, Yuen et al. observed statistically significant dysregulation for miR-132-3p, miR-212-3p in AD brains their meta-analysis [51].

Alongside PGC-1α, mitochondrial genome maintenance exonuclease 1 (MGME1) is also involved in mitochondrial gene expression. It encodes an exonuclease responsible for mtDNA processing and mitochondrial genome maintenance [192, 193]. There is evidence that links mtDNA damage in AD to energy depletion and increased oxidative stress [194]. Our review identified a bioinformatics study which showed that MGME1 is significantly downregulated in the human AD brain and that miR-922 can bind to the 3′ UTR of MGME1 and inhibit its expression [53].

Finally, Xu et al. (2025) found miR-132 and miR-129-5p to be downregulated in AD brains and identified potential interactions between miR-124-3p and the mitochondrial epistasis genes STAT3 and cyclin-dependent kinase 6 (CDK6) [52]. STAT3 acts as a transcription factor which regulates mitochondrial calcium homeostasis, respiration [195], electron transport chain activity [196], and participates in mtDNA transcription [197, 198]. Additionally, it has an anti-apoptotic function, stimulating BCL-2 transcription [199]. CDK6 regulates exit from the G1 cell cycle phase [200]. It has been shown to interact with the promoters of oxidative phosphorylation (OXPHOS) genes and one mitochondrial transcriptional regulator [201]. One murine study on neurotoxicity confirmed that miR-124-3p downregulates STAT3 and lowers neuroinflammation [202]. Interestingly, serum miR-124-3p is upregulated by rosuvastatin in AD patients [203]. Its upregulation was also reported in post-mortem AD human brains [204]. Therapeutically, exosomally delivered miR124-3p attenuated neuroinflammation in a triculture AD model [205] and exerted neuroprotective effects in vitro and in vivo in other AD studies [170, 206]. MiR124-3p dysregulation has also been observed in PD [207] and traumatic brain injury [208, 209].

The STAT3/miR-204/transient receptor potential mucolipin-1 (TRPML1) signaling axis was also implicated in mitochondrial autophagy and oxidative stress regulation in a study by Zhang (2021), wherein miR-204 downregulation by STAT3 resulted in increased TRPML1 expression, reduced mitochondrial dysfunction and ROS production and promoted mitophagy in AD [71]. Li (2025) developed CeO2-DNA-RNA hybrid chain (DRP)/mitochondrial aptamers (MA) nanoclusters designed to silence mitochondrial miR-204. In vitro, treatment improved mitochondrial membrane potential and ROS levels and ameliorated AD-related pathological TRPML1/STAT3 autophagic signaling. In AD mice, the nanoclusters accumulated in brain tissue and exerted similar effects on hippocampal autophagic signaling. However, miRNA-204 levels were only qualitatively assessed via fluorescence studies in mice and no fold changes were reported for the RT-qPCR data on the Aβ-treated SY5Y cells [85]. One clinical study found that miR-204 was downregulated in neuron-derived serum exosomes in AD patients [210] while blood lymphocyte miR-204 was upregulated in another study [211]. MiR-204 downregulation in CSF has been reported in frontotemporal dementia [212] as well as AD [213].

MiR-423 was upregulated in the hippocampus and medial frontal gyrus, whereas miR-98 was downregulated in the cerebellum of AD patients [84]. In vitro studies in non-AD cell lines confirmed that miR-423 and miR-98 downregulate isocitrate dehydrogenase 2 (IDH2), which helps maintain mitochondrial redox balance. IDH2 levels in the selected brain regions were, however, not measured directly [84]. Lower plasma levels of miR-423-5p correlated with cognitive deterioration and increased amyloid deposition in AD patients [214] and experimental evidence from non-AD models supports miR-423-5p involvement in the regulation of mitochondrial function [215, 216]. MiR-98 reportedly displays dysregulated expression in AD patients [217] and has also demonstrated a connection with mitochondrial dysfunction in a scopolamine model of AD [218].

In summary, given that SIRT1 exerts a protective effect on mitochondrial biogenesis, hippocampal-dependent memory and helps ameliorate neurotoxic Aβ peptide accumulation [219], the modulation of miR-9, miR-34a, miR-146a, miR-155 [57], miR-9-5p [51, 66], miR-132-3p, and miR-212-3p may be a viable therapeutic approach in optimizing mitochondrial biogenesis in AD. MiR-485 [55] antagomir treatments to stimulate mitochondrial biogenesis by increasing PGC-1α could also be of great research interest given the broadly observed dysregulation of miR-485 in the blood, CSF and brain of AD patients. MiR-204 inhibition via STAT3 [71] and nanoclusters [85] has positive effects on mitochondrial autophagy and mitochondrial function, respectively. As human full-length tau accumulation [220] and Aβ pathology [221] influence STAT3 levels, miR-204 antagomirs could be employed to compensate for the detrimental effects of low STAT3 on mitochondrial autophagy. MiR-423 and miR-98 may influence mitochondrial redox balance [84]. Additionally, the preliminary bioinformatic data suggest that miR-922 may contribute to MGME1 downregulation [53] and to the accumulation of mitochondrial genome defects in AD. Finally, given the documented involvement of miR-124-3p in neuroinflammatory signaling [222], mitochondrially-mediated apoptosis [223] and mitochondrial localization processes [224] in the context of neurodegeneration, the bioinformatic findings of Xu et al. regarding the regulation of STAT3 and CDK6 by miR-124-3p [52] also require additional experimental validation.

### NcRNAs Regulating Mitochondrial Dynamics in AD

Disturbances in the expression of mitochondrial fusion and fission genes have been observed across neurodegenerative diseases [225]. The AD human hippocampus displays altered mitochondrial dynamics with intensified mitochondrial fission [226] while therapeutic measures stimulating fusion may ameliorate disease course [227].

Mitochondrial fusion involves the fusion of two mitochondrial outer membranes via GTP hydrolysis and a subsequent fusion of the inner membranes. In mammals, there are three key dynamin-related GTPases controlling mitochondrial fusion: mitofusin 1 (MFN1), mitofusin 2 (MFN2), and optic atrophy 1 (OPA1). MFN1/MFN2 fuse the outer membranes, while OPA1 ensures the fusion of the inner membranes [228].

Mitochondrial fission is coordinated by dynamin-related protein 1 (DRP1), a cytosolic protein recruited by various mitochondrial adaptors to form an oligomeric ring around the constricted mitochondria. DRP1 undergoes GTP hydrolysis and dynamin 2 is recruited to the constricted site to complete the division. Inner mitochondrial membrane constriction takes place at mitochondria-ER contact regions and depends on Ca^2+^ [229]. Mitochondrial fission protein 1 (FIS1) is a mitochondrial outer membrane protein which is involved in the fission complex [230] and facilitates DRP1 recruitment and assembly [231]. It has also been shown to inhibit MFN1, MFN2 and OPA1 activity [232]. Miro1 and Miro2 are outer mitochondrial membrane GTPases involved in inter-organelle interactions, mitochondrial transport on microtubule and actin cytoskeleton and mitophagy [233]. Miro proteins inhibit mitochondrial fission in the presence of normal Ca^2+^ concentrations and promote fission in the presence of high Ca^2+^ via DRP1 [234].

Our systematic review has identified several miRNAs which affect mitochondrial dynamics in AD. Woo et al. (2020) demonstrated that Miro2 is a critical target of miR-351-5p. Murine AD hippocampal tissue showed upregulated miR-351-5p and downregulated Miro2, a finding that was supported by transcriptomic data. In HCN cells, miR-351-5p was shown to directly downregulate Miro2 mRNA, leading to increased mitochondrial fragmentation and dysfunction and autophagy-dependent cell death mediated via PINK1/Parkin activity [63]. In another study, across human and 3xTg-AD model mice hippocampi and HCN cells, Han et al. (2024) observed a downregulation of OPA1 and MFN2. This downregulation was linked to the upregulation of miR-323-5p and miR-370-3p, observed in both 3xTg-AD mice and in HCN cells following insulin withdrawal. These microRNAs decreased OPA1 expression, while miR-150-3p was additionally shown to directly target MFN2, promoting mitochondrial fragmentation and neuronal cell death [61].

Zhang et al. bring complementary results by finding that miR-195 was upregulated in the SAMP8 mice and that miR-195 inhibits MFN2 translation and therefore mitochondrial fusion [70]. There is evidence to support the therapeutic potential of miR-195 inhibition in AD. Intraventricular delivery of miR-195 inhibitors improves cognitive performance, enhances postsynaptic density, and increases the activity of mitochondrial respiratory chain complexes I and II in AD mouse models, supporting its role as a regulator of mitochondrial and synaptic function [235].

Conversely, the studies of Kumar et al. showed that miR-455-3p upregulates OPA1, MFN1, and MFN2 and downregulates the fission-associated genes DRP1 and FIS1 in mouse neuroblastoma cells transfected with mutant APP [74] and ameliorates mitochondrial dynamics and biogenesis in mice [236]. These findings are in agreement with a subsequent study which confirms the beneficial effect of miR-455-3p on mitochondrial dynamics and mitophagy [237]. Furthermore, miR-455-3p dysregulation has been observed across post-mortem AD brains, B-lymphocytes, and fibroblasts [238, 239], making it a potent biomarker and therapeutic development focus. On the other hand, lncMtDloop, which was found to be downregulated in human and murine AD mouse brains, was also proved to promote fusion by increasing OPA1 and to decrease fission by downregulating DRP1 [75], but additional clinical and preclinical data is necessary.

Vijayan et al. (2024) found miRNA PC-5P-12969 to be elevated in the human AD frontal cortex, in APP and tau transgenic mice, Aβ knock-in mice as well as in HT22 cells treated with mutant APP (mAPP). MiRNA PC-5P-12969 was shown to downregulate GSK3 and ameliorates mitochondrial respiration, length and ATP production while lowering mitochondrial fragmentation in mAPP-treated cultures [64]. GSK3 is a multifunctional Ser/Thr protein kinase which is dysregulated neurodegeneration [240]. GSK-3β overexpression negatively regulates mitochondrial biogenesis via PGC-1α and PI3K/Akt signaling, promotes mitochondrial fragmentation by increasing DRP1 and mitochondrial fission factor and disturbs mitochondrial bioenergetics by suppressing complex I activity [241].

Wang et al. (2019) found that lncRNA SNHG1 knockdown upregulates miR-137, which in turn contributed to neuroprotection by repressing kringle-containing transmembrane protein 1 (KREMEN1) expression in Aβ-treated neurons [45]. KREMEN1 is involved in the Wnt/β-catenin pathway regulating metabolism and homeostasis [242] and one of its cytoplasmic regulatory components, GSK-3β, has been shown to exhibit regulatory effects on mitochondrial activity [241, 243]. MiR-137 dysregulation has been linked to AD progression [244, 245], mitochondrial biogenesis [246] and mitophagy [247], but the neuroprotective role of miR-137 is not supported across all reports.

Garcia et al. (2021) found that miR-146a was upregulated in SH-SWE cells and downregulated in PSEN1ΔE9 mutated neurons [72]. MiR-124 mimic resulted in a downregulation of exosomal miR-125b/miR-21/miR-146a/miR-155 and an increase in mitochondrial transmembrane potential in both of the above-mentioned cell lines. Interestingly, miR-124 inhibition promoted mitochondrial fusion by increasing MFN2 levels [72]. Another study using an in vitro model of chronic neuroinflammation found that TNF-α exposure in hippocampal neurons results in elevated miR-34a and miR-146a, which correlated with mitochondrial dysfunction [248]. MiR-34a is also elevated in d-galactose (d-gal)-induced oxidative stress. Interventions like swimming and miR-34a inhibitors resulted in decreased hippocampal DRP1 and MFN2, as well as increased mitochondrial biogenesis (PGC-1α) in rats [249]. Elsewhere, it was reported that miR-146a downregulates Parkin [250]. Both miR-34a and miR-146a downregulate BCL-2 [251]. Therefore, miR-146a antagomirs [252] could restore mitophagic balance and mitochondrial function in the AD brain. miR-146a therapies have been preliminarily investigated in relation to cortical impact injury [253] and ALS [254].

Briefly, in the preclinical AD studies, we have identified that mitochondrial fission is promoted by miR-351-5p via Miro2 downregulation [63], by miR-150-3p, miR-323-5p, and miR-370-3p via OPA1 and MFN2 downregulation [61], and by miR-195 [70] and miR-124 [72] via MFN2 inhibition. By contrast, miR-455-3p [74] and lncMtDloop [75] upregulate fusion-related genes, while miRNA PC-5P-12969 [64, 241] and miR-137 [45] seem to reduce mitochondrial fission rates indirectly by downregulating GSK3.

### NcRNAs Regulating Mitochondrial Protein Import in AD

Amyloid precursor protein causes mitochondrial protein import dysfunction by blocking the translocase of the outer membrane (TOM) and translocase of the inner membrane 23 channels (TIM23), which causes Complex IV dysfunction and favors ROS production. Aβ also prevents the entrance of precursor proteins through the mitochondrial outer membrane [255]. APP accumulation in the import channels is proportional to AD severity and to mitochondrial dysfunction [256]. In this section, we summarize the available data on ncRNA regulation of the mitochondrial import machinery in AD.

There are five mitochondrial protein import pathways, and the classical import pathway relies on TOM and TIM23 [257]. TOMM20 is part of the translocase of the outer mitochondrial membrane complex (TOMM). TOMM20 functions as a receptor for mitochondrial precursor proteins and nuclear-encoded oxidative phosphorylation subunits. Dysregulated TOMM20 expression has been linked to ischemic injury damage, Meniere’s disease and cancer [258]. Based on bioinformatics analyses, TOMM20 was predicted to be targeted by Let-7a-2-3p and miR-204-3p in AD [62]. Little data is available regarding these miRNAs in AD: miR-204-3p is downregulated in the murine AD hippocampus and relieves oxidative stress [259] and neuroinflammation [260]. Let-7a-2-3p is downregulated in the epileptic murine hippocampus [261] but has not been quantified in AD models.

TIMM13 is part of the translocase of the inner membrane (TIM) complex and plays a role in mitochondrial import and biogenesis [262] as well as mitophagy [263]. Kim et al. (2021) observed TIMM13 downregulation in the hippocampi of AD patients. In H4-APPswe and SH-SY5Y cells, miR-1273g-3p directly downregulated TIMM13 [58]. Only one other preclinical study investigated miR-1273g-3p, namely, as an autophagic promoter in SH-SY5Y cells [264]. Additional clinical validation of the biomarker potential of this miR is necessary and more detailed descriptions of its role in mitochondrial protein import are warranted.

Voltage-dependent anion channel 1 (VDAC1) is an outer mitochondrial membrane protein responsible for metabolite, ROS, and Ca^2+^ transport as well as apoptotic signaling via BAX and BCL-2 [265]. VDAC1 upregulation has been reported in the AD post-mortem brain and in APP mice [266]. In AD, VDAC1 interacts with Aβ and phosphorylated tau [267], causing mitochondrial pore dysfunction, which affects protein import into the mitochondria and downstream oxidative phosphorylation and structural abnormalities [266]. In the temporal cortex of AD patients, miR-146a levels were upregulated and inversely correlated with VDAC1 expression. MiR-146a compromised oxidative phosphorylation in mixed glial cells exposed to Aβ [59]. Since VDAC1 downregulation seems to ameliorate mitochondria function in AD mice, [268, 269], VDAC1 modulation via mir-146a may be an attractive research direction.

Finally, in a familial AD cell line carrying the PSEN1 L150P mutation, circ_0001946 was found to be upregulated. Circ_0001946 downregulates ubiquitin-conjugating enzyme E2A (UBE2A) [49]. UBE2A encodes a neuronal-specific ubiquitin conjugating enzyme [270] which promotes the mitochondrial translocation of Parkin [271] and also ubiquitinates mitofusins, TOM20, TOM70 and VDAC1 [272]. In AD, outside of its mitochondrial function, UBE2A has been shown to contribute to the proteolytic clearance of amyloid peptides [273]. At the time of writing this article, no additional studies regarding circ_0001946 in relation to AD or the mitochondria could be identified, although circ_0001946 has been studied in glioblastoma and other cancers [274, 275].

Altogether, the strongest experimental and clinical evidence supporting ncRNA involvement in mitochondrial protein import dysfunction concerns miR-146a and miR-1273g-3p as downregulators of VDAC1 and TIMM13, respectively. A singular study suggests that circ_0001946 downregulates UBE2A and thus indirectly helps regulate TOM and VDAC1 expression while only bioinformatic data nominate Let-7a-2-3p and miR-204-3p as regulators of TOMM20 in AD.

### NcRNAs with Emerging Mitochondrial Roles in AD

**Fig. 3:**
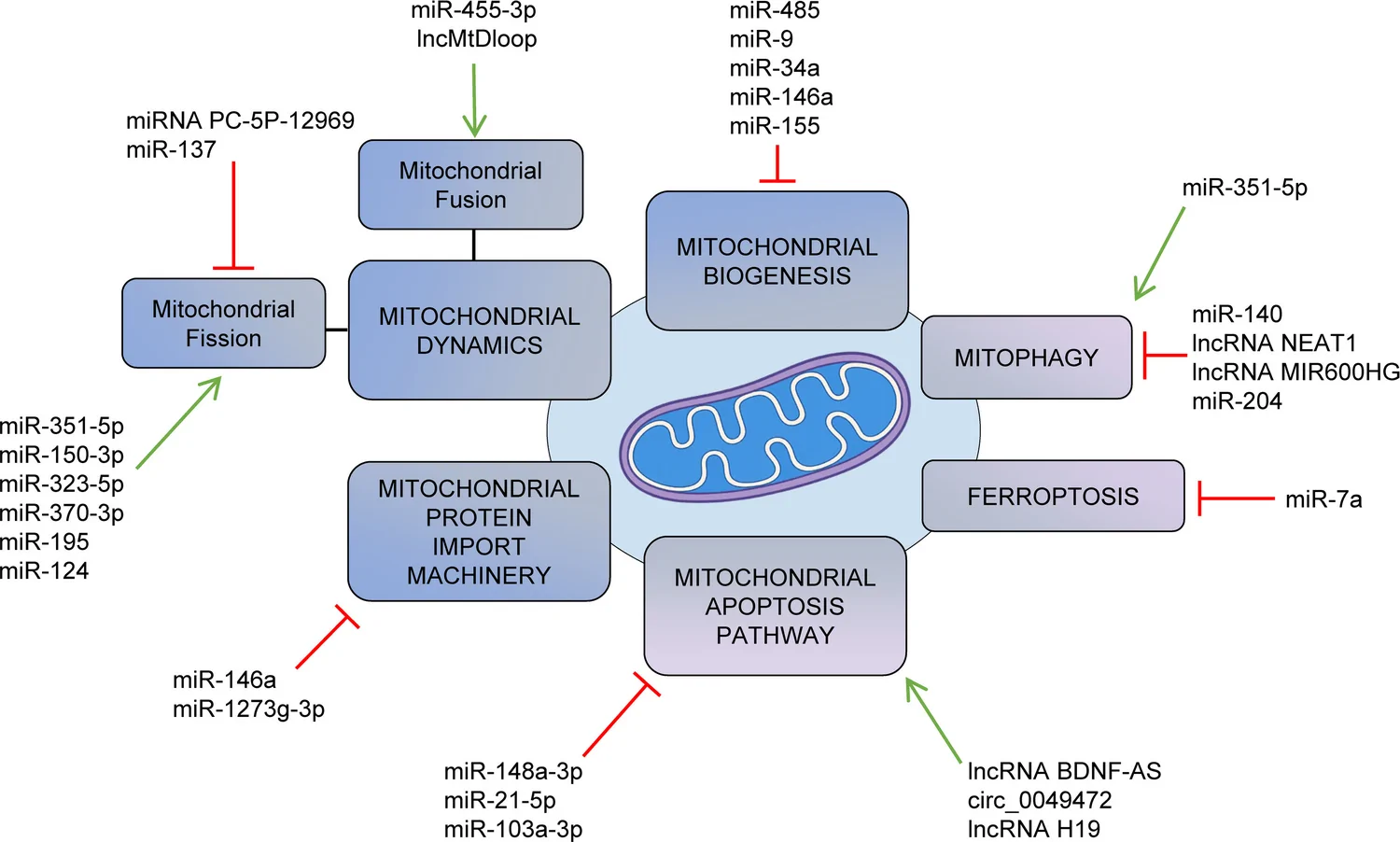
Schematic representation of the major ncRNAs which directly regulate mitochondrial functions in Alzheimer’s disease (AD). The figure summarizes the key miRNAs, lncRNAs, and circRNAs reported to modulate mitochondrial dynamics, biogenesis, mitophagy, ferroptosis, apoptosis, and protein import in AD. Arrows indicate activation or positive regulation, whereas T-bar-ended lines indicate inhibition or negative regulation

The major ncRNAs directly implicated in AD mitochondrial regulation and the mechanisms at play have been synthesized in Fig. 3. In this section, we discuss several ncRNAs of emerging mitochondrial relevance in AD mediated by oxidative stress, neuroinflammation, and endoplasmic reticulum-mitochondria interactions. The most abundant evidence involves miR-375. A bioinformatics analysis, whose findings were replicated in Aβ-treated SH-SY5Y cells and AD mice, identified miR-375 upregulation in the cortex of AD patients while lncRNA WT1-AS and the target gene of miR-375—transcription factor SIX homeobox 4 (SIX4)—were downregulated [46]. Interestingly, another study found that miR-375 was upregulated in the plasma and CSF of aMCI but not in AD patients [58], which points to the need of establishing the expression profiles of miR-375 across distinct tissue types in AD. Wang et al. further showed that lncRNA WT1-AS overexpression suppressed miR-375 and consequently upregulated SIX4 while improving mitochondrial oxidative status and ATP production in vitro. [46] Thus, the miR-375/SIX4 axis may be indirectly involved in mitochondrial homeostasis. Conversely, miR-375-3p mimic treatment seems to improve neuronal mitochondrial function in Aβ-treated neuroblastoma cells and promote mitochondrial autophagy in AD mice [82] without promoting apoptosis in AD [276]. The diverging findings in the effects of miR-375 on mitochondrial function in AD reported in the two studies could be explained by differences in protocols. Wang et al. (2020) differentiated SH-SY5Y cells with retinoic acid and BDNF [46] while the second study does not mention pre-treatment of SH-SY5Y cells before Aβ exposure [82]. It is additionally noted that under physiological conditions, miR-375 can inhibit autophagy, while in early AD pathogenesis, it exerts the opposite effect [82]. The differences in signaling pathway studied and cell culture likely contribute to the differences in mitochondrial outcomes. Thus, the mechanistic details of the effects of miR-375 on mitochondrial function in AD require further investigation.

To continue, there is evidence of miR-218-5p, miR-124-3p downregulation in the human and murine AD brain. These miRNAs downregulate the endoplasmic reticulum protein reticulon 3 (RTN3), which regulates mitochondrial fission and influences neuronal mitochondrial localization [60]. Neuronal RTN3 is upregulated in AD and Parkinson’s disease as a nonspecific endoplasmic reticulum stress response [277]. RNT3 promotes BCL-2/Beclin-1 interactions [278] and mediates BCL-2 translocation to the mitochondria under endoplasmic reticulum stress [277]. In APP-transfected SH-SWE cells, miR-124-3p also inhibits receptor for advanced glycation end-products (RAGE)/high mobility group box 1 (HMGB1) axis signaling, which alleviates neuroinflammation and indirectly optimizes mitochondrial MMP [73, 279]. Hence, miR-124-3p indirectly influences mitochondrial function by regulating endoplasmic reticulum-mitochondria interactions and neuroinflammation signaling.

Meanwhile, several downregulated lncRNAs demonstrating dysregulation in AD have been directly linked to mitochondrial processes in other disease models. LncRNAs MEG3, LOXL1-AS1, ADAMTS9-AS2, and A2M-AS1 are downregulated in a preclinical AD study by Corsi et al. [49]. MEG3 and ADAMTS9-AS2 dysregulation in AD has also been reported in other studies [280–282]. MEG3 [283] and A2M-AS1 [284] have been linked to mitophagy while LOXL1-AS1 helps regulate mitochondrially mediated apoptosis [285, 286]. ADAMTS9-AS2 appears to indirectly regulate pyroptosis [287], an inflammatory cellular death pathway which causes mitochondrial damage [288]. Overall, MEG3 and ADAMTS9-AS2 show promise as potential AD biomarkers and mitochondrial regulators.

A mouse study suggests that the miR-96-5p/Foxo1/Lcn2 axis may be involved in mitochondrial dysfunction and oxidative stress in AD. MiR-96-5p inhibition upregulated Foxo1 and downregulated Lcn2 in AD mouse osteoblasts while reducing AD mouse hippocampal oxidative stress. Foxo1 overexpression and Lcn2 silencing separately improved hippocampal mitochondrial ultrastructure [86]. Serum miR-96-5p and neuronal inflammatory mediator Cathepsin B were shown to have good diagnostic accuracy for AD-related cognitive impairment [289] and miR-96-5p inhibition was seems to promote Aβ clearance [290]. The literature thus suggests miR-96-5p involvement in AD, making the future confirmation the direct impact of miR-96-5p inhibition on mitochondrial parameters in AD a reasonable research objective.

Two preclinical studies investigate circ_0004381 and lncRNA-ATB, which could potentially be used to target AD-related mitochondrial dysfunction induced by oxidative stress. Firstly, Li et al. observed that the knockdown of circ_0004381 in AD neurons reduced COX-2 (cyclooxygenase-2) expression and microglial iNOS (inducible nitric oxide synthase) [80], the latter being involved in inflammatory neurodegeneration [291] and mitochondrial dysfunction [292]. Secondly, lncRNA-ATB was upregulated in Aβ25–35 induced PC12 cell injury and in turn repressed miR-200, which resulted in oncogene zinc finger protein 217 (ZNF217) overexpression, oxidative stress and MMP reduction [76]. ZNF217 suppresses PI3K-AKT activation in Aβ-damaged neurons [293], which increases oxidative stress and mitochondrial vulnerability in neurons [294]. While no clinical data are available for circ_0004381, lncRNA-ATB upregulation has been reported in the CSF and serum of AD patients.

A small clinical study observed the downregulation of miR-6501-5p and miR-143-3p in the whole blood of AD patients compared to controls. These miRNAs were predicted to target mitochondrial genes ATP5E and NDUFB9, NDUFA9, NDUFS1 and COX4I1, respectively, contributing to the regulation of the various functions such as the electron transport chain and respiration [83]. So far, miR-143-3p has been linked to mitochondrial apoptosis in a myocardial ischemia model [295].

Two multiomics studies provide additional research avenues concerning ncRNA-mitochondrial gene interactions. One multiomics study predicted that miR-103 and miR-484 are coregulatory miRNAs for several AD-relevant oxidative phosphorylation and tricarboxylic acid cycle genes [54]. In AD patients, miR-103 demonstrates downregulation in plasma [296] while miR-484 is downregulated in brain tissue [297]. In other disease models, miR-484 was linked to oxidative phosphorylation [298] and mitochondrial fission [297, 299] while miR-103 inhibited mitophagy [134, 300]. Another bioinformatic study proposes miR-7113-5P and miR-3130-3P as regulators of ABAT gene AD-risk alleles [301]. ABAT (4-aminobutyrate aminotransferase) interacts with APOE, potentially contributing to AD pathogenesis [302], and is involved in several mitochondrial processes, from the catabolism of inhibitory neurotransmitter γ-aminobutyric acid to OXPHOS enhancement [303, 304]. However, the dysregulation of miR-7113-5P and miR-3130-3P in AD has yet to be clinically and experimentally confirmed.

Lastly, the downregulation of the cytoplasmic miR processing enzyme Dicer1 due to amyloid pathology appears to impact mitochondrial homeostasis indirectly by inducing oxidative stress [50]. Dicer1 exerts protective effects on mitochondrial metabolism [305, 306] and OXPHOS [307] in several preclinical AD studies. This is an incentive to further study Dicer1 expression in the post-mortem AD brain and to identify the main mechanism by which Dicer1 ameliorates mitochondrial function in AD.

A number of studies not included in our systematic data extraction because their models simulated limited aspects of AD neuropathology indicate that additional ncRNAs may be considered in the exploration of mitochondrial dysfunction in future research. MiR-98 indirectly prevented mitochondrial apoptosis pathway activation in a scopolamine AD-like model [218]. Several studies using AlCl_3_ to induce AD-like neurodegeneration indicate a possible link between miR-134 and altered mitochondrial function as a result of oxidative stress [308–310]. Lnc-NKILA similarly exacerbated oxidative stress and lowered MMP in streptozotocin-induced neuronal injury [311]. While the experimental models employed above do not ideally reflect the characteristics of AD, these mechanistic insights could inform future in vitro and in vivo studies verifying the above-mentioned mechanism in AD models of higher histopathological fidelity.

To summarize this section, we have identified several ncRNAs of potential mitochondrial relevance in AD. There is evidence of miR-218-5p, miR-124-3p, miR-375, miR-103, miR-484, MEG3, ADAMTS9-AS2, miR-96-5p, miR-6501-5p, and miR-143-3p dysregulation in AD models or patients. MiR-218-5p and miR-124-3p regulate endoplasmic reticulum-mitochondria interactions while miR-124-3p additionally inhibits neuroinflammation. Preliminary in vitro data supports the indirect involvement of miR-375 in mitochondrial autophagy in AD, although the exact mechanisms and pathways require further clarification. Despite the documented mitochondrial relevance of miR-103, miR-484, and MEG3 in other pathologies, there is no confirmatory experimental data using AD models. Less evidence is available for circ_0004381, lncRNA-ATB and miR-96-5p which seem to promote mitochondrial dysfunction in vitro by exacerbating oxidative stress, and for miR processing enzyme Dicer1, which has so far not been studied in patient populations, and for miR-7113-5P, miR-3130-3P, which are predicted to target the ABAT gene.

### Limitations and Future Directions

The current study has various limitations. Firstly, the heterogeneity of experimental models and relatively small sample sizes of the translational studies made it impossible to perform a meta-analysis on the collected data. A risk of bias or quality assessment was not performed due to the diverse nature of study designs and documented outcomes, of which some were qualitative and some quantitative. The systematic review protocol has not been pre-registered in a platform such as PROSPERO. Secondly, various discrepancies in the RNA quantification methods and in the observed dysregulation patterns of certain ncRNAs between studies indicate that further research using uniform methodology is needed to establish their pathogenetic relevance. Thirdly, given the intricate relationships between amyloid pathology, oxidative stress and mitochondrial dysfunction [312], whether ncRNA dysregulation is a cause or a consequence of AD and related disorders remains to be determined [313].

Fourth, only a minority of translational studies employed plasma or CSF samples while many used post-mortem transcriptomic data, making it a priority for future AD ncRNA research to focus on larger patient cohorts and to explore body fluid diagnostic methods. In line with this issue, the sample sizes employed in the translational studies were relatively small and larger cohorts will be necessary to validate preexisting findings in human subjects. Notably, there are limited large-scale [314, 315] and longitudinal [316, 317] studies investigating the interplay between ncRNA levels and the evolution of AD. Further studies will be necessary to reach standardized biomarker expression level thresholds.

Additionally, although certain ncRNA knockdown and overexpression methods have yielded promising results in the preclinical studies we identified, cell and rodent AD or neuroinflammation models do not reflect the entirety of the biological states involved in human disease and, as such, preclinical data requires validation in humans. In a similar fashion, ncRNAs which show therapeutic promise pose the challenge of firstly configuring a viable delivery mechanism for human subjects and subsequently evaluating their in vivo dose–effect dynamics [318]. Overall, the employment of ncRNAs as diagnostic and biomarker molecules in clinical practice requires further wider scale validation and inter-molecule sensitivity and specificity comparisons.

## Conclusion

This study has outlined the major ncRNAs which are dysregulated in AD and which are involved in the regulation of mitophagy, mitochondrial-mediated apoptosis, mitochondrial dynamics and metabolism. While the state of biomarker and therapeutic research in the field of mitochondrially relevant ncRNAs is in its primary stages, the growing body of evidence surrounding the role of ncRNAs in neurodegenerative pathologies makes this a dynamic and open-ended research avenue.

We have discussed the various ncRNAs which have been shown to directly regulate mitochondrial processes in AD. Among mitophagy-relevant ncRNAs which interact with PINK1, miR-140 and lncRNA NEAT1 have the most clinical dysregulation evidence. The miRNAs regulating mitochondrial biogenesis with the best translational research potential are miR-9, miR-34a, miR-146a, miR-155, miR-485, and miR-204. Meanwhile, the best further research candidate in mitochondrial autophagic regulation is miR-106b-5p, as the predicted regulator of AD-relevant autophagic genes has yet to be explored in vitro. Mitochondrially mediated apoptosis is promoted by lncRNA BDNF-AS while miR-148a-3p, miR-21-5p, and miR-103a-3p have antiapoptotic effects, all being dysregulated across various patient tissue types. Mitochondrial dynamics is regulated by several ncRNAs, of which miR-195, miR-124, and miR-455-3p are the most broadly studied in clinical settings as biomarkers, followed by miRNA PC-5P-12969 and miR-137. Finally, experimental and translational evidence points to miR-146a and miR-1273g-3p as regulators of mitochondrial protein import mechanisms in AD.

To conclude, miRNAs are by far the most widely studied class of ncRNAs in relation to mitochondrial dysfunction and biomarker development in AD, followed by lncRNAs. The body of research available mainly focuses on elucidating the multi-faceted mechanistic roles of ncRNAs in mitochondrial homeostasis and on identifying viable diagnostic and monitoring candidates. Although promising, the development of ncRNA therapeutics for AD will require extensive further translationally oriented experimental research.

## Supplementary Information

Below is the link to the electronic supplementary material.

ESM1 (DOCX 76.8 KB)

## Data Availability

No original data were generated in the present study. All data analyzed in this systematic review were extracted from previously published articles cited in the reference list.

## Abbreviations

*3xTg-AD mice*: Triple-transgenic Alzheimer’s disease model mice
*A2M-AS1*: Alpha-2-macroglobulin antisense RNA 1
*ABAT*: 4-Aminobutyrate aminotransferase
*AD*: Alzheimer’s disease
*ADAM10*: A Disintegrin and Metalloproteinase domain 10
*AIF*: Apoptosis-inducing factor
*AKT*: Protein kinase B
*ALS*: Amyotrophic lateral sclerosis
*aMCI*: Aβ-positive amnestic mild cognitive impairment
*AMP*: Adenosine monophosphate
*AMPK*: AMP-activated protein kinase
*APOE*: Apolipoprotein E
*APP*: β-Amyloid precursor protein
*ATP*: Adenosine triphosphate
*AuNPs*: Gold nanoparticles
*Aβ*: Amyloid-beta
*BACE1*: Beta-site amyloid precursor protein cleaving enzyme 1
*BACE1-AS*: BACE1 antisense RNA
*BAD*: Bcl-2-associated death promoter
*BAK*: Bcl-2 homologous antagonist/killer
*BAX*: BCL2-associated X protein
*BCL-2*: B-cell lymphoma 2
*Bcl-2*: B-cell lymphoma 2 protein
*BDNF*: Brain-derived neurotrophic factor
*BDNF-AS*: BDNF antisense RNA
*CAT*: Catalase
*CDK6*: Cyclin-dependent kinase 6
*ceRNA*: Competing endogenous RNA
*circRNA*: Circular RNA
*COX-2*: Cyclooxygenase-2
*CSF*: Cerebrospinal fluid
*DEmiRNA*: Differentially expressed microRNA
*DG*: Dentate gyrus
*Dicer1*: Double-stranded RNA-specific endoribonuclease 1
*DRP1*: Dynamin-related protein 1
*ER*: Endoplasmic reticulum
*EV*: Extracellular vesicles
*FIS1*: Mitochondrial fission protein 1
*GABARAPL1*: GABA type A receptor-associated protein-like 1
*GEO*: Gene Expression Omnibus
*GSK3*: Glycogen synthase kinase 3
*GSK-3β*: Glycogen synthase kinase-3 beta
*GTP*: Guanosine triphosphate
*HCN*: Adult hippocampal neural progenitor (cells)
*HIBCH*: 3-Hydroxyisobutyryl-CoA hydrolase
*hiPSC*: Human induced pluripotent stem cell
*HMGB1*: High mobility group box 1
*hNSC*: Human neural stem cell
*IL-1β*: Interleukin-1 beta
*IMM*: Inner mitochondrial membrane
*iNOS*: Inducible nitric oxide synthase
*iPSC*: Induced pluripotent stem cell
*Klf4*: Krüppel-like factor 4
*KREMEN1*: Kringle-containing transmembrane protein 1
*LC3*: Microtubule-associated proteins 1A/1B light chain 3
*LC3B*: Microtubule-associated protein 1 light chain 3B
*lncMtDloop*: Long non-coding mitochondrial displacement loop RNA
*lncRNA BDNF-AS*: Long non-coding RNA brain-derived neurotrophic factor antisense
*lncRNA NEAT1*: LncRNA nuclear paraspeckle assembly transcript 1
*lncRNA NKILA*: NF-κB-interacting long noncoding RNA
*LncRNA WT1-AS*: Wilms tumor 1 antisense RNA
*lncRNA*: Long non-coding RNA
*LOXL1-AS1*: Lysyl oxidase-like 1 antisense RNA 1
*MAP*: Rush Memory and Aging Project
*MAP1-LC3B*: Microtubule-associated proteins 1A/1B light chain 3B
*mAPP*: Mutant APP
*MCI*: Mild cognitive impairment
*MDA*: Malondialdehyde
*MEG3*: Maternally expressed gene 3
*MFN1*: Mitofusin 1
*MFN2*: Mitofusin 2
*MGME1*: Mitochondrial genome maintenance exonuclease 1
*MIR600HG*: MiR-600 host gene
*miRNA*: MicroRNA
*Miro1*: Mitochondrial Rho GTPase 1
*Miro2*: Mitochondrial Rho GTPase 2
*MMP*: Mitochondrial membrane potential
*MOMP*: Mitochondrial outer membrane permeabilisation
*mRNA*: Messenger RNA
*mtDNA*: Mitochondrial DNA
*mTOR*: Mechanistic target of rapamycin
*mtRNA*: Mitochondrial RNA
*NAD + *: Nicotinamide adenine dinucleotide (oxidized form)
*NADH*: Nicotinamide adenine dinucleotide (reduced form)
*NADPH*: Nicotinamide adenine dinucleotide phosphate
*ncRNA*: Non-coding RNA
*NDAN*: Nondemented patients with Alzheimer’s neuropathology
*NDP52*: Nuclear dot protein 52
*NEDD4L*: Neural precursor cell expressed, developmentally down-regulated 4-like, E3 ubiquitin protein ligase
*NLRP3*: NLR family pyrin domain-containing protein 3
*NRF1*: Nuclear respiratory factor 1
*NRF2*: Nuclear factor erythroid 2-related factor 2
*OPA1*: Optic atrophy 1 gene
*OPTN*: Optineurin
*OSI*: Oxidative stress index
*OXPHOS*: Oxidative phosphorylation
*p62*: Sequestosome-1 (SQSTM1)
*Parkin*: E3 ubiquitin-protein ligase parkin
*PD*: Parkinson’s disease
*PDAD*: Prodromal Alzheimer’s disease
*PDE4A*: Phosphodiesterase 4A
*PFC*: Prefrontal cortex
*PGC-1α*: Peroxisome proliferator-activated receptor-gamma coactivator 1-alpha
*PI3K*: Phosphoinositide 3-kinase
*PINK1*: PTEN-induced putative protein kinase 1
*PRISMA*: Preferred Reporting Items for Systematic Reviews and Meta-Analyses
*PS1*: Presenilin-1
*PSAD*: Presymptomatic Alzheimer’s disease
*PSEN1*: Presenilin 1
*PSEN2*: Presenilin 2
*PTEN*: Phosphatase and tensin homolog
*RAGE*: Receptor for advanced glycation end-products
*RNA*: Ribonucleic acid
*RNA-seq*: RNA sequencing
*ROS*: Reactive oxygen species
*RTN3*: Reticulon 3 gene
*RT-qPCR*: Reverse transcription quantitative polymerase chain reaction
*SAMP8*: Senescence-accelerated mouse prone 8
*siRNA*: Short interfering RNA
*SIRT1*: Sirtuin 1
*SIX4*: SIX homeobox 4
*SNHG1*: Small nucleolar RNA host gene 1
*SOCS6*: Suppressor of cytokine signalling 6
*SOD*: Superoxide dismutase
*STAT3*: Signal transducer and activator of transcription 3
*TBI*: Traumatic brain injury
*TCA*: Tricarboxylic acid cycle
*TFEB*: Transcription factor EB
*TIM*: Translocase of the inner membrane
*TIM23*: Translocase of the inner membrane 23
*TIMM13*: Translocase of the inner mitochondrial membrane 13
*TNF-α*: Tumor necrosis factor alpha
*TOM*: Translocase of the outer membrane
*TOMM*: Translocase of the outer mitochondrial membrane (complex)
*TOMM20*: Translocase of the outer mitochondrial membrane 20
*TRPML1*: Transient receptor potential mucolipin-1
*UBE2A*: Ubiquitin-conjugating enzyme E2A
*UTR*: Untranslated region
*VDAC1*: Voltage-dependent anion channel 1
*WT*: Wild-type
*WT1*: Wilms tumor 1 gene
*WT1-AS*: Wilms tumor 1 antisense RNA
*ZNF217*: Zinc finger protein 217
*MDH2*: Malate dehydrogenase 2
*NDUFB10*: NADH:ubiquinone oxidoreductase subunit B10
*NDUFS1*: NADH:ubiquinone oxidoreductase core subunit S1
*NDUFS2*: NADH:ubiquinone oxidoreductase core subunit S2
*NDUFS3*: NADH:ubiquinone oxidoreductase core subunit S3
*NDUFS8*: NADH:ubiquinone oxidoreductase core subunit S8
*NDUFV1*: NADH:ubiquinone oxidoreductase core subunit V1
*PDHA1*: Pyruvate dehydrogenase E1 subunit alpha 1
*SDHB*: Succinate dehydrogenase complex iron sulfur subunit B
*UQCRFS1*: Ubiquinol-cytochrome c reductase Rieske iron-sulfur polypeptide 1
*Poly(A) RNA-seq*: Polyadenylated RNA sequencing

## References

[1] Amiri F, Safiri S, Shamekh A, Ebrahimi A, Sullman MJM, Kolahi A-A (2025) Prevalence, deaths and disability-adjusted life years due to Alzheimer’s disease and other dementias in Middle East and North Africa, 1990–2021. Sci Rep 15:7058. 10.1038/s41598-025-89899-w 40016362 PMC11868542

[2] Baviskar PS, Mahajan HS (2026) Unveiling Alzheimer’s disease (1901–2025): historical insights, global burden, biological mechanisms, diagnostics, and therapeutic strategies. Ageing Res Rev 114:102990. 10.1016/j.arr.2025.102990 41386339

[3] Savelieff MG, Nam G, Kang J, Lee HJ, Lee M, Lim MH (2019) Development of multifunctional molecules as potential therapeutic candidates for Alzheimer’s disease, Parkinson’s disease, and amyotrophic lateral sclerosis in the last decade. Chem Rev 119:1221–1322. 10.1021/acs.chemrev.8b00138 30095897

[4] Monteiro AR, Barbosa DJ, Remião F, Silva R (2023) Alzheimer’s disease: insights and new prospects in disease pathophysiology, biomarkers and disease-modifying drugs. Biochem Pharmacol 211:115522. 10.1016/j.bcp.2023.115522 36996971

[5] Hampel H, Hardy J, Blennow K, Chen C, Perry G, Kim SH, Villemagne VL, Aisen P, et al. (2021) The Amyloid-β pathway in Alzheimer’s disease. Mol Psychiatry 26:5481–5503. 10.1038/s41380-021-01249-0 34456336 PMC8758495

[6] Nanclares C, Colmena I, Muñoz-Montero A, Baraibar AM, De Pascual R, Wojnicz A, Ruiz-Nuño A, García AG, et al. (2025) Beyond the brain: early autonomic dysfunction in Alzheimer’s disease. Acta Neuropathol Commun 13:128. 10.1186/s40478-025-02042-8 40495232 PMC12150586

[7] Ionescu-Tucker A, Cotman CW (2021) Emerging roles of oxidative stress in brain aging and Alzheimer’s disease. Neurobiol Aging 107:86–95. 10.1016/j.neurobiolaging.2021.07.014 34416493

[8] Leng F, Edison P (2021) Neuroinflammation and microglial activation in Alzheimer disease: where do we go from here? Nat Rev Neurol 17:157–172. 10.1038/s41582-020-00435-y 33318676

[9] Karch CM, Goate AM (2015) Alzheimer’s disease risk genes and mechanisms of disease pathogenesis. Biol Psychiatry 77:43–51. 10.1016/j.biopsych.2014.05.006 24951455 PMC4234692

[10] Stern A, Linial M (2025) Integrative machine learning approach to risk prediction for dementia and Alzheimer’s disease. GeroScience 48:3007–3023. 10.1007/s11357-025-01828-x 40864401 PMC12972432

[11] Tahami Monfared AA, Byrnes MJ, White LA, Zhang Q (2022) Alzheimer’s disease: epidemiology and clinical progression. Neurol Ther 11:553–569. 10.1007/s40120-022-00338-8 35286590 PMC9095793

[12] Poliseno L, Lanza M, Pandolfi PP (2024) Coding, or non-coding, that is the question. Cell Res 34:609–629. 10.1038/s41422-024-00975-8 39054345 PMC11369213

[13] Chaabane M, Williams RM, Stephens AT, Park JW (2020) circDeep: deep learning approach for circular RNA classification from other long non-coding RNA. Bioinformatics 36:73–80. 10.1093/bioinformatics/btz537 31268128 PMC6956777

[14] Laurent GS, Wahlestedt C, Kapranov P (2015) The landscape of long noncoding RNA classification. Trends Genet 31:239–251. 10.1016/j.tig.2015.03.007 25869999 PMC4417002

[15] Mattick JS, Amaral PP, Carninci P, Carpenter S, Chang HY, Chen L-L, Chen R, Dean C, et al. (2023) Long non-coding RNAs: definitions, functions, challenges and recommendations. Nat Rev Mol Cell Biol 24:430–447. 10.1038/s41580-022-00566-8 36596869 PMC10213152

[16] Mercer TR, Dinger ME, Mattick JS (2009) Long non-coding RNAs: insights into functions. Nat Rev Genet 10:155–159. 10.1038/nrg2521 19188922

[17] Wang KC, Chang HY (2011) Molecular mechanisms of long noncoding RNAs. Mol Cell 43:904–914. 10.1016/j.molcel.2011.08.018 21925379 PMC3199020

[18] Chen L, Wang C, Sun H, Wang J, Liang Y, Wang Y, Wong G (2020) The bioinformatics toolbox for circRNA discovery and analysis. Brief Bioinform 22:1706–1728. 10.1093/bib/bbaa001 PMC7986655 32103237

[19] Chodurska B, Kunej T (2025) Long non-coding RNAs in humans: classification, genomic organization and function. Non-Coding RNA Res 11:313–327. 10.1016/j.ncrna.2025.01.004 PMC11833636 39967600

[20] Martens JA, Laprade L, Winston F (2004) Intergenic transcription is required to repress the *Saccharomyces cerevisiae* SER3 gene. Nature 429:571–574. 10.1038/nature02538 15175754

[21] Hirota K, Miyoshi T, Kugou K, Hoffman CS, Shibata T, Ohta K (2008) Stepwise chromatin remodelling by a cascade of transcription initiation of non-coding RNAs. Nature 456:130–134. 10.1038/nature07348 18820678

[22] Ponting CP, Oliver PL, Reik W (2009) Evolution and functions of long noncoding RNAs. Cell 136:629–641. 10.1016/j.cell.2009.02.006 19239885

[23] Saliminejad K, Khorram Khorshid HR, Soleymani Fard S, Ghaffari SH (2019) An overview of microRNAs: biology, functions, therapeutics, and analysis methods. J Cell Physiol 234:5451–5465. 10.1002/jcp.27486 30471116

[24] Meister G, Landthaler M, Patkaniowska A, Dorsett Y, Teng G, Tuschl T (2004) Human Argonaute2 mediates RNA cleavage targeted by miRNAs and siRNAs. Mol Cell 15:185–197. 10.1016/j.molcel.2004.07.007 15260970

[25] Wilusz JE, Sharp PA (2013) A circuitous route to noncoding RNA. Science 340:440–441. 10.1126/science.1238522 23620042 PMC4063205

[26] Lan Z, Chen Y, Jin J, Xu Y, Zhu X (2022) Long non-coding RNA: insight into mechanisms of Alzheimer’s disease. Front Mol Neurosci 14:821002. 10.3389/fnmol.2021.821002 35095418 PMC8795976

[27] Ghafouri-Fard S, Shoorei H, Taheri M (2020) Non-coding RNAs are involved in the response to oxidative stress. Biomed Pharmacother 127:110228. 10.1016/j.biopha.2020.110228 32559852

[28] Olufunmilayo EO, Holsinger RMD (2023) Roles of non-coding RNA in Alzheimer’s disease pathophysiology. Int J Mol Sci 24:12498. 10.3390/ijms241512498 37569871 PMC10420049

[29] Adiga D, Eswaran S, Srinath S, Khan NG, Kumar D, Kabekkodu SP (2024) Noncoding RNAs in Alzheimer’s disease: overview of functional and therapeutic significance. Curr Top Med Chem 24:1615–1634. 10.2174/0115680266293212240405042540 38616763

[30] Liu Y, Cheng X, Li H, Hui S, Zhang Z, Xiao Y, Peng W (2022) Non-coding RNAs as novel regulators of neuroinflammation in Alzheimer’s disease. Front Immunol 13:908076. 10.3389/fimmu.2022.908076 35720333 PMC9201920

[31] Singh S, Singh RK (2025) Recent advancements in the understanding of the alterations in mitochondrial biogenesis in Alzheimer’s disease. Mol Biol Rep 52:173. 10.1007/s11033-025-10297-6 39880979

[32] Klemmensen MM, Borrowman SH, Pearce C, Pyles B, Chandra B (2024) Mitochondrial dysfunction in neurodegenerative disorders. Neurotherapeutics 21:e00292. 10.1016/j.neurot.2023.10.002 38241161 PMC10903104

[33] Bustamante-Barrientos FA, Luque-Campos N, Araya MJ, Lara-Barba E, De Solminihac J, Pradenas C, Molina L, Herrera-Luna Y, et al. (2023) Mitochondrial dysfunction in neurodegenerative disorders: potential therapeutic application of mitochondrial transfer to central nervous system-residing cells. J Transl Med 21:613. 10.1186/s12967-023-04493-w 37689642 PMC10493034

[34] Chen L, Guo X, Li Z, He Y (2019) Relationship between long non-coding RNAs and Alzheimer’s disease: a systematic review. Pathol Res Pract 215:12–20. 10.1016/j.prp.2018.11.012 30470438

[35] Tan L, Yu J-T, Hu N, Tan L (2013) Non-coding RNAs in Alzheimer’s disease. Mol Neurobiol 47:382–393. 10.1007/s12035-012-8359-5 23054683

[36] Shobeiri P, Alilou S, Jaberinezhad M, Zare F, Karimi N, Maleki S, Teixeira AL, Perry G, et al. (2023) Circulating long non-coding RNAs as novel diagnostic biomarkers for Alzheimer’s disease (AD): a systematic review and meta-analysis. PLoS One 18:e0281784. 10.1371/journal.pone.0281784 36947499 PMC10032479

[37] Canoy RJ, Sy JC, Deguit CD, Castro CB, Dimaapi LJ, Panlaqui BG, Perian W, Yu J, et al. (2024) Non-coding RNAs involved in the molecular pathology of Alzheimer’s disease: a systematic review. Front Neurosci. 10.3389/fnins.2024.1421675 39005845 PMC11243705

[38] Abed S, Ebrahimi A, Fattahi F, Kouchakali G, Shekari-Khaniani M, Mansoori-Derakhshan S (2024) The role of non-coding RNAs in mitochondrial dysfunction of Alzheimer’s disease. J Mol Neurosci 74:100. 10.1007/s12031-024-02262-y 39466447

[39] Rivera J, Gangwani L, Kumar S (2023) Mitochondria localized microRNAs: an unexplored miRNA niche in Alzheimer’s disease and aging. Cells (Basel) 12:742. 10.3390/cells12050742 PMC10000969 36899879

[40] Subasinghe K, Barber R, Phillips N (2025) miRNA mediated mitochondrial function and gene regulation associated with Alzheimer’s disease. Front Aging 6:1582812. 10.3389/fragi.2025.1582812 40433169 PMC12106540

[41] Gowda P, Reddy PH, Kumar S (2022) Deregulated mitochondrial microRNAs in Alzheimer’s disease: focus on synapse and mitochondria. Ageing Res Rev 73:101529. 10.1016/j.arr.2021.101529 34813976 PMC8692431

[42] Adak S, Singh S, Jain R, Tiwari A, Singh B, Kumar S, Dadhwal A, Chakroborty A, et al. (2025) Advancement in modeling of Alzheimer’s disease: a comprehensive review of preclinical screening platforms. Front Aging Neurosci. 10.3389/fnagi.2025.1646551 40842652 PMC12364833

[43] Page MJ, McKenzie JE, Bossuyt PM, Boutron I, Hoffmann TC, Mulrow CD, Shamseer L, Tetzlaff JM et al (2021) The PRISMA 2020 statement: an updated guideline for reporting systematic reviews. BMJ. 10.1136/bmj.n71 33782057 PMC8005924

[44] Ouzzani M, Hammady H, Fedorowicz Z, Elmagarmid A (2016) Rayyan—a web and mobile app for systematic reviews. Syst Rev 5:210. 10.1186/s13643-016-0384-4 27919275 PMC5139140

[45] Wang H, Lu B, Chen J (2019) Knockdown of lncRNA SNHG1 attenuated Aβ25-35-inudced neuronal injury via regulating KREMEN1 by acting as a ceRNA of miR-137 in neuronal cells. Biochem Biophys Res Commun 518:438–444. 10.1016/j.bbrc.2019.08.033 31447119

[46] Wang Q, Ge X, Zhang J, Chen L (2020) Effect of lncRNA WT1-AS regulating WT1 on oxidative stress injury and apoptosis of neurons in Alzheimer’s disease via inhibition of the miR-375/SIX4 axis. Aging (Albany NY) 12:23974–23995. 10.18632/aging.104079 33234729 PMC7762490

[47] Zhang Y-Y, Bao H-L, Dong L-X, Liu Y, Zhang G-W, An F-M (2021) Silenced lncRNA H19 and up-regulated microRNA-129 accelerates viability and restrains apoptosis of PC12 cells induced by Aβ(25–35) in a cellular model of Alzheimer’s disease. Cell Cycle Georget Tex 20:112–125. 10.1080/15384101.2020.1863681 PMC7849745 33410377

[48] Chen J, Xiao S, Cui X, Gao X, Wang D, Li X, Qi W, Wang B (2025) Circ_0049472 downregulation relieves Amyloid-β-induced neuronal injury by modulating PDE4A expression via targeting miR-22-3p in Alzheimer’s disease. Metab Brain Dis 40:252. 10.1007/s11011-025-01652-4 40879806

[49] Corsi GI, Gadekar VP, Haukedal H, Doncheva NT, Anthon C, Ambardar S, Palakodeti D, Hyttel P, et al. (2023) The transcriptomic landscape of neurons carrying PSEN1 mutations reveals changes in extracellular matrix components and non-coding gene expression. Neurobiol Dis 178:105980. 10.1016/j.nbd.2022.105980 36572121

[50] Wang Y, Lian M, Zhou J, Wu S (2020) Brain Dicer1 is down-regulated in a mouse model of Alzheimer’s disease via Aβ42-induced repression of nuclear factor erythroid 2-related factor 2. Mol Neurobiol 57:4417–4437. 10.1007/s12035-020-02036-8 32737764

[51] Yuen SC, Lee SM-Y, Leung S (2022) Putative factors interfering cell cycle re-entry in Alzheimer’s disease: an omics study with differential expression meta-analytics and co-expression profiling. J Alzheimers Dis 85:1373–1398. 10.3233/JAD-215349 34924393

[52] Xu X, Fan S, Li J, Wu H, He J, He Y, Meng X, Shen Y (2025) Integrated multi-omics analysis and cross-model validation reveal mitochondrial signatures in Alzheimer’s disease. CNS Neurosci Ther 31:e70634. 10.1111/cns.70634 41146472 PMC12559030

[53] Li H, Feng F, Xie S, Ma Y, Wang Y, Zhang F, Wu H, Huang S (2025) Identification of HIBCH and MGME1 as mitochondrial dynamics-related biomarkers in Alzheimer’s disease via integrated bioinformatics analysis. IET Syst Biol 19:e70018. 10.1049/syb2.70018 40286336 PMC12033025

[54] Abyadeh M, Kaya A (2025) Multiomics from Alzheimer’s brains and mesenchymal stem cell-derived extracellular vesicles identifies therapeutic potential of specific subpopulations to target mitochondrial proteostasis. J Cent Nerv Syst Dis 17:11795735251336302. 10.1177/11795735251336302 40297324 PMC12035200

[55] Fracassi A, Marcatti M, Zolochevska O, Tabor N, Woltjer R, Moreno S, Taglialatela G (2021) Oxidative damage and antioxidant response in frontal cortex of demented and nondemented individuals with Alzheimer’s neuropathology. J Neurosci 41:538–554. 10.1523/JNEUROSCI.0295-20.2020 33239403 PMC7821866

[56] Nguyen HD, Kim W-K, Huong Vu G (2024) Molecular mechanisms implicated in protein changes in the Alzheimer’s disease human hippocampus. Mech Ageing Dev 219:111930. 10.1016/j.mad.2024.111930 38554950

[57] Cieślik M, Czapski GA, Wójtowicz S, Wieczorek I, Wencel PL, Strosznajder RP, Jaber V, Lukiw WJ, et al. (2020) Alterations of transcription of genes coding anti-oxidative and mitochondria-related proteins in amyloid β toxicity: relevance to Alzheimer’s disease. Mol Neurobiol 57:1374–1388. 10.1007/s12035-019-01819-y 31734880 PMC7061023

[58] Kim SH, Choi KY, Park Y, McLean C, Park J, Lee JH, Lee K-H, Kim BC, et al. (2021) Enhanced expression of microRNA-1273g-3p contributes to Alzheimer’s disease pathogenesis by regulating the expression of mitochondrial genes. Cells 10:2697. 10.3390/cells10102697 34685681 PMC8534383

[59] Kim SJ, Russell AE, Wang W, Gemoets DE, Sarkar SN, Simpkins JW, Brown CM (2022) MiR-146a dysregulates energy metabolism during neuroinflammation. J Neuroimmune Pharmacol 17:228–241. 10.1007/s11481-021-09999-y 34028667 PMC8611101

[60] Huang H-Z, Ai W-Q, Wei N, Zhu L-S, Liu Z-Q, Zhou C-W, Deng M-F, Zhang W-T, et al. (2024) Senktide blocks aberrant RTN3 interactome to retard memory decline and tau pathology in social isolated Alzheimer’s disease mice. Protein Cell 15:261–284. 10.1093/procel/pwad056 38011644 PMC10984625

[61] Han AR, Moon TK, Kang IK, Yu DB, Kim Y, Byon C, Park S, Kim HL, et al. (2024) Integrative analysis of microRNA-mediated mitochondrial dysfunction in hippocampal neural progenitor cell death in relation with Alzheimer’s disease. BMB Rep 57:281–286. 10.5483/BMBRep.2023-0167 38053296 PMC11214893

[62] Xu W, Su X, Qin J, Jin Y, Zhang N, Huang S (2024) Identification of autophagy-related biomarkers and diagnostic model in Alzheimer’s disease. Genes 15:1027. 10.3390/genes15081027 39202387 PMC11354206

[63] Woo H-N, Park S, Kim HL, Jung M-K, Pack C-G, Park J, Cho Y, Jo D-G, et al. (2021) MiR-351-5p/Miro2 axis contributes to hippocampal neural progenitor cell death via unbalanced mitochondrial fission. Mol Ther Nucleic Acids 23:643–656. 10.1016/j.omtn.2020.12.014 33575111 PMC7848773

[64] Vijayan M, Reddy PH (2024) Unveiling the role of novel miRNA PC-5P-12969 in alleviating Alzheimer’s disease. J Alzheimers Dis 98:1329–1348. 10.3233/JAD-231281 38552115

[65] Ramesh M, Govindaraju T (2025) MiR-7a–Klf4 axis as a regulator and therapeutic target of neuroinflammation and ferroptosis in Alzheimer’s disease. NAR Mol Med 2:ugaf022. 10.1093/narmme/ugaf022 41256289 PMC12429957

[66] Wang Y, Sun X, He B, Yu S (2025) Ginsenoside Rg1 downregulates miR-9-5p expression to modulate SIRT1-mediated mitochondrial dysfunction and ameliorate Alzheimer’s disease. Mol Neurobiol 62:13044–13059. 10.1007/s12035-025-05073-3 40478516

[67] Rao S, Madhu LN, Babu RS, Shankar G, Kotian S, Nagarajan A, Upadhya R, Narvekar E, et al. (2025) Extracellular vesicles from hiPSC-derived NSCs protect human neurons against Aβ-42 oligomers induced neurodegeneration, mitochondrial dysfunction and tau phosphorylation. Stem Cell Res Ther 16:191. 10.1186/s13287-025-04324-3 40251643 PMC12008877

[68] Liang C, Mu Y, Tian H, Wang D, Zhang S, Wang H, Liu Y, Di C (2021) MicroRNA-140 silencing represses the incidence of Alzheimer’s disease. Neurosci Lett 758:135674. 10.1016/j.neulet.2021.135674 33529652

[69] Liu J, Zuo X, Han J, Dai Q, Xu H, Liu Y, Cui S (2020) MiR-9-5p inhibits mitochondrial damage and oxidative stress in AD cell models by targeting GSK-3β. Biosci Biotechnol Biochem 84:2273–2280. 10.1080/09168451.2020.1797469 32713252

[70] Zhang R, Zhou H, Jiang L, Mao Y, Cui X, Xie B, Cui D, Wang H, et al. (2016) MiR-195 dependent roles of mitofusin2 in the mitochondrial dysfunction of hippocampal neurons in SAMP8 mice. Brain Res 1652:135–143. 10.1016/j.brainres.2016.09.047 27693395

[71] Zhang L, Fang Y, Zhao X, Zheng Y, Ma Y, Li S, Huang Z, Li L (2021) MiR-204 silencing reduces mitochondrial autophagy and ROS production in a murine AD model via the TRPML1-activated STAT3 pathway. Mol Ther - Nucleic Acids 24:822–831. 10.1016/j.omtn.2021.02.010 34026326 PMC8121631

[72] Garcia G, Pinto S, Cunha M, Fernandes A, Koistinaho J, Brites D (2021) Neuronal dynamics and miRNA signaling differ between SH-SY5Y APPSwe and PSEN1 mutant iPSC-derived AD models upon modulation with miR-124 mimic and inhibitor. Cells 10:2424. 10.3390/cells10092424 34572073 PMC8465877

[73] Garcia G, Fernandes A, Stein F, Brites D (2022) Protective signature of IFNγ-stimulated microglia relies on miR-124-3p regulation from the secretome released by mutant APP Swedish neuronal cells. Front Pharmacol 13:833066. 10.3389/fphar.2022.833066 35620289 PMC9127204

[74] Kumar S, Reddy AP, Yin X, Reddy PH (2019) Novel MicroRNA-455-3p and its protective effects against abnormal APP processing and amyloid beta toxicity in Alzheimer’s disease. Biochim Biophys Acta BBA - Mol Basis Dis 1865:2428–2440. 10.1016/j.bbadis.2019.06.006 PMC6688505 31181293

[75] Xiong W, Xu K, Sun JK-L, Liu S, Zhao B, Shi J, Herrup K, Chow H-M, et al. (2024) The mitochondrial long non-coding RNA lncMtloop regulates mitochondrial transcription and suppresses Alzheimer’s disease. EMBO J 43:6001–6031. 10.1038/s44318-024-00270-7 39424953 PMC11612450

[76] Wang J, Zhou T, Wang T, Wang B (2018) Suppression of lncRNA-ATB prevents amyloid-β-induced neurotoxicity in PC12 cells via regulating miR-200/ZNF217 axis. Biomed Pharmacother 108:707–715. 10.1016/j.biopha.2018.08.155 30248538

[77] Liu Q, Ling Z, Zhang J, Yu H, Wang Y, Xue Y, Wang C, Zhao J, et al. (2022) LncRNA MIR600HG knockdown alleviates cognitive impairment in Alzheimer’s disease through NEDD4L mediated PINK1 degradation. J Alzheimers Dis 85:1783–1794. 10.3233/JAD-215194 34958029

[78] Huang Z, Zhao J, Wang W, Zhou J, Zhang J (2020) Depletion of LncRNA NEAT1 rescues mitochondrial dysfunction through NEDD4L-dependent PINK1 degradation in animal models of Alzheimer’s disease. Front Cell Neurosci 14:28. 10.3389/fncel.2020.00028 32140098 PMC7043103

[79] Guo C-C, Jiao C, Gao Z-M (2018) Silencing of LncRNA BDNF-AS attenuates Aβ_25-35_ -induced neurotoxicity in PC12 cells by suppressing cell apoptosis and oxidative stress. Neurol Res 40:795–804. 10.1080/01616412.2018.1480921 29902125

[80] Li N, Zhang D, Guo H, Yang Q, Li P, He Y (2022) Inhibition of circ_0004381 improves cognitive function via miR-647/PSEN1 axis in an Alzheimer disease mouse model. J Neuropathol Exp Neurol 82:84–92. 10.1093/jnen/nlac108 36409993

[81] Wang G, Shen X, Song X, Wang N, Wo X, Gao Y (2023) Protective mechanism of gold nanoparticles on human neural stem cells injured by β-amyloid protein through miR-21–5p/SOCS6 pathway. Neurotoxicology 95:12–22. 10.1016/j.neuro.2022.12.011 36623431

[82] Wang Y, Xiao Z, Yin H, Ren Z, Ma X, Wang Y, Zhang Y, Fu X, et al. (2024) MiRNA375-3p/rapamycin mediates the mTOR pathway by decreasing PS1, enhances microglial cell activity to regulate autophagy in Alzheimer’s disease. Heliyon. 10.1016/j.heliyon.2024.e37589 39386837 PMC11461998

[83] Tan MS, Cheah P-L, Chin A-V, Looi L-M, Chang S-W (2023) Differential expression analysis of blood microRNA in identifying potential genes relevant to Alzheimer’s disease pathogenesis, using an integrated bioinformatics and machine learning approach. Appl Sci (Basel) 13:3071. 10.3390/app13053071

[84] Cogswell JP, Ward J, Taylor IA, Waters M, Shi Y, Cannon B, Kelnar K, Kemppainen J, et al. (2008) Identification of miRNA changes in Alzheimer’s disease brain and CSF yields putative biomarkers and insights into disease pathways. J Alzheimers Dis 14:27–41. 10.3233/JAD-2008-14103 18525125

[85] Li W, Dong M-J, Dai H, Lu S, Luo R, Cao J, Zhang F, Mei L, et al. (2025) Application of mitochondrial miRNA-204 nanoprobes in Alzheimer’s disease treatment by clearing reactive oxygen species-mediated autophagy. Chin Chem Lett 36:110614. 10.1016/j.cclet.2024.110614

[86] Wu B-W, Guo J-D, Wu M-S, Liu Y, Lu M, Zhou Y-H, Han H-W (2020) Osteoblast-derived lipocalin-2 regulated by miRNA-96-5p/Foxo1 advances the progression of Alzheimer’s disease. Epigenomics 12:1501–1513. 10.2217/epi-2019-0215 32901506

[87] Ashrafi G, Schwarz TL (2013) The pathways of mitophagy for quality control and clearance of mitochondria. Cell Death Differ 20:31–42. 10.1038/cdd.2012.81 22743996 PMC3524633

[88] Onishi M, Yamano K, Sato M, Matsuda N, Okamoto K (2021) Molecular mechanisms and physiological functions of mitophagy. EMBO J 40:e104705. 10.15252/embj.2020104705 33438778 PMC7849173

[89] Nguyen TN, Padman BS, Usher J, Oorschot V, Ramm G, Lazarou M (2016) Atg8 family LC3/GABARAP proteins are crucial for autophagosome–lysosome fusion but not autophagosome formation during PINK1/Parkin mitophagy and starvation. J Cell Biol 215:857–874. 10.1083/jcb.201607039 27864321 PMC5166504

[90] Akhter R, Shao Y, Shaw M, Formica S, Khrestian M, Leverenz JB, Bekris LM (2018) Regulation of ADAM10 by miR-140-5p and potential relevance for Alzheimer’s disease. Neurobiol Aging 63:110–119. 10.1016/j.neurobiolaging.2017.11.007 29253717 PMC5800962

[91] Song C, Li S, Mai Y, Li L, Dai G, Zhou Y, Liang X, Zou OM, et al. (2024) Dysregulated expression of miR-140 and miR-122 compromised microglial chemotaxis and led to reduced restriction of AD pathology. J Neuroinflammation 21:167. 10.1186/s12974-024-03162-z 38956605 PMC11218311

[92] Li J, Li Y, Jiao J, Wang J, Li Y, Qin D, Li P (2014) Mitofusin 1 is negatively regulated by microRNA 140 in cardiomyocyte apoptosis. Mol Cell Biol 34:1788–1799. 10.1128/MCB.00774-13 24615014 PMC4019028

[93] Yang S, Li H, Chen L (2019) MicroRNA-140 attenuates myocardial ischemia-reperfusion injury through suppressing mitochondria-mediated apoptosis by targeting YES1. J Cell Biochem 120:3813–3821. 10.1002/jcb.27663 30259997

[94] Zhang Q, Bian ZX, Song Y, Wang X, Zhang H, Ren Q, Chen S (2022) Regulation of mitophagy through HIF-1α/miR-140-5p/PARKIN axis in acute kidney injury. Environ Toxicol 37:1759–1767. 10.1002/tox.23523 35312153

[95] Shreya SG, Hunjan G, Aran KR (2026) Role of LncRNA NEAT1 in Alzheimer’s disease: pathophysiological insights and therapeutic approaches. Neurotox Res 44:13. 10.1007/s12640-026-00792-4 41863580

[96] Li Y, Fan H, Ni M, Zhang W, Fang F, Sun J, Lyu P, Ma P (2023) Targeting lncRNA NEAT1 hampers Alzheimer’s disease progression. Neuroscience 529:88–98. 10.1016/j.neuroscience.2023.02.016 37286157

[97] Yan W, Chen Z-Y, Chen J-Q, Chen H-M (2018) LncRNA NEAT1 promotes autophagy in MPTP-induced Parkinson’s disease through stabilizing PINK1 protein. Biochem Biophys Res Commun 496:1019–1024. 10.1016/j.bbrc.2017.12.149 29287722

[98] Lee D-E, Yoo JE, Kim J, Kim S, Kim S, Lee H, Cheong H (2020) NEDD4L downregulates autophagy and cell growth by modulating ULK1 and a glutamine transporter. Cell Death Dis 11:38. 10.1038/s41419-020-2242-5 31959741 PMC6971022

[99] Han F, Wu S, Dong Y, Liu Y, Sun B, Chen L (2024) Aberrant expression of NEDD4L disrupts mitochondrial homeostasis by downregulating CaMKKβ in diabetic kidney disease. J Transl Med 22:465. 10.1186/s12967-024-05207-6 38755664 PMC11100153

[100] Jiang B, Bai F, Hu Y, Ren Y, Su Y, Song W, Xie K, Wang D, et al. (2025) Endothelial major vault protein alleviates vascular remodeling via promoting Parkin-mediated mitophagy. Nat Commun 16:4365. 10.1038/s41467-025-59644-y 40348769 PMC12065841

[101] Wang Z, Zhou J, Zhang B, Xu Z, Wang H, Sun Q, Wang N (2024) Inhibitory effects of β-asarone on lncRNA BACE1-mediated induction of autophagy in a model of Alzheimer’s disease. Behav Brain Res 463:114896. 10.1016/j.bbr.2024.114896 38316166

[102] Zhang W, Zhao H, Wu Q, Xu W, Xia M (2018) Knockdown of BACE1-AS by siRNA improves memory and learning behaviors in Alzheimer’s disease animal model. Exp Ther Med 16:2080–2086. 10.3892/etm.2018.6359 30186443 PMC6122303

[103] Talebi Taheri A, Golshadi Z, Zare H, Alinaghipour A, Faghihi Z, Dadgostar E, Tamtaji Z, Aschner M, et al. (2024) The potential of targeting autophagy-related non-coding RNAs in the treatment of Alzheimer’s and Parkinson’s diseases. Cell Mol Neurobiol 44:28. 10.1007/s10571-024-01461-w 38461204 PMC10924707

[104] Kayano M, Higaki S, Satoh J, Matsumoto K, Matsubara E, Takikawa O, Niida S (2016) Plasma microRNA biomarker detection for mild cognitive impairment using differential correlation analysis. Biomark Res 4:22. 10.1186/s40364-016-0076-1 27999671 PMC5151129

[105] Toury L, Frankel D, Airault C, Magdinier F, Roll P, Kaspi E (2022) MiR-140-5p and miR-140-3p: key actors in aging-related diseases? Int J Mol Sci 23:11439. 10.3390/ijms231911439 36232738 PMC9570089

[106] Fotuhi SN, Khalaj-Kondori M, Hoseinpour Feizi MA, Talebi M (2019) Long non-coding RNA BACE1-AS may serve as an Alzheimer’s disease blood-based biomarker. J Mol Neurosci 69:351–359. 10.1007/s12031-019-01364-2 31264051

[107] Feng L, Liao Y-T, He J-C, Xie C-L, Chen S-Y, Fan H-H, Su Z-P, Wang Z (2018) Plasma long non-coding RNA BACE1 as a novel biomarker for diagnosis of Alzheimer disease. BMC Neurol 18:4. 10.1186/s12883-017-1008-x 29316899 PMC5761117

[108] Zhou Y, Ge Y, Liu Q, Li Y-X, Chao X, Guan J-J, Diwu Y-C, Zhang Q (2021) LncRNA BACE1-AS promotes autophagy-mediated neuronal damage through the miR-214-3p/ATG5 signalling axis in Alzheimer’s disease. Neuroscience 455:52–64. 10.1016/j.neuroscience.2020.10.028 33197504

[109] Khodayi M, Khalaj-Kondori M, Hoseinpour Feizi MA, Jabarpour Bonyadi M, Talebi M (2022) Plasma lncRNA profiling identified BC200 and NEAT1 lncRNAs as potential blood-based biomarkers for late-onset Alzheimer’s disease. EXCLI J 21:772–785. 10.17179/excli2022-4764 PMC9360476 35949493

[110] Boros FA, Maszlag-Török R, Vécsei L, Klivényi P (2020) Increased level of NEAT1 long non-coding RNA is detectable in peripheral blood cells of patients with Parkinson’s disease. Brain Res 1730:146672. 10.1016/j.brainres.2020.146672 31953211

[111] Jiang M, Wang Z, Lu T, Li X, Yang K, Zhao L, Zhang D, Li J, et al. (2023) Integrative analysis of long noncoding RNAs dysregulation and synapse-associated ceRNA regulatory axes in autism. Transl Psychiatry 13:375. 10.1038/s41398-023-02662-5 38057311 PMC10700319

[112] Yang J, Zhang W, Zhang S, Iyaswamy A, Sun J, Wang J, Yang C (2023) Novel insight into functions of transcription factor EB (TFEB) in Alzheimer’s disease and Parkinson’s disease. Aging Dis 14:652–669. 10.14336/AD.2022.0927 37191408 PMC10187700

[113] Kumari S, Dhapola R, Reddy DH (2023) Apoptosis in Alzheimer’s disease: insight into the signaling pathways and therapeutic avenues. Apoptosis 28:943–957. 10.1007/s10495-023-01848-y 37186274

[114] Zhang L, Fang J, Tang Z, Luo Y (2022) A bioinformatics perspective on the dysregulation of ferroptosis and ferroptosis-related immune cell infiltration in Alzheimer’s disease. Int J Med Sci 19:1888–1902. 10.7150/ijms.76660 36438927 PMC9682502

[115] Bagci EZ, Vodovotz Y, Billiar TR, Ermentrout GB, Bahar I (2006) Bistability in apoptosis: roles of Bax, Bcl-2, and mitochondrial permeability transition pores. Biophys J 90:1546–1559. 10.1529/biophysj.105.068122 16339882 PMC1367306

[116] Lopez J, Tait SWG (2015) Mitochondrial apoptosis: killing cancer using the enemy within. Br J Cancer 112:957–962. 10.1038/bjc.2015.85 25742467 PMC4366906

[117] Brunelle JK, Letai A (2009) Control of mitochondrial apoptosis by the Bcl-2 family. J Cell Sci 122:437–441. 10.1242/jcs.031682 19193868 PMC2714431

[118] Stiles BL (2009) PI-3-K and AKT: onto the mitochondria☆. Adv Drug Deliv Rev 61:1276–1282. 10.1016/j.addr.2009.07.017 19720099

[119] Ding Y, Luan W, Shen X, Wang Z, Cao Y (2022) LncRNA BDNF-AS as ceRNA regulates the miR-9-5p/BACE1 pathway affecting neurotoxicity in Alzheimer’s disease. Arch Gerontol Geriatr 99:104614. 10.1016/j.archger.2021.104614 34990931

[120] Soman SK, Swain M, Dagda RK (2025) BDNF-TrkB signaling in mitochondria: implications for neurodegenerative diseases. Mol Neurobiol 62:1756–1769. 10.1007/s12035-024-04357-4 39030441 PMC11909598

[121] Ren H, Qiu W, Zhu B, Li Q, Peng C, Chen X (2023) The long non-coding RNA BDNF-AS induces neuronal cell apoptosis by targeting miR-125b-5p in Alzheimer’s disease models. Adv Clin Exp Med 33:233–245. 10.17219/acem/168241 37486697

[122] Jovicic A, Zaldivar Jolissaint JF, Moser R, Silva Santos M, Luthi-Carter R (2013) MicroRNA-22 (miR-22) overexpression is neuroprotective via general anti-apoptotic effects and may also target specific Huntington’s disease-related mechanisms. PLoS One 8:e54222. 10.1371/journal.pone.0054222 23349832 PMC3547907

[123] Zeng L, Jiang H, Ashraf GM, Liu J, Wang L, Zhao K, Liu M, Li Z, et al. (2021) Implications of miR-148a-3p/p35/PTEN signaling in tau hyperphosphorylation and autoregulatory feedforward of Akt/CREB in Alzheimer’s disease. Mol Ther Nucleic Acids 27:256–275. 10.1016/j.omtn.2021.11.019 35024240 PMC8714918

[124] Zhang H, Liu W, Ge H, Li K (2021) Aberrant expression of miR-148a-3p in Alzheimer’s disease and its protective role against amyloid-β induced neurotoxicity. Neurosci Lett 756:135953. 10.1016/j.neulet.2021.135953 33979697

[125] Satoh J-I, Kino Y, Niida S (2015) MicroRNA-seq data analysis pipeline to identify blood biomarkers for Alzheimer’s disease from public data. Biomark Insights 10:21–31. 10.4137/BMI.S25132 25922570 PMC4401249

[126] Chen J, Bai X, Wu Q, Chen L, Wang H, Zhang J (2023) Exercise protects against cognitive injury and inflammation in Alzheimer’s disease through elevating miR-148a-3p. Neuroscience 513:126–133. 10.1016/j.neuroscience.2023.01.008 36681141

[127] Garcia G, Pinto S, Ferreira S, Lopes D, Serrador MJ, Fernandes A, Vaz AR, de Mendonça A, et al. (2022) Emerging role of miR-21-5p in neuron-glia dysregulation and exosome transfer using multiple models of Alzheimer’s disease. Cells 11:3377. 10.3390/cells11213377 36359774 PMC9655962

[128] Pan S, Zhou Y, Wang X, Tong J, Li Y, Huang J, Chen S, Cui Y, et al. (2025) miRNA-associated gene networks reveal potential candidate markers for Alzheimer’s disease. Front Mol Biosci 12:1699404. 10.3389/fmolb.2025.1699404 41868603 PMC13002409

[129] Gámez-Valero A, Campdelacreu J, Vilas D, Ispierto L, Reñé R, Álvarez R, Armengol MP, Borràs FE, et al. (2019) Exploratory study on microRNA profiles from plasma-derived extracellular vesicles in Alzheimer’s disease and dementia with Lewy bodies. Transl Neurodegener 8:31. 10.1186/s40035-019-0169-5 31592314 PMC6775659

[130] Giuliani A, Gaetani S, Sorgentoni G, Agarbati S, Laggetta M, Matacchione G, Gobbi M, Rossi T, et al. (2021) Circulating inflamma-miRs as potential biomarkers of cognitive impairment in patients affected by Alzheimer’s disease. Front Aging Neurosci 13:647015. 10.3389/fnagi.2021.647015 33776746 PMC7990771

[131] Hashemi KS, Aliabadi MK, Mehrara A, Talebi E, Hemmati AA, Rezaeiye RD, Ghanbary MJ, Motealleh M, et al. (2023) A meta-analysis of microarray datasets to identify biological regulatory networks in Alzheimer’s disease. Front Genet 14:1225196. 10.3389/fgene.2023.1225196 37705610 PMC10497115

[132] Van der Auwera S, Ameling S, Wittfeld K, d’Harcourt Rowold E, Nauck M, Völzke H, Suhre K, Najafi-Shoushtari H, et al. (2019) Association of childhood traumatization and neuropsychiatric outcomes with altered plasma micro RNA-levels. Neuropsychopharmacology 44:2030–2037. 10.1038/s41386-019-0460-2 31284290 PMC6898678

[133] Agostini S, Mancuso R, Citterio LA, Caputo D, Oreni L, Nuzzi R, Pasanisi MB, Rovaris M, et al. (2024) Serum miR-34a-5p, miR-103a-3p, and miR-376a-3p as possible biomarkers of conversion from relapsing-remitting to secondary progressive multiple sclerosis. Neurobiol Dis 200:106648. 10.1016/j.nbd.2024.106648 39181188

[134] Zhou J, Zhao Y, Li Z, Zhu M, Wang Z, Li Y, Xu T, Feng D, et al. (2020) miR-103a-3p regulates mitophagy in Parkinson’s disease through Parkin/Ambra1 signaling. Pharmacol Res 160:105197. 10.1016/j.phrs.2020.105197 32942015

[135] Zhang Y, Wang J, Liu X, Li J, Fan S (2020) MicroRNA miR-103a-3p targets NPAS3 to regulate progression of Alzheimer’s disease. Trop J Pharm Res 19:1015–1021. 10.4314/tjpr.v19i5.16

[136] Xu H, Wang X-P, Mei S-L, Song Z, Yang J (2025) Exercise combined with cognitive rehabilitation training affects miR-103a-3p/BDNF expression to improve Alzheimer’s disease. Turk J Biochem. 10.1515/tjb-2025-0350

[137] Fertan E, Gendron WH, Wong AA, Hanson GM, Brown RE, Weaver ICG (2023) Noncanonical regulation of imprinted gene Igf2 by amyloid-beta 1–42 in Alzheimer’s disease. Sci Rep 13:2043. 10.1038/s41598-023-29248-x 36739453 PMC9899226

[138] De Felice B, Coppola C, Bonavita S, Signoriello E, Montanino C, Farinella F (2023) Exploring circulating long non-coding RNAs in mild cognitive impairment patients’ blood. Biomedicines 11:2963. 10.3390/biomedicines11112963 38001964 PMC10669861

[139] Allen CL, Bayraktutan U (2009) Oxidative stress and its role in the pathogenesis of ischaemic stroke. Int J Stroke 4:461–470. 10.1111/j.1747-4949.2009.00387.x 19930058

[140] Zhong L, Liu P, Fan J, Luo Y (2021) Long non-coding RNA H19: physiological functions and involvements in central nervous system disorders. Neurochem Int 148:105072. 10.1016/j.neuint.2021.105072 34058282

[141] Nandwani A, Rathore S, Datta M (2024) LncRNA H19 inhibition impairs endoplasmic reticulum-mitochondria contact in hepatic cells and augments gluconeogenesis by increasing VDAC1 levels. Redox Biol 69:102989. 10.1016/j.redox.2023.102989 38100882 PMC10761920

[142] Wang S-H, Zhu X-L, Wang F, Chen S-X, Chen Z-T, Qiu Q, Liu W-H, Wu M-X, et al. (2021) LncRNA H19 governs mitophagy and restores mitochondrial respiration in the heart through Pink1/Parkin signaling during obesity. Cell Death Dis 12:557. 10.1038/s41419-021-03821-6 34050133 PMC8163878

[143] Yılmaz ŞG, Erdal ME, Özge AA, Sungur MA (2016) Can peripheral microRNA expression data serve as epigenomic (upstream) biomarkers of Alzheimer’s disease? OMICS J Integr Biol 20:456–461. 10.1089/omi.2016.0099 27501295

[144] Quan X, Liang H, Chen Y, Qin Q, Wei Y, Liang Z (2020) Related network and differential expression analyses identify nuclear genes and pathways in the hippocampus of Alzheimer disease. Med Sci Monit. 10.12659/MSM.919311 31989994 PMC7001513

[145] Qayyum N, Haseeb M, Kim MS, Choi S (2021) Role of thioredoxin-interacting protein in diseases and its therapeutic outlook. Int J Mol Sci 22:2754. 10.3390/ijms22052754 33803178 PMC7963165

[146] Pan Q, Guo K, Xue M, Tu Q (2021) Estradiol exerts a neuroprotective effect on SH-SY5Y cells through the miR-106b-5p/TXNIP axis. J Biochem Mol Toxicol 35:e22861. 10.1002/jbt.22861 34318539

[147] Kim Y-J, Kim SH, Park Y, Park J, Lee JH, Kim BC, Song WK (2020) MiR-16-5p is upregulated by amyloid β deposition in Alzheimer’s disease models and induces neuronal cell apoptosis through direct targeting and suppression of BCL-2. Exp Gerontol 136:110954. 10.1016/j.exger.2020.110954 32320719

[148] Zhang N, Li W-W, Lv C-M, Gao Y-W, Liu X-L, Zhao L (2020) MiR-16-5p and miR-19b-3p prevent amyloid β-induced injury by targeting BACE1 in SH-SY5Y cells. NeuroReport 31:205. 10.1097/WNR.0000000000001379 31876684

[149] Huang D, Wen Q, Su Y, Li X miR-17-5p inhibits BNIP3-mediated mitochondrial autophagy to attenuate pathological cardiac fibrosis. Balk Med J 42:516–525. 10.4274/balkanmedj.galenos.2025.2025-6-25 PMC12576490 40801449

[150] Estfanous S, Daily KP, Eltobgy M, Deems NP, Anne MNK, Krause K, Badr A, Hamilton K, et al. (2021) Elevated expression of MiR-17 in microglia of Alzheimer’s disease patients abrogates autophagy-mediated amyloid-β degradation. Front Immunol 12:705581. 10.3389/fimmu.2021.705581 34426734 PMC8379081

[151] Chen M-L, Hong C-G, Yue T, Li H-M, Duan R, Hu W-B, Cao J, Wang Z-X, et al. (2021) Inhibition of miR-331-3p and miR-9-5p ameliorates Alzheimer’s disease by enhancing autophagy. Theranostics 11:2395–2409. 10.7150/thno.47408 33500732 PMC7797673

[152] Riancho J, Vázquez-Higuera JL, Pozueta A, Lage C, Kazimierczak M, Bravo M, Calero M, Gonalezález A, et al. (2017) MicroRNA profile in patients with Alzheimer’s disease: analysis of miR-9-5p and miR-598 in raw and exosome enriched cerebrospinal fluid samples. J Alzheimers Dis 57:483–491. 10.3233/JAD-161179 28269782

[153] Li W, Zheng Y (2023) MicroRNAs in extracellular vesicles of Alzheimer’s disease. Cells. 10.3390/cells12101378 37408212 PMC10216432

[154] Boyer-Guittaut M, Poillet L, Liang Q, Bôle-Richard E, Ouyang X, Benavides GA, Chakrama F-Z, Fraichard A, et al. (2014) The role of GABARAPL1/GEC1 in autophagic flux and mitochondrial quality control in MDA-MB-436 breast cancer cells. Autophagy 10:986–1003. 10.4161/auto.28390 24879149 PMC4091181

[155] Birgisdottir ÅB, Mouilleron S, Bhujabal Z, Wirth M, Sjøttem E, Evjen G, Zhang W, Lee R, et al. (2019) Members of the autophagy class III phosphatidylinositol 3-kinase complex I interact with GABARAP and GABARAPL1 via LIR motifs. Autophagy 15:1333–1355. 10.1080/15548627.2019.1581009 30767700 PMC6613885

[156] Mei T, Li Y, Orduña Dolado A, Li Z, Andersson R, Berliocchi L, Rasmussen LJ (2023) Pooled analysis of frontal lobe transcriptomic data identifies key mitophagy gene changes in Alzheimer’s disease brain. Front Aging Neurosci 15:1101216. 10.3389/fnagi.2023.1101216 37358952 PMC10288858

[157] Visconte C, Fenoglio C, Serpente M, Muti P, Sacconi A, Rigoni M, Arighi A, Borracci V, et al. (2023) Altered extracellular vesicle miRNA profile in prodromal Alzheimer’s disease. Int J Mol Sci 24:14749. 10.3390/ijms241914749 37834197 PMC10572781

[158] Wang L, Zhen H, Sun Y, Rong S, Li B, Song Z, Liu Z, Li Z, et al. (2022) Plasma exo-miRNAs correlated with AD-related factors of Chinese individuals involved in Aβ accumulation and cognition decline. Mol Neurobiol 59:6790–6804. 10.1007/s12035-022-03012-0 36040555 PMC9425792

[159] Kumar P, Dezso Z, MacKenzie C, Oestreicher J, Agoulnik S, Byrne M, Bernier F, Yanagimachi M, et al. (2013) Circulating miRNA biomarkers for Alzheimer’s disease. PLoS One 8:e69807. 10.1371/journal.pone.0069807 23922807 PMC3726785

[160] Poursaei E, Abolghasemi M, Bornehdeli S, Shanehbandi D, Asadi M, Sadeghzadeh M, Rahmanpour D, Sadeh RN (2022) Evaluation of hsa-let-7d-5p, hsa-let-7g-5p and hsa-miR-15b-5p plasma levels in patients with Alzheimer’s disease. Psychiatr Genet 32:25–29. 10.1097/YPG.0000000000000303 34955516

[161] Huang T, Wang X, Liu Y (2025) Silencing SOX2-OT alleviates A β-induced damage to neurons and microglial cells by targeting miR-143-3p in Alzheimer’s disease. Toxicol Res 14:tfaf159. 10.1093/toxres/tfaf159 PMC12640538 41287841

[162] Li D, Chen Y, Zhang T, Lv Z, Zhang L, Li X, Zhang A (2023) Profiling microRNA from peripheral blood mononuclear cells in early-onset familial Alzheimer’s disease. NeuroReport 34:178–183. 10.1097/WNR.0000000000001878 36719832

[163] Yang Q, Zhao Q, Yin Y (2019) MiR-133b is a potential diagnostic biomarker for Alzheimer’s disease and has a neuroprotective role. Exp Ther Med 18:2711–2718. 10.3892/etm.2019.7855 31572518 PMC6755445

[164] Darabi S, Gorgich EA, Rustamzadeh A, Mohebi N, Mozhdehipanah H (2025) The miR-133b and miR-206 serum levels as a candidate biomarker in Alzheimer’s patients with dyslipidemia. IBRO Neurosci Rep 18:672–678. 10.1016/j.ibneur.2025.04.014 40959537 PMC12434688

[165] Dobricic V, Schilling M, Schulz J, Zhu L-S, Zhou C-W, Fuß J, Franzenburg S, Zhu L-Q, et al. (2022) Differential microRNA expression analyses across two brain regions in Alzheimer’s disease. Transl Psychiatry 12:352. 10.1038/s41398-022-02108-4 36038535 PMC9424308

[166] Sierksma A, Lu A, Salta E, Vanden Eynden E, Callaerts-Vegh Z, D’Hooge R, Blum D, Buée L, et al. (2018) Deregulation of neuronal miRNAs induced by amyloid-β or TAU pathology. Mol Neurodegener 13:54. 10.1186/s13024-018-0285-1 30314521 PMC6186090

[167] McKeever PM, Schneider R, Taghdiri F, Weichert A, Multani N, Brown RA, Boxer AL, Karydas A, et al. (2018) MicroRNA expression levels are altered in the cerebrospinal fluid of patients with young-onset Alzheimer’s disease. Mol Neurobiol 55:8826–8841. 10.1007/s12035-018-1032-x 29603092 PMC6208843

[168] Tan A, Prasad R, Lee C, Jho E (2022) Past, present, and future perspectives of transcription factor EB (TFEB): mechanisms of regulation and association with disease. Cell Death Differ 29:1433–1449. 10.1038/s41418-022-01028-6 35739255 PMC9345944

[169] Calabrese C, Nolte H, Pitman MR, Ganesan R, Lampe P, Laboy R, Ripa R, Fischer J, et al. (2024) Mitochondrial translocation of TFEB regulates complex I and inflammation. EMBO Rep 25:704–724. 10.1038/s44319-024-00058-0 38263327 PMC10897448

[170] Kang Q, Xiang Y, Li D, Liang J, Zhang X, Zhou F, Qiao M, Nie Y, et al. (2017) MiR-124-3p attenuates hyperphosphorylation of tau protein-induced apoptosis via caveolin-1-PI3K/Akt/GSK3β pathway in N2a/APP695swe cells. Oncotarget 8:24314–24326. 10.18632/oncotarget.15149 28186985 PMC5421849

[171] Awuson-David B, Williams AC, Wright B, Hill LJ, Di Pietro V (2023) Common microRNA regulated pathways in Alzheimer’s and Parkinson’s disease. Front Neurosci. 10.3389/fnins.2023.1228927 37719162 PMC10502311

[172] Wu Y, Xu J, Xu J, Cheng J, Jiao D, Zhou C, Dai Y, Chen Q (2017) Lower serum levels of miR-29c-3p and miR-19b-3p as biomarkers for Alzheimer’s disease. Tohoku J Exp Med 242:129–136. 10.1620/tjem.242.129 28626163

[173] Sha S, Shen X, Cao Y, Qu L (2021) Mesenchymal stem cells-derived extracellular vesicles ameliorate Alzheimer’s disease in rat models via the microRNA-29c-3p/BACE1 axis and the Wnt/β-catenin pathway. Aging 13:15285–15306. 10.18632/aging.203088 34086603 PMC8221351

[174] Mou Y, Wang J, Wu J, He D, Zhang C, Duan C, Li B (2019) Ferroptosis, a new form of cell death: opportunities and challenges in cancer. J Hematol Oncol 12:34. 10.1186/s13045-019-0720-y 30925886 PMC6441206

[175] Yan N, Zhang J (2020) Iron metabolism, ferroptosis, and the links with Alzheimer’s disease. Front Neurosci 13:1443. 10.3389/fnins.2019.01443 32063824 PMC7000453

[176] Jakaria M, Belaidi AA, Bush AI, Ayton S (2021) Ferroptosis as a mechanism of neurodegeneration in Alzheimer’s disease. J Neurochem 159:804–825. 10.1111/jnc.15519 34553778

[177] Zhang Y, Zhang D, Xiao H (2026) Regulated neuronal death in Alzheimer’s disease: crosstalk and convergence of apoptosis, pyroptosis, senescence, and ferroptosis. J Alzheimer’s Dis 13872877261430002. 10.1177/13872877261430002 41808488

[178] Onukwufor JO, Dirksen RT, Wojtovich AP (2022) Iron dysregulation in mitochondrial dysfunction and Alzheimer’s disease. Antioxidants (Basel, Switzerland). 10.3390/antiox11040692 35453377 PMC9027385

[179] Xi J, Zhang B, Luo F, Liu J, Yang T (2012) Quercetin protects neuroblastoma SH-SY5Y cells against oxidative stress by inhibiting expression of Krüppel-like factor 4. Neurosci Lett 527:115–120. 10.1016/j.neulet.2012.08.082 22985515

[180] Javadov S (2022) Mitochondria and ferroptosis Curr Opin Physiol 25:100483. 10.1016/j.cophys.2022.100483 35342847 PMC8944045

[181] Gao M, Yi J, Zhu J, Minikes AM, Monian P, Thompson CB, Jiang X (2019) Role of mitochondria in ferroptosis. Mol Cell 73:354-363.e3. 10.1016/j.molcel.2018.10.042 30581146 PMC6338496

[182] Wang H, Liu C, Zhao Y, Gao G (2020) Mitochondria regulation in ferroptosis. Eur J Cell Biol 99:151058. 10.1016/j.ejcb.2019.151058 31810634

[183] Kushwaha S, Lakhanpal V, Elangovan A, Iyer M, Kinoshita M, Narayanasamy A, Reddy DH, Siama Z, et al. (2026) Unravelling the novel insights between miR-7 and Parkinson’s disease (PD). Neurol Sci 47:310. 10.1007/s10072-026-08834-7 41762282

[184] Sheng B, Wang X, Su B, Lee H, Casadesus G, Perry G, Zhu X (2012) Impaired mitochondrial biogenesis contributes to mitochondrial dysfunction in Alzheimer’s disease. J Neurochem 120:419–429. 10.1111/j.1471-4159.2011.07581.x 22077634 PMC3253532

[185] Palabiyik AA, Palabiyik E (2025) Pharmacological approaches to enhance mitochondrial biogenesis: focus on PGC-1Α, AMPK, and SIRT1 in cellular health. Mol Biol Rep 52:270. 10.1007/s11033-025-10368-8 40019682

[186] Han S, Lin F, Ruan Y, Zhao S, Yuan R, Ning J, Jiang K, Xie J, et al. (2021) MiR-132-3p promotes the cisplatin-induced apoptosis and inflammatory response of renal tubular epithelial cells by targeting SIRT1 via the NF-κB pathway. Int Immunopharmacol 99:108022. 10.1016/j.intimp.2021.108022 34339961

[187] Li C, Han S, Zhu J, Cheng F (2023) MiR-132-3p activation aggravates renal ischemia-reperfusion injury by targeting Sirt1/PGC1alpha axis. Cell Signal 110:110801. 10.1016/j.cellsig.2023.110801 37433399

[188] Zhang J-Y, Yang Z, Fang K, Shi Z-L, Ren D-H, Sun J (2020) Long noncoding RNA ILF3-AS1 regulates myocardial infarction via the miR-212-3p/SIRT1 axis and PI3K/Akt signaling pathway. Eur Rev Med Pharmacol Sci 24:2647–2658. 10.26355/eurrev_202003_20534 32196615

[189] Wang Z, Sun L, Jia K, Wang H, Wang X (2019) MiR-9-5p modulates the progression of Parkinson’s disease by targeting SIRT1. Neurosci Lett 701:226–233. 10.1016/j.neulet.2019.02.038 30826419

[190] Ryu IS, Kim DH, Cho H-J, Ryu J-H (2023) The role of microRNA-485 in neurodegenerative diseases. Rev Neurosci 34:49–62. 10.1515/revneuro-2022-0039 35793556

[191] Ryu IS, Kim DH, Ro J-Y, Park B-G, Kim SH, Im J-Y, Lee J-Y, Yoon SJ, et al. (2023) The microRNA-485-3p concentration in salivary exosome-enriched extracellular vesicles is related to amyloid β deposition in the brain of patients with Alzheimer’s disease. Clin Biochem 118:110603. 10.1016/j.clinbiochem.2023.110603 37355215

[192] Kornblum C, Nicholls TJ, Haack TB, Schöler S, Peeva V, Danhauser K, Hallmann K, Zsurka G, et al. (2013) Loss-of-function mutations in MGME1 impair mtDNA replication and cause multisystemic mitochondrial disease. Nat Genet 45:214–219. 10.1038/ng.2501 23313956 PMC3678843

[193] Matic S, Jiang M, Nicholls TJ, Uhler JP, Dirksen-Schwanenland C, Polosa PL, Simard M-L, Li X, et al. (2018) Mice lacking the mitochondrial exonuclease MGME1 accumulate mtDNA deletions without developing progeria. Nat Commun 9:1202. 10.1038/s41467-018-03552-x 29572490 PMC5865154

[194] Bazzani V, Equisoain Redin M, McHale J, Perrone L, Vascotto C (2022) Mitochondrial DNA repair in neurodegenerative diseases and ageing. Int J Mol Sci 23:11391. 10.3390/ijms231911391 36232693 PMC9569545

[195] Yang R, Rincon M (2016) Mitochondrial Stat3, the need for design thinking. Int J Biol Sci 12:532–544. 10.7150/ijbs.15153 27019635 PMC4807418

[196] Wegrzyn J, Potla R, Chwae Y-J, Sepuri NBV, Zhang Q, Koeck T, Derecka M, Szczepanek K, et al. (2009) Function of mitochondrial Stat3 in cellular respiration. Science 323:793–797. 10.1126/science.1164551 19131594 PMC2758306

[197] Garama DJ, White CL, Balic JJ, Gough DJ (2016) Mitochondrial STAT3: powering up a potent factor. Cytokine 87:20–25. 10.1016/j.cyto.2016.05.019 27269970

[198] Macias E, Rao D, Carbajal S, Kiguchi K, DiGiovanni J (2014) Stat3 binds to mtDNA and regulates mitochondrial gene expression in keratinocytes. J Invest Dermatol 134:1971–1980. 10.1038/jid.2014.68 24496235 PMC4057971

[199] Alawdi SH, El-Denshary ES, Safar MM, Eidi H, David M-O, Abdel-Wahhab MA (2017) Neuroprotective effect of nanodiamond in Alzheimer’s disease rat model: a pivotal role for modulating NF-κB and STAT3 signaling. Mol Neurobiol 54:1906–1918. 10.1007/s12035-016-9762-0 26897372

[200] Tigan A-S, Bellutti F, Kollmann K, Tebb G, Sexl V (2016) CDK6—a review of the past and a glimpse into the future: from cell-cycle control to transcriptional regulation. Oncogene 35:3083–3091. 10.1038/onc.2015.407 26500059

[201] Scheiblecker L, Klampfl T, Doma E, Nebenfuehr S, Torres-Quesada O, Strich S, Heller G, Werdenich D, et al. (2025) CDK6 kinase inhibition unmasks metabolic dependencies in BCR::ABL1+ leukemia. Cell Death Dis 16:107. 10.1038/s41419-025-07434-1 39966356 PMC11836434

[202] Liu F, Qiu F, Chen H (2021) MiR-124-3p ameliorates isoflurane-induced learning and memory impairment via targeting STAT3 and inhibiting neuroinflammation. NeuroImmunoModulation 28:248–254. 10.1159/000515661 34392240

[203] Usefi F, Rustamzadeh A, Ghobadi Z, Sadigh N, Mohebi N, Ariaei A, Moradi F (2024) Rosuvastatin attenuates total-tau serum levels and increases expression of miR-124-3p in dyslipidemic Alzheimer’s patients: a historic cohort study. Metab Brain Dis 39:1201–1211. 10.1007/s11011-024-01371-2 38896205

[204] Wang X, Liu D, Huang H-Z, Wang Z-H, Hou T-Y, Yang X, Pang P, Wei N, et al. (2018) A novel microRNA-124/PTPN1 signal pathway mediates synaptic and memory deficits in Alzheimer’s disease. Biol Psychiatry 83:395–405. 10.1016/j.biopsych.2017.07.023 28965984

[205] Évora A, Garcia G, Rubi A, De Vitis E, Matos AT, Vaz AR, Gervaso F, Gigli G, et al. (2025) Exosomes enriched with miR-124-3p show therapeutic potential in a new microfluidic triculture model that recapitulates neuron–glia crosstalk in Alzheimer’s disease. Front Pharmacol. 10.3389/fphar.2025.1474012 40144670 PMC11936931

[206] Ke J, Ding J, Xu Y, Yu C, Hong Y, Li S, Meng T, Ping Y, et al. (2026) Engineering microglial exosome-mediated microRNA-124-3p delivery for Alzheimer’s disease combinational therapy. Biomater Sci 14:186–197. 10.1039/D5BM01080B 41208730

[207] Angelopoulou E, Paudel YN, Piperi C (2019) miR-124 and Parkinson’s disease: a biomarker with therapeutic potential. Pharmacol Res 150:104515. 10.1016/j.phrs.2019.104515 31707035

[208] Vuokila N, Aronica E, Korotkov A, van Vliet EA, Nuzhat S, Puhakka N, Pitkänen A (2020) Chronic regulation of miR-124-3p in the perilesional cortex after experimental and human TBI. Int J Mol Sci. 10.3390/ijms21072418 32244461 PMC7177327

[209] Ge X, Guo M, Hu T, Li W, Huang S, Yin Z, Li Y, Chen F, et al. (2020) Increased microglial exosomal miR-124-3p alleviates neurodegeneration and improves cognitive outcome after rmTBI. Mol Ther 28:503–522. 10.1016/j.ymthe.2019.11.017 31843449 PMC7001001

[210] Taşdelen E, Özel Kizil ET, Tezcan S, Yalap E, Bi̇ngöl AP, Yürür Kutlay N Determination of miR-373 and miR-204 levels in neuronal exosomes in Alzheimer’s disease. Turk J Med Sci 52:1458–1467. 10.55730/1300-0144.5484 PMC10395704 36422510

[211] Almawashee HS, Khalaj-Kondori M, Feizi MAH, Safaralizadeh R (2026) Correlation of miR-214, miR-204, miR-25, miR-15a expression with IL-33 and malondialdehyde in blood samples from patients with Alzheimer’s disease. 10.2174/0122115366411857251025004952 41589493

[212] Schneider R, McKeever P, Kim T, Graff C, van Swieten JC, Karydas A, Boxer A, Rosen H et al (2018) Downregulation of exosomal miR-204-5p and miR-632 as a biomarker for FTD: a GENFI study. J Neurol Neurosurg Psychiatry 89:851–858. 10.1136/jnnp-2017-317492 29434051 PMC6045452

[213] Tan YJ, Wong BYX, Vaidyanathan R, Sreejith S, Chia SY, Kandiah N, Ng ASL, Zeng L (2021) Altered cerebrospinal fluid exosomal microRNA levels in young-onset Alzheimer’s disease and frontotemporal dementia. J Alzheimers Dis Rep 5:805–813. 10.3233/ADR-210311 34870106 PMC8609483

[214] Han S-W, Park YH, Pyun J-M, Bice PJ, Kim S, Saykin AJ, Nho K (2025) miR-423-5p and miR-92a-3p in Alzheimer’s disease: relationship with pathology and cognition. Front Aging Neurosci 17:1637368. 10.3389/fnagi.2025.1637368 40766177 PMC12321855

[215] Zhu X, Lu X (2019) MiR-423-5p inhibition alleviates cardiomyocyte apoptosis and mitochondrial dysfunction caused by hypoxia/reoxygenation through activation of the wnt/β-catenin signaling pathway via targeting MYBL2. J Cell Physiol 234:22034–22043. 10.1002/jcp.28766 31074036

[216] Bocchetti M, Cossu AM, Porru M, Ferraro MG, Irace C, Tufano R, Vitale G, Misso G, et al. (2025) MiR-423-5p is a metabolic and growth tuner in hepatocellular carcinoma via MALAT-1 and mitochondrial interaction. J Exp Clin Cancer Res 44:270. 10.1186/s13046-025-03524-2 41029313 PMC12487375

[217] Tan L, Yu J-T, Tan M-S, Liu Q-Y, Wang H-F, Zhang W, Jiang T, Tan L (2014) Genome-wide serum microRNA expression profiling identifies serum biomarkers for Alzheimer’s disease. J Alzheimers Dis 40:1017–1027. 10.3233/JAD-132144 24577456

[218] Chen F, Zhao Y, Chen H (2018) MicroRNA-98 reduces amyloid β-protein production and improves oxidative stress and mitochondrial dysfunction through the Notch signaling pathway via HEY2 in Alzheimer’s disease mice. Int J Mol Med. 10.3892/ijmm.2018.3957 30365070 PMC6257854

[219] Ye F, Wu A (2021) The protective mechanism of SIRT1 in the regulation of mitochondrial biogenesis and mitochondrial autophagy in Alzheimer’s disease. J Alzheimers Dis 82:149–157. 10.3233/JAD-210132 33998544

[220] Wan H-L, Hong X-Y, Zhao Z-H, Li T, Zhang B-G, Liu Q, Wang Q, Zhao S, et al. (2021) STAT3 ameliorates cognitive deficits via regulation of NMDAR expression in an Alzheimer’s disease animal model. Theranostics 11:5511–5524. 10.7150/thno.56541 33859760 PMC8039956

[221] Chiba T, Yamada M, Aiso S (2009) Targeting the JAK2/STAT3 axis in Alzheimer’s disease. Expert Opin Ther Targets 13:1155–1167. 10.1517/14728220903213426 19663649

[222] Aaluri GR, Choudhary Y, Kumar S (2024) Mitochondria-associated microRNAs and Parkinson’s disease. Neurosci Insights 19:26331055241254850. 10.1177/26331055241254846 38800624 PMC11127579

[223] Wang H, Ye Y, Zhu Z, Mo L, Lin C, Wang Q, Wang H, Gong X, et al. (2016) MiR -124 regulates apoptosis and autophagy process in MPTP model of Parkinson’s disease by targeting to Bim. Brain Pathol 26:167–176. 10.1111/bpa.12267 25976060 PMC8029438

[224] Yardeni T, Fine R, Joshi Y, Gradus-Pery T, Kozer N, Reichenstein I, Yanowski E, Nevo S, et al. (2018) High content image analysis reveals function of miR-124 upstream of vimentin in regulating motor neuron mitochondria. Sci Rep 8:59. 10.1038/s41598-017-17878-x 29311649 PMC5758812

[225] Grel H, Woznica D, Ratajczak K, Kalwarczyk E, Anchimowicz J, Switlik W, Olejnik P, Zielonka P, et al. (2023) Mitochondrial dynamics in neurodegenerative diseases: unraveling the role of fusion and fission processes. Int J Mol Sci. 10.3390/ijms241713033 37685840 PMC10487704

[226] Wang X, Su B, Lee H, Li X, Perry G, Smith MA, Zhu X (2009) Impaired balance of mitochondrial fission and fusion in Alzheimer’s disease. J Neurosci 29:9090–9103. 10.1523/JNEUROSCI.1357-09.2009 19605646 PMC2735241

[227] Misrani A, Tabassum S, Yang L (2021) Mitochondrial dysfunction and oxidative stress in Alzheimer’s disease. Front Aging Neurosci. 10.3389/fnagi.2021.617588 33679375 PMC7930231

[228] Yu R, Lendahl U, Nistér M, Zhao J (2020) Regulation of mammalian mitochondrial dynamics: opportunities and challenges. Front Endocrinol 11:374. 10.3389/fendo.2020.00374 PMC7300174 32595603

[229] Adebayo M, Singh S, Singh AP, Dasgupta S (2021) Mitochondrial fusion and fission: the fine-tune balance for cellular homeostasis. FASEB J 35:e21620. 10.1096/fj.202100067R 34048084 PMC8415099

[230] Shen Q, Yamano K, Head BP, Kawajiri S, Cheung JTM, Wang C, Cho J-H, Hattori N, et al. (2014) Mutations in Fis1 disrupt orderly disposal of defective mitochondria. Mol Biol Cell 25:145–159. 10.1091/mbc.e13-09-0525 24196833 PMC3873885

[231] Losón OC, Song Z, Chen H, Chan DC (2013) Fis1, Mff, MiD49, and MiD51 mediate Drp1 recruitment in mitochondrial fission. Mol Biol Cell 24:659–667. 10.1091/mbc.e12-10-0721 23283981 PMC3583668

[232] Yu R, Jin S, Lendahl U, Nistér M, Zhao J (2019) Human Fis1 regulates mitochondrial dynamics through inhibition of the fusion machinery. EMBO J 38:e99748. 10.15252/embj.201899748 30842096 PMC6463211

[233] Eberhardt EL, Ludlam AV, Tan Z, Cianfrocco MA (2020) Miro: a molecular switch at the center of mitochondrial regulation. Protein Sci 29:1269–1284. 10.1002/pro.3839 32056317 PMC7255519

[234] Ding L, Lei Y, Han Y, Li Y, Ji X, Liu L (2016) Vimar is a novel regulator of mitochondrial fission through Miro. PLoS Genet 12:e1006359. 10.1371/journal.pgen.1006359 27716788 PMC5065127

[235] Gao Z, Zhang R, Jiang L, Zhou H, Wang Q, Ma Y, Zhang D, Qin Y, et al. (2022) Administration of miR-195 inhibitor enhances memory function through improving synaptic degradation and mitochondrial dysfunction of the hippocampal neurons in SAMP8 mice. J Alzheimers Dis 85:1495–1509. 10.3233/JAD-215301 34924391

[236] Kumar S, Morton H, Sawant N, Orlov E, Bunquin LE, Pradeepkiran JA, Alvir R, Reddy PH (2021) MicroRNA-455-3p improves synaptic, cognitive functions and extends lifespan: relevance to Alzheimer’s disease. Redox Biol 48:102182. 10.1016/j.redox.2021.102182 34781166 PMC8604688

[237] Pradeepkiran JA, Bandaru M, Islam MA, Kshirsagar S, Alvir RV, Mukherjee U, Reddy PH (2026) Overexpression of miR-455-3p enhances mitophagy, synaptic and mitochondrial proteins in Alzheimer’s disease. Mitochondrion 89:102151. 10.1016/j.mito.2026.102151 41932415

[238] Islam MA, Sultana OF, Bandari M, Kshirsagar S, Manna PR, Reddy PH (2024) MicroRNA-455-3P as a peripheral biomarker and therapeutic target for mild cognitive impairment and Alzheimer’s disease. Ageing Res Rev 100:102459. 10.1016/j.arr.2024.102459 39153602 PMC11383742

[239] Kumar S, Reddy PH (2018) MicroRNA-455-3p as a potential biomarker for Alzheimer’s disease: an update. Front Aging Neurosci 10:41. 10.3389/fnagi.2018.00041 29527164 PMC5829054

[240] Chiara F, Rasola A (2013) GSK-3 and mitochondria in cancer cells. Front Oncol. 10.3389/fonc.2013.00016 23386998 PMC3564062

[241] Yang K, Chen Z, Gao J, Shi W, Li L, Jiang S, Hu H, Liu Z, et al. (2017) The key roles of GSK-3β in regulating mitochondrial activity. Cell Physiol Biochem 44:1445–1459. 10.1159/000485580 29190615

[242] Moldovan RA, Hidalgo MR, Castañé H, Jiménez-Franco A, Joven J, Burks DJ, Galán A, García-García F (2025) Landscape of sex differences in obesity and type 2 diabetes in subcutaneous adipose tissue: a systematic review and meta-analysis of transcriptomics studies. Metabolism 168:156241. 10.1016/j.metabol.2025.156241 40157598

[243] Liu J, Xiao Q, Xiao J, Niu C, Li Y, Zhang X, Zhou Z, Shu G, et al. (2022) Wnt/β-catenin signalling: function, biological mechanisms, and therapeutic opportunities. Signal Transduct Target Ther 7:3. 10.1038/s41392-021-00762-6 34980884 PMC8724284

[244] Wasim M, Guo J, Wang Z, Parveen R, Chen R, Wang Y, Ma G (2025) miR-137: a therapeutic candidate or a key molecular regulator in Alzheimer’s disease? J Alzheimers Dis Rep 9:25424823251352170. 10.1177/25424823251352166 40575117 PMC12198550

[245] Geekiyanage H, Jicha GA, Nelson PT, Chan C (2012) Blood serum miRNA: non-invasive biomarkers for Alzheimer’s disease. Exp Neurol 235:491–496. 10.1016/j.expneurol.2011.11.026 22155483 PMC3361462

[246] Channakkar AS, Singh T, Pattnaik B, Gupta K, Seth P, Adlakha YK (2020) MiRNA-137-mediated modulation of mitochondrial dynamics regulates human neural stem cell fate. Stem Cells 38:683–697. 10.1002/stem.3155 32012382 PMC7217206

[247] Li W, Zhang X, Zhuang H, Chen H, Chen Y, Tian W, Wu W, Li Y, et al. (2014) MicroRNA-137 is a novel hypoxia-responsive microRNA that inhibits mitophagy via regulation of two mitophagy receptors FUNDC1 and NIX. J Biol Chem 289:10691–10701. 10.1074/jbc.M113.537050 24573672 PMC4036186

[248] Russell AE, Jun S, Sarkar S, Geldenhuys WJ, Lewis SE, Rellick SL, Simpkins JW (2019) Extracellular vesicles secreted in response to cytokine exposure increase mitochondrial oxygen consumption in recipient cells. Front Cell Neurosci 13:51. 10.3389/fncel.2019.00051 30837842 PMC6383587

[249] Kou X, Li J, Liu X, Chang J, Zhao Q, Jia S, Fan J, Chen N (2017) Swimming attenuates d-galactose-induced brain aging via suppressing miR-34a-mediated autophagy impairment and abnormal mitochondrial dynamics. J Appl Physiol 122:1462–1469. 10.1152/japplphysiol.00018.2017 28302704

[250] Jauhari A, Singh T, Mishra S, Shankar J, Yadav S (2020) Coordinated action of miR-146a and Parkin gene regulate rotenone-induced neurodegeneration. Toxicol Sci 176:433–445. 10.1093/toxsci/kfaa066 32392329

[251] Giuliani A, Cirilli I, Prattichizzo F, Mensà E, Fulgenzi G, Sabbatinelli J, Graciotti L, Olivieri F, et al. (2018) The mitomiR/Bcl-2 axis affects mitochondrial function and autophagic vacuole formation in senescent endothelial cells. Aging (Albany NY) 10:2855–2873. 10.18632/aging.101591 30348904 PMC6224225

[252] Krützfeldt J, Rajewsky N, Braich R, Rajeev KG, Tuschl T, Manoharan M, Stoffel M (2005) Silencing of microRNAs in vivo with ‘antagomirs.’ Nature 438:685–689. 10.1038/nature04303 16258535

[253] Wang W-X, Prajapati P, Vekaria H, Spry M, Cloud A, Sullivan P, Springer J (2021) Temporal changes in inflammatory mitochondria-enriched microRNAs following traumatic brain injury and effects of miR-146a nanoparticle delivery. Neural Regen Res 16:514. 10.4103/1673-5374.293149 32985480 PMC7996041

[254] Barbosa M, Gomes C, Sequeira C, Gonçalves-Ribeiro J, Pina CC, Carvalho LA, Moreira R, Vaz SH, et al. (2021) Recovery of depleted miR-146a in ALS cortical astrocytes reverts cell aberrancies and prevents paracrine pathogenicity on microglia and motor neurons. Front Cell Dev Biol 9:634355. 10.3389/fcell.2021.634355 33968923 PMC8103001

[255] Palmer CS, Anderson AJ, Stojanovski D (2021) Mitochondrial protein import dysfunction: mitochondrial disease, neurodegenerative disease and cancer. FEBS Lett 595:1107–1131. 10.1002/1873-3468.14022 33314127

[256] Devi L, Prabhu BM, Galati DF, Avadhani NG, Anandatheerthavarada HK (2006) Accumulation of amyloid precursor protein in the mitochondrial import channels of human Alzheimer’s disease brain is associated with mitochondrial dysfunction. J Neurosci 26:9057–9068. 10.1523/JNEUROSCI.1469-06.2006 16943564 PMC6675337

[257] Wiedemann N, Pfanner N (2017) Mitochondrial machineries for protein import and assembly. Annu Rev Biochem 86:685–714. 10.1146/annurev-biochem-060815-014352 28301740

[258] Heinemeyer T, Stemmet M, Bardien S, Neethling A (2019) Underappreciated roles of the translocase of the outer and inner mitochondrial membrane protein complexes in human disease. DNA Cell Biol 38:23–40. 10.1089/dna.2018.4292 30481057

[259] Tao W, Yu L, Shu S, Liu Y, Zhuang Z, Xu S, Bao X, Gu Y, et al. (2021) MiR-204-3p/Nox4 mediates memory deficits in a mouse model of Alzheimer’s disease. Mol Ther 29:396–408. 10.1016/j.ymthe.2020.09.006 32950103 PMC7791017

[260] Lian X, Zhang X, Chen W, Xue F, Wang G (2024) Dexmedetomidine mitigates neuroinflammation in an Alzheimer’s disease mouse model via the miR-204-3p/FBXL7 signaling axis. Brain Res 1822:148612. 10.1016/j.brainres.2023.148612 37778649

[261] Tan Y, Yu Y, Niu H, Wang C, Mo P, Li D, Zhang Q, Feng D, et al. (2024) Profile of miRNA expression in the hippocampus of epileptic mice and the prediction of potential therapeutic targets. Mol Biol Rep 51:929. 10.1007/s11033-024-09861-3 39172288

[262] Baker MJ, Crameri JJ, Thorburn DR, Frazier AE, Stojanovski D (2022) Mitochondrial biology and dysfunction in secondary mitochondrial disease. Open Biol 12:220274. 10.1098/rsob.220274 36475414 PMC9727669

[263] Michaelis JB, Brunstein ME, Bozkurt S, Alves L, Wegner M, Kaulich M, Pohl C, Münch C (2022) Protein import motor complex reacts to mitochondrial misfolding by reducing protein import and activating mitophagy. Nat Commun 13:5164. 10.1038/s41467-022-32564-x 36056001 PMC9440083

[264] Zhang W, Yang Y, Xiang Z, Cheng J, Yu Z, Wang W, Hu L, Ma F, et al. (2022) MRTF-A-mediated protection against amyloid-β-induced neuronal injury correlates with restoring autophagy via miR-1273g-3p/mTOR axis in Alzheimer models. Aging (Albany NY) 14:4305–4325. 10.18632/aging.203883 35604830 PMC9186769

[265] Shoshan-Barmatz V, Nahon-Crystal E, Shteinfer-Kuzmine A, Gupta R (2018) VDAC1, mitochondrial dysfunction, and Alzheimer’s disease. Pharmacol Res 131:87–101. 10.1016/j.phrs.2018.03.010 29551631

[266] Manczak M, Reddy PH (2012) Abnormal interaction of VDAC1 with amyloid beta and phosphorylated tau causes mitochondrial dysfunction in Alzheimer’s disease. Hum Mol Genet 21:5131–5146. 10.1093/hmg/dds360 22926141 PMC3490521

[267] Camara AKS, Zhou Y, Wen P-C, Tajkhorshid E, Kwok W-M (2017) Mitochondrial VDAC1: a key gatekeeper as potential therapeutic target. Front Physiol 8:460. 10.3389/fphys.2017.00460 28713289 PMC5491678

[268] Manczak M, Sheiko T, Craigen WJ, Reddy PH (2013) Reduced VDAC1 protects against Alzheimer’s disease, mitochondria, and synaptic deficiencies. J Alzheimers Dis 37:679–690. 10.3233/JAD-130761 23948905 PMC3925364

[269] Verma A, Shteinfer-Kuzmine A, Kamenetsky N, Pittala S, Paul A, Nahon Crystal E, Ouro A, Chalifa-Caspi V, et al. (2022) Targeting the overexpressed mitochondrial protein VDAC1 in a mouse model of Alzheimer’s disease protects against mitochondrial dysfunction and mitigates brain pathology. Transl Neurodegener 11:58. 10.1186/s40035-022-00329-7 36578022 PMC9795455

[270] Haddad DM, Vilain S, Vos M, Esposito G, Matta S, Kalscheuer VM, Craessaerts K, Leyssen M, et al. (2013) Mutations in the intellectual disability gene Ube2a cause neuronal dysfunction and impair Parkin-dependent mitophagy. Mol Cell 50:831–843. 10.1016/j.molcel.2013.04.012 23685073

[271] Fiesel FC, Moussaud-Lamodière EL, Ando M, Springer W (2014) Select E2 enzymes differentially regulate parkin activation and mitophagy. J Cell Sci. 10.1242/jcs.147520 24928900 PMC4132391

[272] Geisler S, Vollmer S, Golombek S, Kahle PJ (2014) UBE2N, UBE2L3 and UBE2D2/3 ubiquitin-conjugating enzymes are essential for parkin-dependent mitophagy. J Cell Sci. 10.1242/jcs.146035 24906799

[273] Zhao Y, Alexandrov P, Jaber V, Lukiw W (2016) Deficiency in the ubiquitin conjugating enzyme UBE2A in Alzheimer’s disease (AD) is linked to deficits in a natural circular miRNA-7 sponge (circRNA; ciRS-7). Genes 7:116. 10.3390/genes7120116 27929395 PMC5192492

[274] Bakhti SZ, Dadashi S, Dah Pahlevan A, Kafshresan F (2021) Functional roles and biological mechanisms of circular RNAs and their encoded peptides in glioma: a narrative review. Int Clin Neurosci J 8:157–167. 10.34172/icnj.2021.33

[275] Lagunas-Rangel FA (2024) Role of circular RNAs in DNA repair. RNA Biol 21:1226–1238. 10.1080/15476286.2024.2429945 PMC11572198 39550713

[276] Bao X, Zhang C, Ren Z, Wang Y, Zeng L (2025) Multi-omics integration analysis revealed that miR-375-3p is a two-sided factor regulating the development and tumorigenesis of Alzheimer’s disease. Int J Mol Sci. 10.3390/ijms26083666 40332288 PMC12027480

[277] Wan Q, Kuang E, Dong W, Zhou S, Xu H, Qi Y, Liu Y (2007) Reticulon 3 mediates Bcl-2 accumulation in mitochondria in response to endoplasmic reticulum stress. Apoptosis 12:319–328. 10.1007/s10495-006-0574-y 17191123

[278] Chen R, Jin R, Wu L, Ye X, Yang Y, Luo K, Wang W, Wu D, et al. (2011) Reticulon 3 attenuates the clearance of cytosolic prion aggregates via inhibiting autophagy. Autophagy 7:205–216. 10.4161/auto.7.2.14197 21119307

[279] Juranek J, Mukherjee K, Kordas B, Załęcki M, Korytko A, Zglejc-Waszak K, Szuszkiewicz J, Banach M (2022) Role of RAGE in the pathogenesis of neurological disorders. Neurosci Bull 38:1248–1262. 10.1007/s12264-022-00878-x 35729453 PMC9554177

[280] Baazaoui N, Y. Alfaifi M, Ben Saad R, Garzoli S (2025) Potential role of long noncoding RNA maternally expressed gene 3 (MEG3) in the process of neurodegeneration. Neuroscience 565:487–498. 10.1016/j.neuroscience.2024.12.023 39675694

[281] Farhadieh M-E, Ghaedi K (2023) Analyzing alternative splicing in Alzheimer’s disease postmortem brain: a cell-level perspective. Front Mol Neurosci 16:1237874. 10.3389/fnmol.2023.1237874 37799732 PMC10548223

[282] Lai Y, Lin H, Chen M, Lin X, Wu L, Zhao Y, Lin F, Lin C (2023) Integration of bulk RNA sequencing and single-cell analysis reveals a global landscape of DNA damage response in the immune environment of Alzheimer’s disease. Front Immunol 14:1115202. 10.3389/fimmu.2023.1115202 36895559 PMC9989175

[283] Wang Z, Xia P, Hu J, Huang Y, Zhang F, Li L, Wang E, Guo Q, et al. (2021) LncRNA MEG3 alleviates diabetic cognitive impairments by reducing mitochondrial-derived apoptosis through promotion of FUNDC1-related mitophagy via Rac1-ROS axis. ACS Chem Neurosci 12:2280–2307. 10.1021/acschemneuro.0c00682 33843209

[284] Zhang J, Wang C, Yang G, Zhou Y, Hou D, Xia Y (2025) Olfactory mucosal mesenchymal stem cell-derived exosome Lnc A2M-AS1 ameliorates oxidative stress by regulating TP53INP1-mediated mitochondrial autophagy through interacting with IGF2BP1 in Parkinson’s diseases. Cell Biol Toxicol 41:60. 10.1007/s10565-025-10009-7 40111649 PMC11926059

[285] Bijur GN, Jope RS (2003) Rapid accumulation of Akt in mitochondria following phosphatidylinositol 3-kinase activation. J Neurochem 87:1427–1435. 10.1046/j.1471-4159.2003.02113.x 14713298 PMC2040497

[286] He W, Duan L, Zhang L (2023) LOXL1-AS1 aggravates myocardial ischemia/reperfusion injury through the miR-761/PTEN axis. Korean Circ J 53:387. 10.4070/kcj.2022.0301 37161797 PMC10244188

[287] Ren N, Jiang T, Wang C, Xie S, Xing Y, Piao D, Zhang T, Zhu Y (2020) LncRNA ADAMTS9-AS2 inhibits gastric cancer (GC) development and sensitizes chemoresistant GC cells to cisplatin by regulating miR-223-3p/NLRP3 axis. Aging (Albany NY) 12:11025–11041. 10.18632/aging.103314 32516127 PMC7346038

[288] Miao R, Jiang C, Chang WY, Zhang H, An J, Ho F, Chen P, Zhang H, et al. (2023) Gasdermin D permeabilization of mitochondrial inner and outer membranes accelerates and enhances pyroptosis. Immunity 56:2523-2541.e8. 10.1016/j.immuni.2023.10.004 37924812 PMC10872579

[289] Zheng K, Huang H, Liu D, Brazhe N, Chen J, Zhu L (2025) Targeting the miR-96-5p/cathepsin B pathway to alleviate neuron-derived neuroinflammation in Alzheimer’s disease. MedComm 6:e70368. 10.1002/mco2.70368 40919134 PMC12413563

[290] Zhu M, Jia L, Jia J (2021) Inhibition of miR-96-5p may reduce Aβ42/Aβ40 ratio via regulating ATP-binding cassette transporter A1. J Alzheimers Dis 83:367–377. 10.3233/JAD-210411 34334400

[291] Brown GC (2007) Mechanisms of inflammatory neurodegeneration: iNOS and NADPH oxidase. Biochem Soc Trans 35:1119–1121. 10.1042/BST0351119 17956292

[292] Guo Y, Wen J, He A, Qu C, Peng Y, Luo S, Wang X (2023) I<changed>n</changed>OS contributes to heart failure with preserved ejection fraction through mitochondrial dysfunction and Akt S-nitrosylation. J Adv Res 43:175–186. 10.1016/j.jare.2022.03.003 36585107 PMC9811328

[293] Xie Y, Xie D, Chen C (2024) Hsa_circ_0049472 contributed to amyloid-beta peptide-induced neurotoxicity, apoptosis and inflammation via regulating PI3K-AKT signaling pathway by interacting with miR-22–3p/ZNF217 axis. Brain Res Bull 215:111004. 10.1016/j.brainresbull.2024.111004 38852653

[294] Sun Z, Sun L, Tu L (2020) GABAB receptor-mediated PI3K/Akt signaling pathway alleviates oxidative stress and neuronal cell injury in a rat model of Alzheimer’s disease. J Alzheimers Dis 76:1513–1526. 10.3233/JAD-191032 32651311

[295] Lu C-H, Chen D-X, Dong K, Wu Y-J, Na N, Wen H, Hu Y, Liang Y-Y, et al. (2023) Inhibition of miR-143-3p alleviates myocardial ischemia reperfusion injury via limiting mitochondria-mediated apoptosis. Biol Chem 404:619–631. 10.1515/hsz-2022-0334 36780323

[296] Wang J, Chen C, Zhang Y (2020) An investigation of microRNA-103 and microRNA-107 as potential blood-based biomarkers for disease risk and progression of Alzheimer’s disease. J Clin Lab Anal 34:e23006. 10.1002/jcla.23006 31420923 PMC6977154

[297] Wingo TS, Yang J, Fan W, Min Canon S, Gerasimov ES, Lori A, Logsdon B, Yao B, et al. (2020) Brain microRNAs associated with late-life depressive symptoms are also associated with cognitive trajectory and dementia. Npj Genomic Med 5:6. 10.1038/s41525-019-0113-8 PMC7004995 32047652

[298] Lin T, Pu X, Zhou S, Huang Z, Chen Q, Zhang Y, Mao Q, Liang Y, et al. (2023) Identification of exosomal miR-484 role in reprogramming mitochondrial metabolism in pancreatic cancer through Wnt/MAPK axis control. Pharmacol Res 197:106980. 10.1016/j.phrs.2023.106980 37944835

[299] Wang K, Long B, Jiao J-Q, Wang J-X, Liu J-P, Li Q, Li P-F (2012) MiR-484 regulates mitochondrial network through targeting Fis1. Nat Commun 3:781. 10.1038/ncomms1770 22510686

[300] Zhang Z, Zhang T, Feng R, Huang H, Xia T, Sun C (2019) CircARF3 alleviates mitophagy-mediated inflammation by targeting miR-103/TRAF3 in mouse adipose tissue. Mol Ther Nucleic Acids 14:192–203. 10.1016/j.omtn.2018.11.014 30623853 PMC6325073

[301] Zheng Q, Bi R, Xu M, Zhang D-F, Tan L-W, Lu Y-P, Yao Y-G (2021) Exploring the genetic association of the ABAT gene with Alzheimer’s disease. Mol Neurobiol 58:1894–1903. 10.1007/s12035-020-02271-z 33404980

[302] Ciminelli BM, Menduti G, Benussi L, Ghidoni R, Binetti G, Squitti R, Rongioletti M, Nica S, et al. (2020) Polymorphic genetic markers of the GABA catabolism pathway in Alzheimer’s disease. J Alzheimers Dis 77:301–311. 10.3233/JAD-200429 32804142

[303] Besse A, Wu P, Bruni F, Donti T, Graham BH, Craigen WJ, McFarland R, Moretti P, et al. (2015) The GABA transaminase, ABAT, is essential for mitochondrial nucleoside metabolism. Cell Metab 21:417–427. 10.1016/j.cmet.2015.02.008 25738457 PMC4757431

[304] Shen J, Wang C, Li D, Wang T, McAlinden A, O’Keefe R (2018) Methylation of 4-aminobutyrate aminotransferase (ABAT) by dnmy3b regulates chondrocytes metabolism and the development of osteoarthritis (OA). Osteoarthritis Cartil 26:S64–S65. 10.1016/j.joca.2018.02.137

[305] Hao W, Luo Q, Szabo IM, Gasparoni G, Tierling S, Quan W, Chang H-F, Wu G, et al. (2025) Intestinal epithelial Dicer1 regulates gut microbiome and Alzheimer’s pathology in App-knock-in mice. Alzheimers Res Ther 17:202. 10.1186/s13195-025-01849-w 40926280 PMC12418706

[306] Su X, Jin Y, Shen Y, Kim I, Weintraub NL, Tang Y (2019) RNAase III-type enzyme dicer regulates mitochondrial fatty acid oxidative metabolism in cardiac mesenchymal stem cells. Int J Mol Sci 20:5554. 10.3390/ijms20225554 31703292 PMC6888515

[307] Tong X, Yu N, Han R, Wang T (2020) Function of Dicer with regard to energy homeostasis regulation, structural modification, and cellular distribution. Int J Endocrinol 2020:6420816. 10.1155/2020/6420816 32774363 PMC7397435

[308] Kandeil MA, Mohammed ET, Ibrahim MA, Radi RA, Gamal A, Abdel-Razik A-RH, Khalil F, Sabry D (2025) Formulation and evaluation of astaxanthin-loaded invasomes as therapeutic approaches for Alzheimer’s disease induced in rats: role of SIRT-1/BDNF/miRNA-134/GSK-3β signaling. Mol Neurobiol 62:16189–16208. 10.1007/s12035-025-05241-5 40748435 PMC12559087

[309] Abozaid OAR, Sallam MW, Ahmed ESA (2022) Mesenchymal stem cells modulate SIRT1/MiR-134/GSK3β signaling pathway in a rat model of Alzheimer’s disease. J Prev Alzheimers Dis 9:458–468. 10.14283/jpad.2022.26 35841247

[310] Abozaid OAR, Sallam MW, El-Sonbaty S, Aziza S, Emad B, Ahmed ESA (2022) Resveratrol-selenium nanoparticles alleviate neuroinflammation and neurotoxicity in a rat model of Alzheimer’s disease by regulating Sirt1/miRNA-134/GSK3β expression. Biol Trace Elem Res 200:5104–5114. 10.1007/s12011-021-03073-7 35059981

[311] Zhou Y, Wang Y, Wang Y, Chen L, Wang N, Su Y, Diwu Y, Zhang Q (2023) LncRNA NKILA exacerbates Alzheimer’s disease progression by regulating the FOXA1-mediated transcription of TNFAIP1. Neurochem Res 48:2895–2910. 10.1007/s11064-023-03944-6 37217807

[312] García-Escudero V, Martín-Maestro P, Perry G, Avila J (2013) Deconstructing mitochondrial dysfunction in Alzheimer disease. Oxid Med Cell Longev. 10.1155/2013/162152 23840916 PMC3693159

[313] Ilieva MS (2024) Non-coding RNAs in neurological and neuropsychiatric disorders: unraveling the hidden players in disease pathogenesis. Cells 13:1063. 10.3390/cells13121063 38920691 PMC11201512

[314] Patrick E, Rajagopal S, Wong H-KA, McCabe C, Xu J, Tang A, Imboywa SH, Schneider JA, et al. (2017) Dissecting the role of non-coding RNAs in the accumulation of amyloid and tau neuropathologies in Alzheimer’s disease. Mol Neurodegener 12:51. 10.1186/s13024-017-0191-y 28668092 PMC5494142

[315] Novotný JS, Čarná M, Dammer EB, Mao Z, Stokin GB (2026) Rethinking Alzheimer’s: novel miRNAs illuminate a disease beyond the brain. Mol Psychiatry 1–10. 10.1038/s41380-026-03487-6 PMC13269133 41688789

[316] Kenny A, McArdle H, Calero M, Rabano A, Madden SF, Adamson K, Forster R, Spain E, et al. (2019) Elevated plasma microRNA-206 levels predict cognitive decline and progression to dementia from mild cognitive impairment. Biomolecules 9:734. 10.3390/biom9110734 31766231 PMC6920950

[317] Guévremont D, Tsui H, Knight R, Fowler CJ, Masters CL, Martins RN, Abraham WC, Tate WP, et al. (2022) Plasma microRNA vary in association with the progression of Alzheimer’s disease. Alzheimers Dement Diagn Assess Dis Monit 14:e12251. 10.1002/dad2.12251 PMC8817674 35141392

[318] Winkle M, El-Daly SM, Fabbri M, Calin GA (2021) Noncoding RNA therapeutics — challenges and potential solutions. Nat Rev Drug Discov 20:629–651. 10.1038/s41573-021-00219-z 34145432 PMC8212082

