# Supplementary material for: Non-coding RNAs and Mitochondrial Dysfunction in Alzheimer’s Disease: A Systematic Review
Source: Mol Neurobiol. 2026 Sep 15;63(1):894. doi: 10.1007/s12035-026-06198-9 (PMC13574927; doi:10.1007/s12035-026-06198-9)
Supplement: Supplementary file 1 [file 12035_2026_6198_MOESM1_ESM.docx]

**Molecular Neurobiology**

**Non-coding RNAs and Mitochondrial Dysfunction in Alzheimer's Disease: A Systematic Review**

**Ștefania-Maria Mocrei-Rebrean1, Vlad Răzniceanu1, Sebastian Romeo Pintilie1, Ioana Berindan-Neagoe2,3,4**

1 Faculty of Medicine, Iuliu Hațieganu University of Medicine and Pharmacy, Cluj-Napoca, Romania

2 Department of Genomics, MEDFUTURE Institute for Biomedical Research, Iuliu Hațieganu University of Medicine and Pharmacy, Cluj-Napoca, Romania

3 Doctoral School, Iuliu Hațieganu University of Medicine and Pharmacy, Cluj-Napoca, Romania

4 Academy of Medical Sciences, Bucharest, Romania

**Corresponding author: Vlad Răzniceanu, razniceanu.vlad@elearn.umfcluj.ro**

**Table 2** MicroRNA dysregulation and mitochondrial dysfunction in AD: overview of translational and fundamental studies

| **MiRNA - Translational Studies** | | | | |
| --- | --- | --- | --- | --- |
| Reference | Tissue sample | Subjects or experimental model | RNA quantification methods | Results |
| [51] | Brain | AD patients and controls  Meta-analysis:   - 36 transcriptomic datasets (7 for RNA-seq and 29 for microarray) - 45 differential miR expression studies | N/A | AD vs controls:   - ↓ miR-132-3p (logOR = −5.08; 95% CI −6.41 to −3.74; FDR = 1.19 × 10⁻12) - ↓ miR-212-3p (logOR = −6.37; 95% CI −7.53 to −5.21; FDR = 2.38 × 10⁻25) - miR-9-5p frequently reported as differentially expressed, but not among the statistically significant meta-analytic DEmiRNAs |
| [52] | Brain | Discovery datasets:   - Religious Orders Study and the Rush Memory and Aging Project - miR cohorts: control (n=180), MCI (n=111), AD (n=147), other (n=12) - mRNA cohorts: control (n=200), MCI (n=165), AD (n=251), other (n=12)   Validation dataset:   - Alzheimer's Disease Neuroimaging Initiative - mRNA cohorts: control (n=246), MCI (n=383), AD (n=114) | N/A | AD vs controls:   - ↓ miR-129-5p (FDR < 0.01) - ↓ miR-132 (FDR < 0.01)   ceRNA regulatory axes:   - miR-132-3p – NEAT1 – circ_0132052 - miR-129-5p – MALAT1 – circ_0066962 |
| [53]^e^ | Brain | Training set: GSE5281  n= 87 controls  n= 74 AD samples | N/A | AD vs controls:   - ↓ HIBCH and MGME1 (>2-fold; p < 0.001) - miR-922 predicted and experimentally validated as a direct regulator of MGME1 |
|  | Blood | Validation set: GSE63061  n= 139 AD samples  n=249 controls |  |  |
| [54] | Brain | GSE118553  GSE48350  GSE33000  n= 377 AD patients  n= 223 controls | N/A | AD vs controls:   - miR-103 and miR-484 identified as predicted regulators of mitochondrial hub genes (UQCRFS1, NDUFS2, NDUFS3, NDUFS1, SDHB, MDH2, PDHA1, NDUFS8, NDUFB10 and NDUFV1) - miR-103 showed a reported interaction with NDUFV1; the role of miR-103/miR-484 in AD remains insufficiently characterized |
| [55] | Brain (frontal cortex) | n=10 AD  n=12 controls  n=12 nondemented with Alzheimer's neuropathology (NDAN) | RT-qPCR | AD vs controls and NDAN:   - ↑ miR-485-5p vs controls (p = 0.007) - ↑ miR-485-5p vs NDAN (p = 0.003) - no significant difference between controls and NDAN (p = 0.0909) - ↓ PGC1α in AD; elevated miR-485-5p was proposed to contribute to PGC1α inhibition |
| [56] | Brain (hippocampus) | NeuroPro database: 11 proteomic studies of human AD hippocampal tissue | N/A | AD hippocampus:   - miR-106b-5p, miR-17-5p and miR-16-5p identified as regulators associated with altered proteins - associated target proteins enriched in ribosomal function, autophagy and immune-system pathways |
| [57] | Brain (temporal neocortex) | n=3 AD patients (79.3 ± 9.2 years)  n=3 controls (78.4 ± 5.7 years) | array-based miRNA-mRNA analysis and miRNA-mRNA-based linking assay | AD vs age-, sex- and PMI-matched controls:   - ↑ miR-9, miR-34a, miR-146a and miR-155 (1.7- to 3.3-fold) - miR-9, miR-34a, miR-146a and miR-155 have recognition sites in the SIRT1 mRNA 3′UTR - their upregulation was proposed to contribute to ↓ SIRT1 expression in AD |
| [58]^e^ | Plasma | n=36 controls  n=24 aMCI  n=36 AD | Microarray for  miRNAs | AD vs controls (\|FC\| > 1.5; p < 0.05):   - ↑ miR-1273g-3p - ↑ miR-8063 - ↓ miR-6798-3p - ↓ miR-106b-5p - ↓ miR-363-3p   aMCI vs controls (\|FC\| > 1.5; p < 0.05):   - ↑ miR-1273g-3p   ↑ miR-375 |
|  | Plasma | n=31 control  n=12 PSAD (presymptomatic AD)  n=20 PDAD (prodromal AD)  n=20 AD | RT-qPCR | AD vs controls:   - ↑ miR-1273g-3p (p < 0.05)   AD vs PSAD:   - ↑ miR-1273g-3p (p < 0.01)   PDAD vs controls:   - no significant difference in miR-1273g-3p |
|  | CSF | n=13 controls  n=10 PSAD  n=13 PDAD  n=14 AD | RT-qPCR | PSAD vs controls:   - ↑ miR-1273g-3p (p < 0.01)   PDAD vs controls:   - ↑ miR-1273g-3p (p < 0.01)   AD vs controls:   - ↑ miR-1273g-3p (p < 0.001)   CSF miR-1273g-3p negatively correlated with CSF Aβ42 (r = -0.501; p < 0.001) |
|  | Brain (hippocampus) | Prefrontal cortex RNA-seq dataset:  n=383 AD  n=165 controls  Post-mortem hippocampus:  n=8 AD  n=5 controls | N/A | AD vs controls:   - ↓ GLRX5, MTCH1 and TIMM13 mRNA in prefrontal cortex - ↓ TIMM13 protein in hippocampal CA1, CA2 and CA3 |
| [59]^e^ | Brain (temporal neocortex) | n=13 AD patients  n=10 controls | RT-qPCR | AD vs age-matched controls:   - ↑ miR-146a (3.6-fold; p ≤ 0.05) - miR-146a negatively correlated with VDAC1 protein levels (ρ = -0.80; p ≤ 0.01) |
| [60]^e^ | Brain (hippocampus) | n=6 AD  n=6 controls | RT-qPCR | AD vs controls:   - ↓ miR-218-5p and miR-124-3p (p < 0.05) - ↑ RTN3 protein (p < 0.001) |
| [61]^e^ | Brain | GSE173955  n=8 AD (hippocampus)  n=10 controls | N/A | AD vs controls:   - ↓ OPA1 (p = 0.031) - ↓ MFN2, not significant (p = 0.058)   AD vs controls:   - ↓ OPA1 (p = 0.029) - ↓ MFN2 (p = 0.00042) |
|  |  | GSE183260  n = 19 AD (whole brain)  n = 21 controls | N/A |  |
| [62] | Brain | GSE5281:  n=87 AD  n=74 controls  Validation dataset GSE132903:  n=97 AD  n=98 controls  miRDB/HMDD databases:  hub gene-targeting AD-associated miRNAs | N/A | GSE5281, AD vs controls:   - ↑ TFEB (logFC = 1.80; Padj = 1.75 × 10^-14) - ↓ TOMM20 (logFC = -1.01; Padj = 1.77 × 10^-6) - ↓ GABARAPL1 (logFC = -1.01; Padj = 1.57 × 10^-9)   Expression patterns of TFEB, TOMM20 and GABARAPL1 confirmed in GSE132903 (p < 0.001)  miRDB/HMDD:   - TFEB predicted targets: miR-29a-3p, miR-124-3p, miR-29b-3p, miR-29c-3p - TOMM20 predicted targets: let-7a-2-3p, miR-204-3p - GABARAPL1 predicted targets: miR-195-5p, miR-16-5p, miR-155-5p, miR-497-5p, miR-15b-5p, miR-143-3p, miR-133b |
| [64]^e^ | Brain (frontal cortex) | n=27 AD (65–95 years old)  n=15 controls (65–103 years old) | RT-qPCR | AD vs controls:   - ↑ miRNA PC-5P-12969 (p = 0.0008) |
| [83] | Whole blood | Statistical and machine learning analysis  N=7 AD (>65 years old)  N=4 controls (>65 years old) | Small RNA-Sequencing | AD compared to controls (peripheral blood):  ↓miR-6501-5p (log2FC = −1.34159, p = 0.003957)  ↓miR-143-3p (log2FC = −0.69404, p = 0.048802)  Predicted mitochondrial target genes:   - miR-6501-5p: predicted to target ATP5E - miR-143-3p: predicted to target NDUFB9, NDUFA9, NDUFS1 and COX4I1   Associated mitochondria-relevant terms as per Gene Ontology (GO) analysis:   - **miR-6501-5p:** respiratory electron transport chain; generation of precursor metabolites and energy - **miR-143-3p:** respiratory electron transport chain; generation of precursor metabolites and energy; mitochondrial inner membrane; mitochondrial electron transport; NADH dehydrogenase activity; mitochondrial respiratory chain complex I |
| [84]^e^ | Brain | Hippocampus,  medial frontal gyrus, and cerebellum samples  from age-matched n=27 subjects:  n=2 Braak stage 0 (non-demented)  n=5 Braak stage 1 (non-demented)  n=5 Braak stage 3 (non-demented)  n=5 each Braak stages 4, 5, and 6 (AD) | RT-qPCR | Braak stages 5–6 AD vs Braak stages 0–1 controls:  Hippocampus: ↑ miR-423 (p < 0.05)  Medial frontal gyrus: ↑ miR-423 (p < 0.05)  Cerebellum: ↓ miR-98 (p < 0.05) |
| **MiRNA – Fundamental Studies** | | | | |
| Reference | Tissue sample | Subjects or experimental model | RNA quantification methods | Results |
| [48] | Cells | SK-N-SH and CHP212 cells treated with Aβ | RT-qPCR | Aβ-treated vs control cells:  - ↓ miR-22-3p (p < 0.001)  Mechanistic validation:   - miR-22-3p mimic vs miR-NC: ↓ PDE4A - miR-22-3p directly targets the PDE4A 3′UTR - miR-22-3p overexpression protected against Aβ-induced neuronal injury |
| [50] | Brain (hippocampus) | APP/PS1 mice | RT-qPCR | APP/PS1 vs WT littermates:   - ↓ Dicer1 mRNA (4 months: p = 0.0335; 11 months: p = 0.0167) - ↓ Dicer1 protein (4 months: p = 0.0092; 11 months: p = 0.0002) - ↓ Nrf2 mRNA (4 months: p = 0.0304; 11 months: p = 0.0195) - ↓ Nrf2 protein (4 months: p = 0.0001; 11 months: p = 0.0038) - ↑ activated caspase-3 protein (4 months: p = 0.0098; 11 months: p = 0.0008) |
|  | Cells | Primary murine hippocampal neurons | RT-qPCR | Dicer1 siRNA vs NC siRNA:   - increased ROS (p < 0.0001) - decreased MMP (p = 0.0399) - ↑ activated caspase-3 protein (p = 0.020) |
|  | Cells | Neuro-2a cells | RT-qPCR | Aβ42 vs vehicle:   - ↓ Dicer1 protein (p = 0.0004) - ↓ Nrf2 protein   Nrf2 overexpression vs vehicle plasmid:   - ↑ Dicer1 mRNA (p = 0.0482) - ↑ Dicer1 protein (p = 0.024)   Mechanistic validation:   - Nrf2 directly regulates Dicer1 transcription through ARE sites in the Dicer1 promoter |
| [53] | Cells | 293T cells co-transfected with MGME1 3′UTR luciferase reporter constructs and miR-922 or miR-98-5p | N/A | MGME1-3′UTR-WT + miR-922 vs MGME1-3′UTR-WT + miR-NC:   - decreased luciferase activity by 42% (p = 0.003) - confirmed direct binding of miR-922 to MGME1 3′UTR   MGME1-3′UTR + miR-98-5p:   - no specific binding to MGME1 |
| [58] | Cells | H4-APPswe and SH-SY5Y cells | RT-qPCR | H4-APPswe and SH-SY5Y + miR-1273g-3p mimic vs mimic NC:   - ↑ miR-1273g-3p (p < 0.01) - decreased MMP and mitochondrial respiration - increased mitochondrial fragmentation and oxidative stress - ↓ GLRX5, MTCH1 and TIMM13   Rescue experiments:   - restored mitochondrial respiration and reversed BACE1/Aβ42-related changes |
| [59] | Cells | Primary cortical neurons from Sprague Dawley rats treated with Aβ | RT-qPCR | Aβ-treated vs untreated primary cortical neurons:   - ↑ intracellular miR-146a (p ≤ 0.05) - ↑ EV miR-146a (p ≤ 0.0001) |
|  | Cells | Primary mixed glial cells from Sprague Dawley rats | RT-qPCR | Aβ-treated vs untreated primary mixed glial cells:   - ↑ intracellular miR-146a (p ≤ 0.0001) - ↑ EV miR-146a (p ≤ 0.01)   miR-146a overexpression vs vector control in primary mixed glia:   - ↑ intracellular miR-146a (>2-fold) - ↑ EV miR-146a (53–116-fold) - decreased ATP-linked respiration, maximal respiration and spare respiratory capacity, glycolysis, glycolytic capacity and glycolytic reserve |
| [60] | Brain (dentate gyrus) | Male 3× Tg-AD mice subjected to social isolation  Adult male C57BL/6 mice (wild-type) subjected to social isolation | RT-qPCR | Socially isolated vs group-housed AD and WT mice:   - ↓ miR-218-5p and miR-124-3p   Socially isolated AD mice + miR-218-5p/miR-124-3p agomirs vs scramble:   - restored DG-CA3 synaptic plasticity and pattern separation   Mechanistic validation:   - miR-218-5p and miR-124-3p directly bind the Rtn3 3′UTR and posttranscriptionally suppress RTN3 protein - RTN3 upregulation/aggregation disrupted mitochondrial distribution in presynaptic boutons |
| [61] | Brain | 3xTg-AD mice | RT-qPCR | 3×Tg-AD vs WT mice:   - ↑ miR-323-5p and miR-370-3p (>2-fold, p < 0.001) - ↓ OPA1 and MFN2 protein |
|  | Cells | Adult rat hippocampal neural stem/progenitor (HCN) cells   - insulin withdrawal - miR-150-3p, miR-323-5p or miR-370-3p transfection | Microarray for miRNAs | Insulin withdrawal vs control:   - ↑ miR-150-3p, miR-323-5p and miR-370-3p   miRNA transfection vs control:   - ↓ OPA1 - miR-150-3p additionally ↓ MFN2 - decreased mitochondrial respiration and ATP production - increased mitochondrial fission   Mechanistic validation:   - miR-150-3p directly binds the MFN2 3′UTR (30% reduction in reporter activity, p < 0.001) - OPA1 is a potential target of all three miRNAs |
| [63] | Cells | HCN cells | RT-qPCR | miR-351-5p mimic vs miR-Con:   - ↓ Miro2 mRNA by 71% (p < 0.0001) - ↓ Miro2 protein by 41% (p < 0.01) - increased mitochondrial fission, mitophagy, autophagy-dependent cell death - decreased MMP and ATP production   Mechanistic validation:   - miR-351-5p directly targets the Miro2 3′UTR (37% reduction in reporter activity; p < 0.001) |
|  | Brain (hippocampus) | AD model: 5xFAD mice, 3xTg-AD mice  WT mice | RT-qPCR | 3xTg-AD and 5xFAD vs WT mice:   - ↑ miR-351-5p - ↓ Miro2 |
| [64] | Brain (cortex) | APP transgenic mice (Tg2576), 6 months old (n = 5)  Tau transgenic mice (P301L), 6 months old (n = 5)  Aβ knock-in mice (hA-KI), 12 months old (n = 5)  Wild-type C57BL/6 mice (n = 5) | RT-qPCR | vs respective WT controls:   - ↑ miRNA PC-5P-12969 in APP mice (p = 0.0117) - ↑ miRNA PC-5P-12969 in Tau mice (p = 0.0110) - no significant change in 6-month hA-KI mice (p = 0.9606) - ↑ miRNA PC-5P-12969 in 12-month hA-KI mice (p = 0.0362) |
|  | Cells | HT22 cells (untreated)  HT22 cells + miRNA PC-5P-12969 vector  HT22 cells + mAPP plasmid vector  HT22 cells + miRNA PC-5P-12969 + mAPP vectors (pre-condition)  HT22 cells + mAPP + miRNA PC-5P-12969 vectors (post-condition) | RT-qPCR | vs untreated HT22 cells:   - ↑ miRNA PC-5P-12969 with mAPP (p < 0.0001) - ↑ miRNA PC-5P-12969 with miRNA PC-5P-12969 vector (p < 0.0001) - ↑ miRNA PC-5P-12969 in post-condition (p = 0.0001) - no significant change in pre-condition (p = 0.9693)   Post-condition vs mAPP:   - ↓ GSK3α and APP - increased ATP production and maximal mitochondrial respiration - increased mitochondrial length - decreased mitochondrial fragmentation   Mechanistic validation:   - miRNA PC-5P-12969 directly binds the GSK3α 3′UTR (p < 0.0001) |
| [65] | Brain | *n* = 3 APP/PSEN1 double transgenic mice  *n* = 3 wild-type (WT) mice | NanoString miRNA expression assay;  RT-qPCR | AD cortex and hippocampus vs. controls:   - ↑ miR-7a (cortex and hippocampus) - ↑ miR-148a (hippocampus) - ↓ miR-34a (cortex) - ↓ miR-128a (hippocampus)   ↑ Klf4 protein (brain – immunofluorescence and Western Blot) |
|  | Cells | N9 microglial cells primed with lipopolysaccharide, followed by treatment with Aβ oligomers for 24 h (neuroinflammation model) | N/A | miR-7a-mediated Klf4 silencing resulted in:   - decreased iNOS   ↓ NLRP3 expression |
|  | Cells | SH-SY5Y cells | N/A | miR-7a mimic vs negative control: ↓ Klf4  miR-7a inhibitor treatment: ↑ Klf4 |
|  | Cells | SH-SY5Y cells treated with RAS-selective lethal 3 and vehicle (ferroptosis model) | N/A | RSL3-treated SH-SY5Y vs SH-SY5Y: ↑ Klf4 mRNA and protein  miR-7a mimic treatment followed by RSL3 resulted in:   - decreased ROS - normal long tubular mitochondrial morphology   MMP recovery |
| [65] | Brain | 12-month-old APP/PSEN1 double-transgenic mice (n = 3)  WT mice (n = 3) | NanoString miRNA expression assay;  RT-qPCR | APP/PSEN1 vs WT mice:   - cortex: ↑ miR-7a (2.05-fold); ↓ miR-34a - hippocampus: ↑ miR-7a and miR-148a; ↓ miR-128a - ↑ Klf4 protein (p = 0.015 by immunofluorescence; p < 0.05 by western blot) |
|  | Cells | N9 microglial cells primed with lipopolysaccharide, followed by treatment with Aβ oligomers for 24 h | RT-qPCR | LPS-primed N9 + AβO + miR-7a mimic vs LPS-primed N9 + AβO:   - decreased ROS - ↓ iNOS and NLRP3 |
|  | Cells | SH-SY5Y cells treated with RAS-selective lethal 3 and vehicle (ferroptosis model) | RT-qPCR | RSL3 + miR-7a mimic vs RSL3:   - ↓ Klf4 - decreased ROS - restored normal long tubular mitochondrial morphology - increased MMP (complete recovery at 250 nM miR-7a)   Mechanistic validation:   - - miR-7a directly binds the Klf4 3′UTR and suppresses Klf4 |
| [66] | Brain | n=42 APP/PS1 mice  n=6 wild type C57BL/6J mice | RT‐qPCR | AD vs WT:   - ↑ miR-9-5p (p < 0.001) - ↓ SIRT1 - decreased ATP, MMP and mitochondrial respiratory function   AD + Rg1 vs AD + Vehicle 1:   - ↓ miR-9-5p (p < 0.001) - ↑ SIRT1 - improved mitochondrial function   AD + Rg1 + miR-9-5p agomir vs AD + Rg1 + agomir NC:   - ↑ miR-9-5p (p < 0.05) - ↓ SIRT1 - increased mitochondrial dysfunction |
|  | Cells | Mouse hippocampal neuron HT-22 treated with Aβ_1–42_ (20 µM) | RT‐qPCR | Aβ1-42 vs blank:   - ↑ miR-9-5p (p < 0.001) - ↓ SIRT1 - ↓ PINK1, Parkin, LC3B II/I and Beclin-1   Aβ1-42 + Rg1 vs Aβ1-42 + Vehicle 1:   - ↓ miR-9-5p (p < 0.001) - ↑ SIRT1 - ↑ PINK1, Parkin, LC3B II/I and Beclin-1   Aβ1-42 + Rg1 + miR-9-5p mimic vs Aβ1-42 + Rg1 + mimic NC:   - ↑ miR-9-5p (p < 0.05) - ↓ SIRT1 - ↓ PINK1, Parkin, LC3B II/I and Beclin-1 - decreased ATP, MMP and mitochondrial respiratory function |
| [67] | Cells | Primary human neuronal cultures from human induced pluripotent stem cells (hiPSCs) exposed to 1 µM Aβ-42o | RT‐qPCR | hiPSC-NSC-EVs (containing miR-148a-3p, miR-21-5p and miR-103a-3p) + Aβ-42o vs Aβ-42o alone:   - decreased mitochondrial superoxide - ↓ BAD and BAX; ↑ BCL-2 - increased MMP and preserved TOM20+ mitochondria - ↑ Beclin-1 and MAP1LC3B - normalized mitochondrial complex gene expression |
| [68] | Brain | Male Sprague-Dawley rats, induced via aggregated injection Aβ1-42 into the hippocampus | RT-qPCR | AD vs normal rat hippocampus:   - ↑ miR-140 (p < 0.05) - ↓ PINK1   miR-140 antagomir vs NC-antagomir:   - ↑ PINK1 - increased autophagy |
|  | Cells | Primary hippocampal neurons from 1-day-old Sprague-Dawley rats treated with Aβ-derived diffusible ligands | RT-qPCR | AβDDLs-treated vs control neurons:   - ↑ miR-140 (p < 0.05) - ↓ PINK1 - increased ROS and mitochondrial dysfunction; decreased autophagy   miR-140 inhibitor vs NC-inhibitor:   - ↓ miR-140 (p < 0.05) - ↑ PINK1 - increased MMP and autophagy; decreased ROS |
| [69] | Cells | Mouse hippocampal neuron cell line HT22 treated with Aβ25-35 | RT-qPCR | Aβ25-35-treated vs untreated HT22 cells:   - ↓ miR-9-5p (p < 0.05) - ↑ GSK-3β   Aβ25-35 + miR-9-5p mimic vs Aβ25-35 + NC mimic:   - ↑ miR-9-5p (p < 0.01) - ↓ GSK-3β - decreased ROS/oxidative stress and apoptosis - ↓ cytochrome C, AIF, cleaved caspase-3 and BAX; ↑ BCL-2 |
| [70] | Brain (hippocampus) | SAMP8 mice aged 3-, 6- and 9- months (hippocampus) | Microarray for miRNAs;  RT-qPCR | SAMP8 vs age-matched SAMR1 hippocampus:   - ↑ miR-195 at 3 and 6 months (p < 0.05); no significant change at 9 months - ↓ miR-15b at 3, 6 and 9 months (p < 0.05) - ↓ MFN2 mRNA and protein |
|  | Cells | HT-22 hippocampal neuronal cells transfected with miR-195 mimics | N/A | miR-195 mimic vs scramble in HT-22 cells:   - ↑ miR-195 (p < 0.05) - ↓ MFN2 protein - decreased MMP |
| [71] | Cells | Rat hippocampal neurons treated with Aβ1-42  Normal hippocampal neurons (control) | RT-qPCR | Aβ1–42-treated vs control neurons:   - ↑ miR-204 (p < 0.05) - ↓ TRPML1 - increased mitochondrial dysfunction/ROS and impaired mitochondrial autophagy   Aβ1–42 + miR-204 inhibitor vs Aβ1–42 + inhibitor negative-control:   - ↑ TRPML1 - ↓ PINK1, Parkin, LC3II and Beclin1 - increased MMP; decreased ROS and mitochondrial autophagy   Aβ1–42 + CLN (STAT3 activation) vs Aβ1–42 + vehicle:   - ↓ miR-204 (p < 0.05) - ↑ TRPML1 - increased MMP and ATP; decreased ROS and mitochondrial autophagy |
|  | Brain | APP/PS1 transgenic AD mouse model | RT-qPCR | APP/PS1 vs normal hippocampus:   - ↑ miR-204 (p < 0.05) - ↓ TRPML1 - decreased mitochondrial autophagy and increased mitochondrial ROS |
| [72] | Cells | SH-SWE cells (transfected with human APP695) | RT-qPCR | SH-SWE vs SH-WT:   - ↑ miR-124, miR-125b and miR-146a (p < 0.05); ↑ miR-21 and miR-155 (p < 0.01) - ↑ exosomal miR-124 (p < 0.05) and miR-155 (p < 0.01)   miR-124 mimic vs mock in SH-SWE:   - ↑ miR-124 (70-fold; p < 0.01) - ↓ exosomal miR-125b (p < 0.05), miR-146a and miR-155 (p < 0.01); miR-21 decreased, NS - increased mitochondrial activity / MitoTracker intensity   miR-124 inhibitor vs mock in SH-SWE:   - ↓ miR-124 (≥80% decrease; p < 0.01) - ↑ miR-21 (p < 0.05) and miR-146a (p < 0.01); ↓ miR-125b (p < 0.05) - increased MFN2 protein; no significant change in DRP1 |
|  | Cells | iNEU-PSEN (iNeurons from iPSCs generated from a patient carrying the PSEN1ΔE9 mutation) and iNEU-wild type from a healthy female control | RT-qPCR | iNEU-PSEN vs iNEU-WT:   - ↑ miR-124, miR-125b and miR-21 (p < 0.05) - ↓ miR-146a and miR-155 (p < 0.05) - ↑ exosomal miR-124 and miR-155 (p < 0.01)   miR-124 mimic vs mock in iNEU-PSEN:   - ↑ miR-124 (~12-fold; p < 0.01) - ↓ miR-125b (p < 0.01) and miR-21 (p < 0.05) - ↓ exosomal miR-21 and miR-146a (p < 0.05) and miR-155 (p < 0.01); miR-125b decreased, NS   miR-124 inhibitor vs mock in iNEU-PSEN:   - ↓ miR-124 (≥80% decrease; p < 0.01) - ↓ miR-125b (p < 0.05) and miR-21 (p < 0.01); ↑ miR-146a (p < 0.01) |
| [73] | Cells | APP Swedish SH-SY5Y cells (SWE) | RT-qPCR | SWE vs wild-type SH-SY5Y (SH):   - ↑ miR-124 in cells and secretome (cellular, exosomal, and EXO-free; p < 0.05) |
|  | Cells | Human CHME3 microglial cells treated with IFNγ and co-cultured with SWE cells | RT-qPCR | IFNγ-microglia co-cultured with SWE vs IFNγ-microglia monoculture:   - ↑ miR-124 in microglia (nearly 90-fold; p < 0.001)   IFNγ-microglia co-cultured with miR-124-mimic SWE vs IFNγ-microglia co-cultured with mock SWE:   - ↑ miR-124 in SWE cells (70-fold; p < 0.01) and secretome (exosomal and EXO-free; p < 0.05) - ↑ miR-124 and ↓ miR-146a in co-cultured microglia (p < 0.01) - ↓ iNOS and TNF-α expression in microglia - increased mitochondrial activity / MitoTracker Red intensity |
| [74] | Cells | N2a mouse neuroblastoma cells transfected with mutant APP cDNA | N/A | mutant APP + miR-455-3p vs mutant APP   - increased mitochondrial biogenesis - decreased mitochondrial fragmentation - ↓ mitochondrial fission markers (DRP1 and FIS1) - ↑ mitochondrial fusion markers (OPA1, MFN1, and MFN2) |
| [81] | Cells | human neural stem cells (hNSCs) (H9-derived) treated with Aβ1–42 +/- gold nanoparticles (AuNPs) | Microarray for miRNAs;  RT-qPCR | Aβ + AuNPs vs Aβ   - ↑ miR-21-5p (FC = 4.78), miR-132, miR-107, miR-369, miR-181c, and miR-212 - ↓ miR-34a and miR-138 - ↑ DRP1, NRF1, and TFAM mRNA - increased MMP and decreased mitochondrial ROS - decreased apoptosis   Aβ + AuNPs + miR-21-5p inhibitor vs Aβ + AuNPs + NC   - ↓ miR-21-5p (p < 0.01) - ↓ DRP1, NRF1, and TFAM mRNA - decreased cell viability - decreased MMP, increased mitochondrial ROS, and increased apoptosis   miR-21-5p mimic vs mimic-NC   - ↑ miR-21-5p (p < 0.01) - ↓ SOCS6 protein - increased cell viability and decreased apoptosis |
| [82] | Cells | SH-SY5Y cells treated with Aβ25-35 | RT-qPCR | SH + Aβ25–35 + miR-375-3p vs SH + NC + Aβ25–35   - decreased Aβ25–35-induced apoptosis - decreased intracellular ROS - increased MMP / JC-1 polymer:monomer fluorescence ratio   AD + miR-375-3p vs AD control   - ↑ LC3B, Beclin-1, MFN2 and PINK1 mRNAs |
| [85] | Brain | n=10 3×Tg-AD mice receiving PBS injections in the tail veil  n=10 3×Tg-AD mice receiving CeO_2_-DRP/MA 1 mmol/L (CeO_2_ nanoparticles covalently linked with mitochondrial aptamers (MA) and DNA-RNA hybrid chains (DRP) to detect and silence mitochondrial miRNA-204) injections in the tail vein  n=10 C57BL/6 mice | N/A | 3×Tg-AD treated with CeO_2_-DRP/MA vs PBS-treated 3×Tg-AD mice:   - Decreased escape latency (p < 0.05) - Increased swimming speed (Morris water maze) - Decreased depression-related and anxiety-related behaviors   Hippocampal tissue of CeO_2_-DRP/MA treated vs PBS-treated 3×Tg-AD mice:   - ↓ PINK1, Beclin1, and LC3B mRNA/protein - ↑TRPML1 and p-STAT3 mRNA/protein |
|  | Cells | SY5Y+Aβ cells | RT-qPCR | SY5Y+Aβ + CeO2-DRP/MA (1 mmol/L) vs untreated SY5Y+Aβ cells:   - increased cell viability from ~50% at 0 mmol/L to 98% at 1 mmol/L after 24 h - increased MMP - ↓ mitochondrial miRNA-204 (p < 0.01) - ↓PINK1, Beclin-1, and LC3B mRNA - ↑TRPML1 and STAT3 mRNA |
|  | Cells | N2aSW cells | N/A | CeO2-DRP/MA-treated vs untreated N2aSW cells:   - ↓PINK1, Beclin-1, LC3B mRNA/protein - ↑TRPML1, STAT3 mRNA/protein |
| [84] | Cells | SK-N-AS (human neuroblastoma cells) | RT-qPCR | Pre-miR-423 vs negative control-transfected cells:   - ↓ IDH2 mRNA (59.33% and 60.19% of control in two independent experiments; p < 0.05) |
|  |  | HepG2 (human hepatocellular carcinoma) | RT-qPCR | Pre-miR-98 vs negative control-transfected cells:   - ↓ IDH2 mRNA (70.03% and 43.98% of control in two independent experiments; p < 0.05) |
| [86] | Cells | Kunming mice:  n=6 normal  n=6 sham  n=66 successfully modeled AD mice after bilateral hippocampal Aβ1–40 injection, divided into 11 groups (n=6/group) | RT-qPCR | AD-mice derived osteoblasts:   - AD + naringin vs AD + PBS: ↓ miR-96-5p (p < 0.05) - AD + miR-96-5p inhibitor vs AD + inhibitor-NC: ↓ miR-96-5p (p < 0.05), ↑ Foxo1, ↓Lcn-2 - AD + miR-96-5p mimic vs AD + miR-96-5p mimic-NC: ↓ Foxo1, ↑Lcn-2 |
|  | Brain |  | N/A | Hippocampus of AD mice:   - AD + miR-96-5p inhibitor vs AD + inhibitor-NC:   - Reduced OH- and MDA   - Increased SOD, CAT and GSH-Px - AD + sh-Lcn2 vs AD + sh-NC: Reduced mitochondrial structural damage **(qualitative assessment)** - AD + oe-Foxo1 vs oe-NC:   - Reduced mitochondrial structural damage **(qualitative assessment)**   - Reduced OH- and MDA   - Increased SOD, CAT and GSH-Px   - ↓ Lcn2 |

**^e^** Studies marked with a superscript ‘e’ included additional experimental validation directly assessing the reported ncRNA or its proposed regulatory interaction which was accordingly included in the ‘Fundamental’ section of the table.

**Notes:** Table 2 synthesizes the tissue sample, experimental model, RNA quantification method and mitochondrially-relevant results of microRNA-focused studies. Studies using human tissue like CSF, serum, plasma, blood or human omics datasets were termed ‘translational’, while in vitro and in vivo studies were referred to as ‘fundamental’. The first part of the table summarizes translational data while the second part of the table summarizes fundamental experimental findings. For those studies which featured both translational and fundamental results, the findings were placed in the respective table segment. The downregulation and upregulation of ncRNAs, other genes and proteins are represented using downward arrows (↓) and upward arrows (↑). The RNA quantification column indicates only experimental RNA expression assessment methods. N/A indicates that no experimental RNA quantification methods were reported or relevant to the study design.

**Abbreviations:** 3xTg-AD mice, Triple-transgenic Alzheimer’s disease model mice; AD, Alzheimer’s disease; aMCI, Aβ-positive amnestic mild cognitive impairment; APP, β-amyloid precursor protein; ATP, adenosine triphosphate; AuNPs, gold nanoparticles; Aβ, amyloid-beta; ceRNA, competing endogenous RNA; CSF, cerebrospinal fluid; DEmiRNA/DEmiRNAs, differentially expressed microRNA(s); EV, extracellular vesicles; HCN, adult hippocampal neural progenitor cells; hiPSC, human induced pluripotent stem cell; hNSC, human neural stem cell; iPSC, induced pluripotent stem cell; lncRNA, long non-coding RNA; MAP, Rush Memory and Aging Project; mAPP, mutant APP; MCI, mild cognitive impairment; miR/miRNA, microRNA; MMP, mitochondrial membrane potential; mRNA, messenger RNA; N/A, not applicable; ncRNA, non-coding RNA; NDAN, nondemented patients with Alzheimer's neuropathology; PDAD, prodromal Alzheimer's disease; PSAD, presymptomatic Alzheimer's disease; RNA-seq, RNA sequencing; ROS, reactive oxygen species; RT-qPCR, reverse transcription quantitative polymerase chain reaction; SAMP8, senescence-accelerated mouse prone 8; WT, wild-type; PBS, phosphate-buffered saline; CeO₂, cerium dioxide; DRP, DNA–RNA hybrid chains; MA, mitochondrial aptamers; DNA, deoxyribonucleic acid; RNA, ribonucleic acid; FAM, fluorescein amidite; IVIS, in vivo imaging system; NC, negative control; MDA, malondialdehyde; SOD, superoxide dismutase; CAT, catalase; GSH-Px, glutathione peroxidase; shRNA, short hairpin RNA; oe, overexpression, RSL3, RAS-selective lethal 3; log2FC, log2 fold change; GO, Gene Ontology.

**Gene and protein symbols:** AIF, apoptosis-inducing factor; BAD, Bcl-2-associated death promoter; BAX, BCL2-associated X protein; BCL-2, B-cell lymphoma 2; Dicer1, double-stranded RNA-specific endoribonuclease 1; DRP1, dynamin-related protein 1; FIS1, mitochondrial fission protein 1; GABARAPL1, GABA type A receptor-associated protein-like 1; GSK-3β, glycogen synthase kinase-3 beta; GSK3, glycogen synthase kinase 3; HIBCH, 3-hydroxyisobutyryl-CoA hydrolase; iNOS, inducible nitric oxide synthase; Klf4, Krüppel-like factor 4; LC3B, microtubule-associated protein 1 light chain 3B; MAP1-LC3B, microtubule-associated proteins 1A/1B light chain 3B; MDH2, malate dehydrogenase 2; MFN1, mitofusin 1; MFN2, mitofusin 2; MGME1, mitochondrial genome maintenance exonuclease 1; Miro2, mitochondrial Rho GTPase 2; NDUFB10, NADH:ubiquinone oxidoreductase subunit B10; NDUFS1, NADH:ubiquinone oxidoreductase core subunit S1; NDUFS2, NADH:ubiquinone oxidoreductase core subunit S2; NDUFS3, NADH:ubiquinone oxidoreductase core subunit S3; NDUFS8, NADH:ubiquinone oxidoreductase core subunit S8; NDUFV1, NADH:ubiquinone oxidoreductase core subunit V1; NLRP3, NLR family pyrin domain-containing protein 3; NRF1, nuclear respiratory factor 1; NRF2, nuclear factor erythroid 2-related factor 2; OPA1, optic atrophy 1 gene; Parkin, E3 ubiquitin-protein ligase parkin; PDE4A, phosphodiesterase 4A; PDHA1, pyruvate dehydrogenase E1 subunit alpha 1; PGC-1α, peroxisome proliferator-activated receptor-gamma coactivator 1-alpha; PINK1, PTEN-induced putative protein kinase 1; PSEN1, presenilin 1; RTN3, reticulon 3 gene; SDHB, succinate dehydrogenase complex iron sulfur subunit B; SIRT1, sirtuin 1; SOCS6, suppressor of cytokine signalling 6; STAT3, signal transducer and activator of transcription 3; TFEB, transcription factor EB; TIMM13, translocase of the inner mitochondrial membrane 13; TNF-α, tumor necrosis factor alpha; TOMM20, translocase of the outer mitochondrial membrane 20; TRPML1, transient receptor potential mucolipin-1; UQCRFS1, ubiquinol-cytochrome c reductase Rieske iron-sulfur polypeptide 1; UTR, untranslated region; VDAC1, voltage-dependent anion channel 1, IDH2, isocitrate dehydrogenase 2, Beclin1, beclin 1; FOXO1, forkhead box O1; LCN2, lipocalin 2, ATP5E, ATP synthase F1 subunit epsilon; NDUFB9, NADH:ubiquinone oxidoreductase subunit B9; NDUFA9, NADH:ubiquinone oxidoreductase subunit A9; COX4I1, cytochrome c oxidase subunit 4I1.

**Table 3** Long non-coding RNA dysregulation and mitochondrial dysfunction in AD: overview of translational and fundamental studies

| **LncRNA - Translational Studies** | | | | |
| --- | --- | --- | --- | --- |
| Reference | Tissue sample | Subjects or experimental model | RNA quantification  method | Results |
| [46]^e^ | Brain | GEO GSE4757 (n=10 controls, n=10 AD human cortices)  GEO GSE16759 (n=4 controls, n=4 AD human samples - parietal lobe cortex) | N/A | GSE4757, AD vs controls:   - ↓ WT1-AS (p = 0.0082) - ↓ SIX4 (p = 0.0055)   GSE16759, AD vs controls:   - ↑ miR-375 (p = 0.0438) |
| [75]^e^ | Brain | n=8 controls (5 female, 3 male)  n=8 AD patients (4 female, 4 male, Braak stages IV-VI) | RNAscope ISH; RT-qPCR; Northern blot | AD vs age- and sex-matched controls:   - ↓ lncMtDloop in PFC (p = 0.0057) - ↓ lncMtDloop in hippocampal CA1 (p = 0.0076) - ↓ lncMtDloop in dentate gyrus, trend only (p = 0.063) - ↓ lncMtDloop by RT-qPCR in PFC (p = 0.0031) and hippocampus (p = 0.0027) |
| [76]^e^ | CSF  Serum | n=16 probable AD patients (7 males and 9 females, 70.3 ± 8.8 years)  n=18 healthy individuals (8 males and 10 females, 68.3 ± 9.6 years) | RT-qPCR | AD patients vs healthy controls:   - ↑ lncRNA-ATB in CSF (p < 0.001) - ↑ lncRNA-ATB in serum (p < 0.05) |
| **LncRNA - Fundamental Studies** | | | | |
| Reference | Tissue sample | Subjects or experimental model | RNA quantification methods | Results |
| [45] | Cells | SH-SY5Y cells treated with Aβ25-35  human primary neurons treated with Aβ25-35 | RT-qPCR | Aβ25-35-treated vs untreated SH-SY5Y and human primary neurons:   - ↑ SNHG1 (20 μM: p < 0.001) - decreased MMP   Aβ25-35 + si-SNHG1 vs Aβ25-35 + si-NC:   - increased MMP and decreased apoptosis   Mechanistic validation:   - SNHG1 knockdown ↑ miR-137 (p < 0.01) - SNHG1 sponges miR-137; miR-137 directly targets KREMEN1 - miR-137 inhibition or KREMEN1 overexpression partially reversed the protective effects of SNHG1 knockdown |
| [46] | Cells | SH-SY5Y cells treated with Aβ_25-35_ | RT-qPCR | Aβ25-35-treated vs untreated SH-SY5Y cells:   - ↑ miR-375 (p < 0.05) - ↓ WT1-AS and SIX4 (p < 0.05)   Aβ25-35 + miR-375 mimic vs Aβ25-35 + mimic-NC:   - ↓ SIX4 mRNA and protein (p < 0.05) - decreased MMP and ATP production - increased oxidative stress and apoptosis   Mechanistic validation:   - miR-375 directly binds SIX4 mRNA - WT1 transcriptionally promotes miR-375 expression |
|  | Brain | Male BALB/c mice, Aβ25-35-induced AD model  n = 10/group | RT-qPCR | Aβ25-35 AD-model vs sham mice:   - ↑ miR-375 (p < 0.05) - ↓ WT1-AS and SIX4; ↑ WT1   AD + WT1-AS overexpression vs AD + oe-NC:   - ↓ miR-375 (p < 0.05) - ↑ SIX4 (p < 0.05) - decreased oxidative stress and neuronal apoptosis |
| [47] | Cells | PC12 cells treated with Aβ_25-35_ | RT-qPCR | Aβ25-35-treated vs untreated PC12 cells:   - ↑ H19 and ↓ miR-129 (p < 0.05) - ↑ HMGB1 (p < 0.05)   Aβ25-35 + si-H19 vs Aβ25-35 + si-NC:   - ↑ miR-129 and ↓ HMGB1 (p < 0.05) - decreased ROS and increased MMP (p < 0.05)   Mechanistic validation:   - H19 directly binds/sponges miR-129 - miR-129 directly targets the HMGB1 3′UTR |
|  | Brain | BALB/c male mice injected with Aβ25-35 (intraventricular)  n = 10/group | RT-qPCR | Aβ25-35 AD-model vs sham mice:   - ↑ H19 and ↓ miR-129 - ↑ HMGB1 |
| [49] | Cells | hiPSC-derived glutamatergic forebrain neurons from PSEN1-mutant patients (L150P, A79V) and isogenic controls (fibroblast-derived) | Poly(A) RNA-seq | A79V and L150P PSEN1-mutant neurons vs respective gene-corrected isogenic controls:   - ↓ MEG3 (A79V: L2FC = -5.88, Padj = 1.47 × 10⁻²⁹; L150P: L2FC = -6.31, Padj = 2.75 × 10⁻⁹⁴) - ↓ LOXL1-AS1 - ↓ A2M-AS1 |
| [75] | Cells | WT and 3xTg-AD mouse-derived primary hippocampal neuron cultures | RNAscope ISH; RT-qPCR; Northern blot | 3xTg-AD vs WT primary hippocampal neurons:   - ↓ lncMtDloop (RT-qPCR: p = 0.0075)   3xTg-AD + LV-lncMtDloop vs 3xTg-AD + LV-control:   - ↑ mtRNA (p = 0.0363) - ↑ mitochondrial genome-encoded OXPHOS genes/proteins - ↓ Drp1 protein (p = 0.0352) - ↑ Opa1 protein (p = 0.0296) - increased basal and reserve mitochondrial respiration |
|  | Brain | 3xTg-AD mice  Wild-type mice | RNAscope ISH; RT-qPCR; Northern blot | 12-month-old 3xTg-AD vs age-matched WT mice:   - ↓ lncMtDloop in PFC and hippocampus (RT-qPCR: p = 0.0059 and p = 0.0082, respectively)   3xTg-AD + AAV-lncMtDloop vs 3xTg-AD + AAV-control:   - restored mitochondrial ultrastructure and morphology in PFC and hippocampus - ↑ PINK1, Parkin and LC3B   Mechanistic validation:   - lncMtDloop directly interacts with TFAM and promotes its recruitment to mtDNA promoters, increasing mitochondrial transcription |
| [76] | Cells | neuronal PC12 cells treated with Aβ _25-35_ | RT-qPCR | Aβ25-35-treated vs untreated PC12 cells:   - ↑ lncRNA-ATB (p < 0.001) - increased ROS - decreased MMP   Aβ25-35 + sh-ATB vs Aβ25-35 + sh-NC:   - ↓ lncRNA-ATB (p < 0.01) - decreased ROS - increased MMP   sh-ATB vs sh-NC:   - ↑ miR-200 (p < 0.01)   Aβ25-35 + sh-ATB + miR-200 inhibitor vs Aβ25-35 + sh-ATB + inhibitor NC:   - increased ROS - decreased MMP   miR-200 mimic vs mimic NC:   - ↓ ZNF217 |
| [77] | Brain | APP/PS1 mice  5xFAD mice | RT-qPCR | APP/PS1 and 5xFAD mice >6 months vs age-matched WT:   - ↑ MIR600HG (p < 0.05; age-dependent)   APP/PS1 + AAV-shMIR600HG vs untreated APP/PS1:   - increased cytochrome c oxidase activity |
|  | Cells | SH-SY5Y cells | N/A | SH-SY5Y + MIR600HG overexpression vs control:   - ↑ PINK1–NEDD4L interaction - ↑ PINK1 ubiquitination - ↓ PINK1 - ↓ autophagy markers (LC3, OPTN and p62)   SH-SY5Y + shMIR600HG vs control:   - ↑ PINK1 - ↑ autophagy markers (LC3, OPTN and p62) |
| [78] | Brain (hippocampus) | APP/PS1 transgenic mice | RT-qPCR | APP/PS1 vs WT:   - ↑ lncRNA NEAT1 in older APP/PS1 mice (p < 0.01)   APP/PS1 + AAV-NEAT1 vs untreated APP/PS1:   - decreased cytochrome c oxidase activity   APP/PS1 + AAV-shNEAT1 vs untreated APP/PS1:   - increased/restored cytochrome c oxidase activity and ATP - decreased Aβ |
|  | Cells | SH-SY5Y cells | N/A | NEAT1 overexpression vs vector:   - ↑ PINK1 ubiquitination and degradation - decreased PINK1-dependent autophagy   NEAT1 knockdown vs vector:   - ↓ PINK1 ubiquitination - ↑ PINK1 and PINK1-dependent autophagy |
|  | Cells | N2A-APPsw cells | N/A | shNEAT1 vs control:   - decreased Aβ42 (p < 0.01)   shNEAT1 + siPINK1/siOPTN/siLC3 vs shNEAT1:   - reversed the reduction in Aβ42 |
| [79] | Cells | PC12 cells treated with Aβ_25-35_ | RT-qPCR | Aβ25-35 vs untreated PC12 cells:   - ↑ lncRNA BDNF-AS (p < 0.05) - ↓ BDNF   Aβ25-35 + si-BDNF-AS vs Aβ25-35 + si-NC:   - ↓ lncRNA BDNF-AS (p < 0.05) - ↑ BDNF and BCL-2; ↓ BAX and cleaved caspase-3 - increased MMP - decreased cytochrome c release and apoptosis - decreased ROS and MDA; increased SOD and CAT activity |

**^e^** Studies marked with a superscript ‘e’ included additional experimental validation directly assessing the reported ncRNA or its proposed regulatory interaction which was accordingly included in the ‘Fundamental’ section of the table.

**Notes:** Table 3 synthesizes the tissue sample, experimental model, RNA quantification method and mitochondrially-relevant results of lncRNA-focused studies. Studies using human tissue like CSF, serum, plasma, blood or human omics datasets were termed ‘translational’, while in vitro and in vivo studies were referred to as ‘fundamental’. The downregulation and upregulation of ncRNAs, other genes and proteins are represented using downward arrows (↓) and upward arrows (↑). The first part of the table summarizes translational data while the second part of the table summarizes fundamental experimental findings. For those studies which featured both translational and fundamental results, the findings were placed in the respective table segment. The RNA quantification column indicates only experimental RNA expression assessment methods. N/A indicates that no experimental RNA quantification methods were reported or relevant to the study design.

**Abbreviations:** 3xTg-AD mice, Triple-transgenic Alzheimer’s disease model mice; AD, Alzheimer’s disease; APP, β-amyloid precursor protein; ATP, adenosine triphosphate; AuNPs, gold nanoparticles; Aβ, amyloid-beta; CAT, catalase; CSF, cerebrospinal fluid; DG, dentate gyrus; GEO, Gene Expression Omnibus; hiPSC, human induced pluripotent stem cell; LC3, microtubule-associated proteins 1A/1B light chain 3; lncMtDloop, long non-coding mitochondrial displacement loop RNA; lncRNA, long non-coding RNA; lncRNA BDNF-AS, long non-coding RNA brain-derived neurotrophic factor antisense; lncRNA NEAT1, lncRNA nuclear paraspeckle assembly transcript 1; lncRNA WT1-AS, Wilms tumor 1 antisense RNA; MDA, malondialdehyde; miRNA, microRNA; MMP, mitochondrial membrane potential; mtRNA, mitochondrial RNA; N/A, not applicable; OSI, oxidative stress index; OXPHOS, oxidative phosphorylation; PFC, prefrontal cortex; Poly(A) RNA-seq, polyadenylated RNA sequencing; PSEN1, presenilin 1; RT-qPCR, reverse transcription quantitative polymerase chain reaction; SOD, superoxide dismutase; total RNA-seq, total RNA sequencing.

**Gene and protein symbols:** A2M-AS1, alpha-2-macroglobulin antisense RNA 1; BAX, BCL2-associated X protein; BCL-2, B-cell lymphoma 2; BDNF-AS, BDNF antisense RNA; DRP1, dynamin-related protein 1; HMGB1, high mobility group box 1; LC3B, microtubule-associated protein 1 light chain 3B; LOXL1-AS1, lysyl oxidase-like 1 antisense RNA 1; MIR600HG, miR-600 host gene; NEDD4L, neural precursor cell expressed, developmentally down-regulated 4-like, E3 ubiquitin protein ligase; OPA1, optic atrophy 1 gene; OPTN, optineurin; p62, sequestosome-1 (SQSTM1); Parkin, E3 ubiquitin-protein ligase parkin; PINK1, PTEN-induced putative protein kinase 1; SIX4, SIX homeobox 4; WT1, Wilms Tumor 1 gene; WT1-AS, Wilms tumor 1 antisense RNA; ZNF217, zinc finger protein 217.

**Table 4** Circular RNA dysregulation and mitochondrial dysfunction in AD: overview of translational and experimental studies

| **CircRNA – Fundamental Studies** | | | | |
| --- | --- | --- | --- | --- |
| Reference | Tissue sample | Subjects or experimental model | RNA quantification methods | Results |
| [48] | Cells | SK-N-SH and CHP212 cells treated with 20 μM Aβ exposure for 24 h | RT-qPCR | Aβ-treated vs control cells:   - ↑ circ_0049472 (p < 0.001) - ↓ miR-22-3p (p < 0.001)   Aβ + si-circ_0049472 vs Aβ + si-NC:   - ↓ circ_0049472 (p < 0.001) - ↑ miR-22-3p (p < 0.001) - ↑ BCL-2; ↓ BAX and cleaved-caspase-3 - increased/restored MMP |
| [49] | Cells | hiPSC-derived glutamatergic forebrain neurons from PSEN1-mutant patients (L150P, A79V) and isogenic controls (fibroblast-derived) | Total RNA-seq | L150P vs isogenic control:   - ↑ CDR1as/circ_0001946 (L2FC = 2.38; Padj = 2.04 × 10^-10) - ↓ UBE2A   A79V vs isogenic control:   - circ_0001946: no significant change (L2FC = 0.67; Padj = 0.37) - ↓ UBE2A |
| [80] | Cells | Primary mouse hippocampal neurons treated with Aβ1-42 | RT-qPCR | miR-647 inhibitor + sh-circ_0004381 vs sh-circ_0004381:   - ↓ miR-647 (p < 0.05) - increased ROS and cytoplasmic cytochrome c; decreased SOD - attenuated the increases in ATP and MMP - ↑ BAX/BCL-2 ratio |
|  | Cells | Microglial BV2 cells treated with lipopolysaccharide | N/A | LPS + sh-circ_0004381 vs LPS + sh-NC:   - ↓ iNOS and COX-2 - decreased M1 and increased M2 polarization - decreased IL-1β, IL-6 and TNF-α |
|  | Brain | Male APPswe/PS1dE9 transgenic mice | N/A | APPswe/PS1dE9 vs non-transgenic controls:   - ↑ circ_0004381 and ↓ miR-647 (p < 0.05)   APPswe/PS1dE9 + sh-circ_0004381 vs untreated APPswe/PS1dE9:   - ↓ circ_0004381 and ↑ miR-647 (p < 0.05) - decreased ROS |

**Notes**: Table 4 synthesizes the tissue sample, experimental model, RNA quantification method and mitochondrially-relevant results of 3 circRNA-focused studies. No eligible circRNA studies using patient-derived data were identified after the screening process. All circRNA evidence was derived from experimental in vitro and in vivo studies, which we refer to as ‘fundamental studies’. The downregulation and upregulation of ncRNAs, other genes and proteins are represented using downward arrows (↓) and upward arrows (↑). The RNA quantification column indicates only experimental RNA expression assessment methods. N/A indicates that no experimental RNA quantification methods were reported or relevant to the study design.

**Abbreviations:** AD, Alzheimer’s disease; APP, β-amyloid precursor protein; ATP, adenosine triphosphate; Aβ, amyloid-beta; circRNA, circular RNA; hiPSC, human induced pluripotent stem cell; MMP, mitochondrial membrane potential; N/A, not applicable; ncRNA, non-coding RNA; Poly(A) RNA-seq, polyadenylated RNA sequencing; RNA-seq, RNA sequencing; ROS, reactive oxygen species; RT-qPCR, reverse transcription quantitative polymerase chain reaction; SOD, superoxide dismutase; total RNA-seq, total RNA sequencing.

**Gene and protein symbols:** BAX, BCL2-associated X protein; BCL-2, B-cell lymphoma 2; COX-2, cyclooxygenase-2; iNOS, inducible nitric oxide synthase; PSEN1, presenilin 1; UBE2A, ubiquitin-conjugating enzyme E2A.

**References**

45.	Wang H, Lu B, Chen J (2019) Knockdown of lncRNA SNHG1 attenuated Aβ25-35-inudced neuronal injury via regulating KREMEN1 by acting as a ceRNA of miR-137 in neuronal cells. Biochem Biophys Res Commun 518:438–444. https://doi.org/10.1016/j.bbrc.2019.08.033

46.	Wang Q, Ge X, Zhang J, Chen L (2020) Effect of lncRNA WT1-AS regulating WT1 on oxidative stress injury and apoptosis of neurons in Alzheimer’s disease via inhibition of the miR-375/SIX4 axis. Aging 12:23974–23995. https://doi.org/10.18632/aging.104079

47.	Zhang Y-Y, Bao H-L, Dong L-X, Liu Y, Zhang G-W, An F-M (2021) Silenced lncRNA H19 and up-regulated microRNA-129 accelerates viability and restrains apoptosis of PC12 cells induced by Aβ(25-35) in a cellular model of Alzheimer’s disease. Cell Cycle Georget Tex 20:112–125. https://doi.org/10.1080/15384101.2020.1863681

48.	Chen J, Xiao S, Cui X, Gao X, Wang D, Li X, Qi W, Wang B (2025) Circ_0049472 downregulation relieves Amyloid-β-induced neuronal injury by modulating PDE4A expression via targeting miR-22-3p in Alzheimer’s disease. Metab Brain Dis 40:252. https://doi.org/10.1007/s11011-025-01652-4

49.	Corsi GI, Gadekar VP, Haukedal H, Doncheva NT, Anthon C, Ambardar S, Palakodeti D, Hyttel P, Freude K, Seemann SE, Gorodkin J (2023) The transcriptomic landscape of neurons carrying PSEN1 mutations reveals changes in extracellular matrix components and non-coding gene expression. Neurobiol Dis 178:105980. https://doi.org/10.1016/j.nbd.2022.105980

50.	Wang Y, Lian M, Zhou J, Wu S (2020) Brain Dicer1 Is Down-Regulated in a Mouse Model of Alzheimer’s Disease Via Aβ42-Induced Repression of Nuclear Factor Erythroid 2-Related Factor 2. Mol Neurobiol 57:4417–4437. https://doi.org/10.1007/s12035-020-02036-8

51.	Yuen SC, Lee SM-Y, Leung S (2022) Putative Factors Interfering Cell Cycle Re-Entry in Alzheimer’s Disease: An Omics Study with Differential Expression Meta-Analytics and Co-Expression Profiling. J Alzheimers Dis 85:1373–1398. https://doi.org/10.3233/JAD-215349

52.	Xu X, Fan S, Li J, Wu H, He J, He Y, Meng X, Shen Y (2025) Integrated Multi‐Omics Analysis and Cross‐Model Validation Reveal Mitochondrial Signatures in Alzheimer’s Disease. CNS Neurosci Ther 31:e70634. https://doi.org/10.1111/cns.70634

53.	Li H, Feng F, Xie S, Ma Y, Wang Y, Zhang F, Wu H, Huang S (2025) Identification of HIBCH and MGME1 as Mitochondrial Dynamics‐Related Biomarkers in Alzheimer’s Disease Via Integrated Bioinformatics Analysis. IET Syst Biol 19:e70018. https://doi.org/10.1049/syb2.70018

54.	Abyadeh M, Kaya A (2025) Multiomics from Alzheimer’s Brains and Mesenchymal Stem Cell-Derived Extracellular Vesicles Identifies Therapeutic Potential of Specific Subpopulations to Target Mitochondrial Proteostasis. J Cent Nerv Syst Dis 17:11795735251336302. https://doi.org/10.1177/11795735251336302

55.	Fracassi A, Marcatti M, Zolochevska O, Tabor N, Woltjer R, Moreno S, Taglialatela G (2021) Oxidative Damage and Antioxidant Response in Frontal Cortex of Demented and Nondemented Individuals with Alzheimer’s Neuropathology. J Neurosci 41:538–554. https://doi.org/10.1523/JNEUROSCI.0295-20.2020

56.	Nguyen HD, Kim W-K, Huong Vu G (2024) Molecular mechanisms implicated in protein changes in the Alzheimer’s disease human hippocampus. Mech Ageing Dev 219:111930. https://doi.org/10.1016/j.mad.2024.111930

57.	Cieślik M, Czapski GA, Wójtowicz S, Wieczorek I, Wencel PL, Strosznajder RP, Jaber V, Lukiw WJ, Strosznajder JB (2020) Alterations of Transcription of Genes Coding Anti-oxidative and Mitochondria-Related Proteins in Amyloid β Toxicity: Relevance to Alzheimer’s Disease. Mol Neurobiol 57:1374–1388. https://doi.org/10.1007/s12035-019-01819-y

58.	Kim SH, Choi KY, Park Y, McLean C, Park J, Lee JH, Lee K-H, Kim BC, Huh YH, Lee KH, Song WK (2021) Enhanced Expression of microRNA-1273g-3p Contributes to Alzheimer’s Disease Pathogenesis by Regulating the Expression of Mitochondrial Genes. Cells 10:2697. https://doi.org/10.3390/cells10102697

59.	Kim SJ, Russell AE, Wang W, Gemoets DE, Sarkar SN, Simpkins JW, Brown CM (2022) miR-146a Dysregulates Energy Metabolism During Neuroinflammation. J Neuroimmune Pharmacol 17:228–241. https://doi.org/10.1007/s11481-021-09999-y

60.	Huang H-Z, Ai W-Q, Wei N, Zhu L-S, Liu Z-Q, Zhou C-W, Deng M-F, Zhang W-T, Zhang J-C, Yang C-Q, Hu Y-Z, Han Z-T, Zhang H-H, Jia J-J, Wang J, Liu F-F, Li K, Xu Q, Yuan M, Man H, Guo Z, Lu Y, Shu K, Zhu L-Q, Liu D (2024) Senktide blocks aberrant RTN3 interactome to retard memory decline and tau pathology in social isolated Alzheimer’s disease mice. Protein Cell 15:261–284. https://doi.org/10.1093/procel/pwad056

61.	Han AR, Moon TK, Kang IK, Yu DB, Kim Y, Byon C, Park S, Kim HL, Lee KJ, Lee H, Woo H-N, Kim SW (2024) Integrative analysis of microRNA-mediated mitochondrial dysfunction in hippocampal neural progenitor cell death in relation with Alzheimer’s disease. BMB Rep 57:281–286. https://doi.org/10.5483/BMBRep.2023-0167

62.	Xu W, Su X, Qin J, Jin Y, Zhang N, Huang S (2024) Identification of Autophagy-Related Biomarkers and Diagnostic Model in Alzheimer’s Disease. Genes 15:1027. https://doi.org/10.3390/genes15081027

63.	Woo H-N, Park S, Kim HL, Jung M-K, Pack C-G, Park J, Cho Y, Jo D-G, Kim DK, Mook-Jung I, Kim SW, Lee H (2021) miR-351-5p/Miro2 axis contributes to hippocampal neural progenitor cell death via unbalanced mitochondrial fission. Mol Ther Nucleic Acids 23:643–656. https://doi.org/10.1016/j.omtn.2020.12.014

64.	Vijayan M, Reddy PH (2024) Unveiling the Role of Novel miRNA PC-5P-12969 in Alleviating Alzheimer’s Disease. J Alzheimers Dis 98:1329–1348. https://doi.org/10.3233/JAD-231281

65.	Ramesh M, Govindaraju T (2025) MiR-7a–Klf4 axis as a regulator and therapeutic target of neuroinflammation and ferroptosis in Alzheimer’s disease. NAR Mol Med 2:ugaf022. https://doi.org/10.1093/narmme/ugaf022

66.	Wang Y, Sun X, He B, Yu S (2025) Ginsenoside Rg1 Downregulates miR-9-5p Expression to Modulate SIRT1-Mediated Mitochondrial Dysfunction and Ameliorate Alzheimer’s Disease. Mol Neurobiol 62:13044–13059. https://doi.org/10.1007/s12035-025-05073-3

67.	Rao S, Madhu LN, Babu RS, Shankar G, Kotian S, Nagarajan A, Upadhya R, Narvekar E, Cai JJ, Shetty AK (2025) Extracellular vesicles from hiPSC-derived NSCs protect human neurons against Aβ-42 oligomers induced neurodegeneration, mitochondrial dysfunction and tau phosphorylation. Stem Cell Res Ther 16:191. https://doi.org/10.1186/s13287-025-04324-3

68.	Liang C, Mu Y, Tian H, Wang D, Zhang S, Wang H, Liu Y, Di C (2021) MicroRNA-140 silencing represses the incidence of Alzheimer’s disease. Neurosci Lett 758:135674. https://doi.org/10.1016/j.neulet.2021.135674

69.	Liu J, Zuo X, Han J, Dai Q, Xu H, Liu Y, Cui S (2020) MiR-9-5p inhibits mitochondrial damage and oxidative stress in AD cell models by targeting GSK-3β. Biosci Biotechnol Biochem 84:2273–2280. https://doi.org/10.1080/09168451.2020.1797469

70.	Zhang R, Zhou H, Jiang L, Mao Y, Cui X, Xie B, Cui D, Wang H, Zhang Q, Xu S (2016) MiR-195 dependent roles of mitofusin2 in the mitochondrial dysfunction of hippocampal neurons in SAMP8 mice. Brain Res 1652:135–143. https://doi.org/10.1016/j.brainres.2016.09.047

71.	Zhang L, Fang Y, Zhao X, Zheng Y, Ma Y, Li S, Huang Z, Li L (2021) miR-204 silencing reduces mitochondrial autophagy and ROS production in a murine AD model via the TRPML1-activated STAT3 pathway. Mol Ther - Nucleic Acids 24:822–831. https://doi.org/10.1016/j.omtn.2021.02.010

72.	Garcia G, Pinto S, Cunha M, Fernandes A, Koistinaho J, Brites D (2021) Neuronal Dynamics and miRNA Signaling Differ between SH-SY5Y APPSwe and PSEN1 Mutant iPSC-Derived AD Models upon Modulation with miR-124 Mimic and Inhibitor. Cells 10:2424. https://doi.org/10.3390/cells10092424

73.	Garcia G, Fernandes A, Stein F, Brites D (2022) Protective Signature of IFNγ-Stimulated Microglia Relies on miR-124-3p Regulation From the Secretome Released by Mutant APP Swedish Neuronal Cells. Front Pharmacol 13:833066. https://doi.org/10.3389/fphar.2022.833066

74.	Kumar S, Reddy AP, Yin X, Reddy PH (2019) Novel MicroRNA-455-3p and its protective effects against abnormal APP processing and amyloid beta toxicity in Alzheimer’s disease. Biochim Biophys Acta BBA - Mol Basis Dis 1865:2428–2440. https://doi.org/10.1016/j.bbadis.2019.06.006

75.	Xiong W, Xu K, Sun JK-L, Liu S, Zhao B, Shi J, Herrup K, Chow H-M, Lu L, Li J (2024) The mitochondrial long non-coding RNA lncMtloop regulates mitochondrial transcription and suppresses Alzheimer’s disease. EMBO J 43:6001–6031. https://doi.org/10.1038/s44318-024-00270-7

76.	Wang J, Zhou T, Wang T, Wang B (2018) Suppression of lncRNA-ATB prevents amyloid-β-induced neurotoxicity in PC12 cells via regulating miR-200/ZNF217 axis. Biomed Pharmacother 108:707–715. https://doi.org/10.1016/j.biopha.2018.08.155

77.	Liu Q, Ling Z, Zhang J, Yu H, Wang Y, Xue Y, Wang C, Zhao J, Cao J, Duan S, Zhao J (2022) lncRNA MIR600HG Knockdown Alleviates Cognitive Impairment in Alzheimer’s Disease Through NEDD4L Mediated PINK1 Degradation. J Alzheimer’s Dis 85:1783–1794. https://doi.org/10.3233/JAD-215194

78.	Huang Z, Zhao J, Wang W, Zhou J, Zhang J (2020) Depletion of LncRNA NEAT1 Rescues Mitochondrial Dysfunction Through NEDD4L-Dependent PINK1 Degradation in Animal Models of Alzheimer’s Disease. Front Cell Neurosci 14:28. https://doi.org/10.3389/fncel.2020.00028

79.	Guo C-C, Jiao C, Gao Z-M (2018) Silencing of LncRNA BDNF-AS attenuates Aβ_25-35_ -induced neurotoxicity in PC12 cells by suppressing cell apoptosis and oxidative stress. Neurol Res 40:795–804. https://doi.org/10.1080/01616412.2018.1480921

80.	Li N, Zhang D, Guo H, Yang Q, Li P, He Y (2022) Inhibition of circ_0004381 improves cognitive function via miR-647/PSEN1 axis in an Alzheimer disease mouse model. J Neuropathol Exp Neurol 82:84–92. https://doi.org/10.1093/jnen/nlac108

81.	Wang G, Shen X, Song X, Wang N, Wo X, Gao Y (2023) Protective mechanism of gold nanoparticles on human neural stem cells injured by β-amyloid protein through miR-21–5p/SOCS6 pathway. NeuroToxicology 95:12–22. https://doi.org/10.1016/j.neuro.2022.12.011

82.	Wang Y, Xiao Z, Yin H, Ren Z, Ma X, Wang Y, Zhang Y, Fu X, Zhang F, Zeng L (2024) miRNA375-3p/rapamycin mediates the mTOR pathway by decreasing PS1, enhances microglial cell activity to regulate autophagy in Alzheimer’s disease. Heliyon 10:. https://doi.org/10.1016/j.heliyon.2024.e37589

83.	Tan MS, Cheah P-L, Chin A-V, Looi L-M, Chang S-W (2023) Differential Expression Analysis of Blood MicroRNA in Identifying Potential Genes Relevant to Alzheimer’s Disease Pathogenesis, Using an Integrated Bioinformatics and Machine Learning Approach. Appl Sci 13:3071. https://doi.org/10.3390/app13053071

84.	Cogswell JP, Ward J, Taylor IA, Waters M, Shi Y, Cannon B, Kelnar K, Kemppainen J, Brown D, Chen C, Prinjha RK, Richardson JC, Saunders AM, Roses AD, Richards CA (2008) Identification of miRNA Changes in Alzheimer’s Disease Brain and CSF Yields Putative Biomarkers and Insights into Disease Pathways. J Alzheimers Dis 14:27–41. https://doi.org/10.3233/JAD-2008-14103

85.	Li W, Dong M-J, Dai H, Lu S, Luo R, Cao J, Zhang F, Mei L, Yu J (2025) Application of mitochondrial miRNA-204 nanoprobes in Alzheimer’s disease treatment by clearing reactive oxygen species-mediated autophagy. Chin Chem Lett 36:110614. https://doi.org/10.1016/j.cclet.2024.110614

86.	Wu B-W, Guo J-D, Wu M-S, Liu Y, Lu M, Zhou Y-H, Han H-W (2020) Osteoblast-Derived Lipocalin-2 Regulated by miRNA-96-5p/Foxo1 Advances the Progression of Alzheimer’s Disease. Epigenomics 12:1501–1513. https://doi.org/10.2217/epi-2019-0215
